# CNVkit: Genome-Wide Copy Number Detection and Visualization from Targeted DNA Sequencing

**DOI:** 10.1371/journal.pcbi.1004873

**Published:** 2016-04-21

**Authors:** Eric Talevich, A. Hunter Shain, Thomas Botton, Boris C. Bastian

**Affiliations:** 1 Department of Dermatology, University of California, San Francisco, San Francisco, California, United States of America; 2 Department of Pathology, University of California, San Francisco, San Francisco, California, United States of America; 3 Helen Diller Family Comprehensive Cancer Center, University of California, San Francisco, San Francisco, California, United States of America

## Abstract

Germline copy number variants (CNVs) and somatic copy number alterations (SCNAs) are of significant importance in syndromic conditions and cancer. Massively parallel sequencing is increasingly used to infer copy number information from variations in the read depth in sequencing data. However, this approach has limitations in the case of targeted re-sequencing, which leaves gaps in coverage between the regions chosen for enrichment and introduces biases related to the efficiency of target capture and library preparation. We present a method for copy number detection, implemented in the software package CNVkit, that uses both the targeted reads and the nonspecifically captured off-target reads to infer copy number evenly across the genome. This combination achieves both exon-level resolution in targeted regions and sufficient resolution in the larger intronic and intergenic regions to identify copy number changes. In particular, we successfully inferred copy number at equivalent to 100-kilobase resolution genome-wide from a platform targeting as few as 293 genes. After normalizing read counts to a pooled reference, we evaluated and corrected for three sources of bias that explain most of the extraneous variability in the sequencing read depth: GC content, target footprint size and spacing, and repetitive sequences. We compared the performance of CNVkit to copy number changes identified by array comparative genomic hybridization. We packaged the components of CNVkit so that it is straightforward to use and provides visualizations, detailed reporting of significant features, and export options for integration into existing analysis pipelines. CNVkit is freely available from https://github.com/etal/cnvkit.

This is a *PLoS Computational Biology* software paper.

## Introduction

Copy number changes are a useful diagnostic indicator for many diseases, including cancer. The gold standard for genome-wide copy number is array comparative genomic hybridization (array CGH) [1, 2]. More recently, methods have been developed to obtain copy number information from whole-genome sequencing data ([3]; reviewed by [4]). For clinical use, sequencing of genome partitions, such as the exome or a set of disease-relevant genes, is often preferred to enrich for regions of interest and sequence them at higher coverage to increase the sensitivity for calling variants [5]. Tools have been developed for copy number analysis of these datasets, as well, including CNVer [6], ExomeCNV [7], exomeCopy [8], CONTRA [9], CoNIFER [10], ExomeDepth [11], VarScan 2 [12], XHMM [13], ngCGH [14], EXCAVATOR [15], CANOES [16], PatternCNV [17], CODEX [18], and recent versions of Control-FREEC [19] and cn.MOPS [20]. However, these approaches do not use the sequencing reads from intergenic and, usually, intronic regions, limiting their potential to infer copy number across the genome.

During the target enrichment, targeted regions are captured by hybridization; however, a significant quantity of off-target DNA remains in the library, and this DNA is sequenced and represents a considerable portion of the reads. Thus, off-target reads provide a very low-coverage sequencing of the whole genome, in addition to the high-coverage sequencing obtained in targeted regions. While the off-target reads alone do not provide enough coverage to call single-nucleotide variants (SNVs) and other small variants, they can provide useful information on copy number at a larger scale, as recently demonstrated by cnvOffSeq [21] and CopywriteR [22].

We developed a computational method for analysis of copy number variants and alterations in targeted DNA sequencing data that we packaged into a software toolkit. This toolkit, called CNVkit, implements a pipeline for CNV detection that takes advantage of both on– and off-target sequencing reads and applies a series of corrections to improve accuracy in copy number calling. We compare binned read depths in on– and off-target regions and find that they provide comparable estimates of copy number, albeit at different resolutions. We evaluate several bias correction algorithms to reduce the variance among binned read counts unlikely to be driven by true copy number changes. Finally, we compare copy ratio estimates by the CNVkit method and two competing CNV callers to those of array CGH, and find that CNVkit most closely agrees with array CGH. In summary, we demonstrate that both on– and off-target reads can be combined to provide highly accurate and reliable copy ratio estimates genome-wide, maximizing the copy number information obtained from targeted sequencing.

## Design and Implementation

We implemented CNVkit as a Python 2.7 software package comprising a command-line program, cnvkit.py, and reusable library, cnvlib.

### Software pipeline

**Fig 1:**
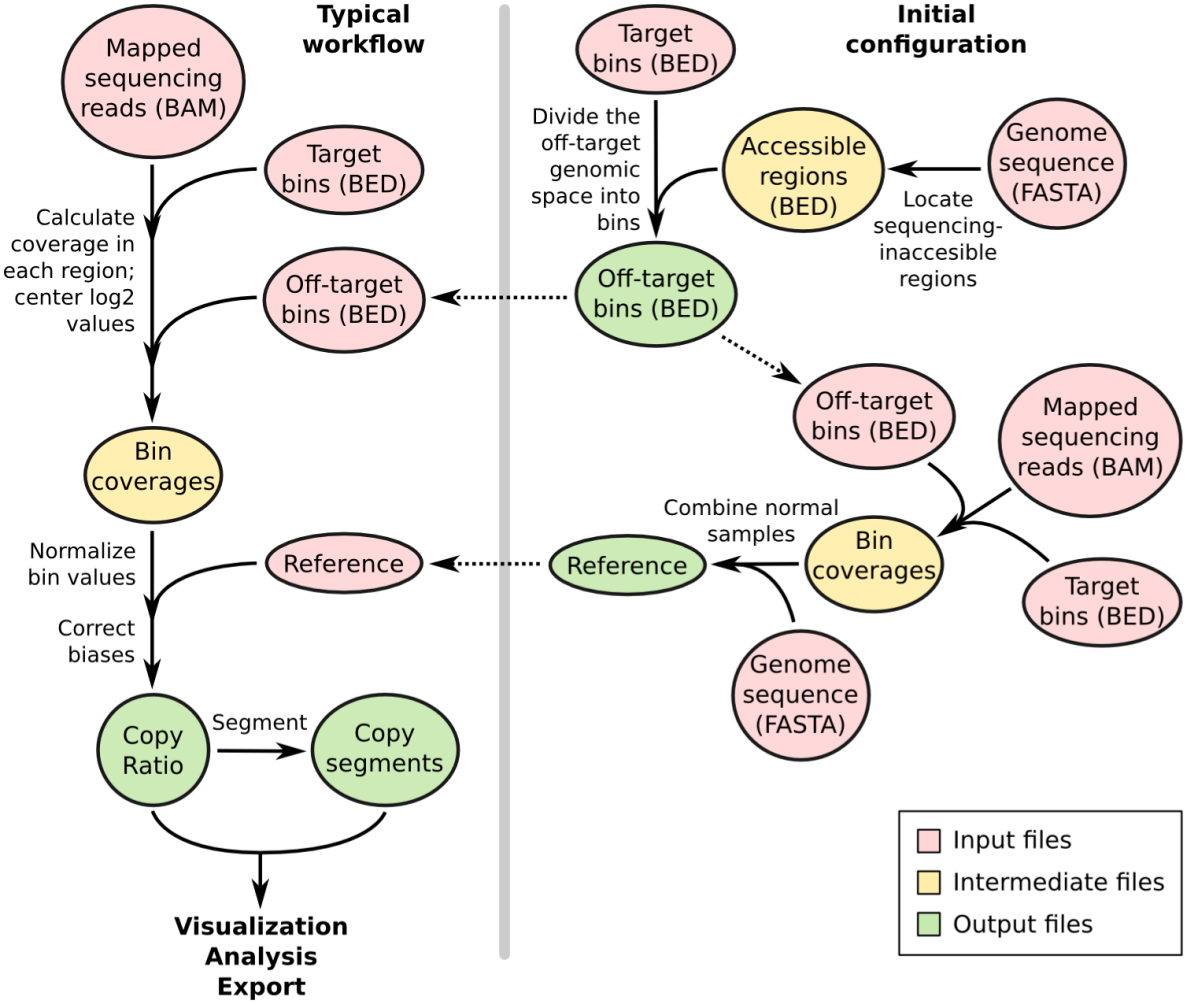
CNVkit workflows. The target and off-target bin BED files and reference file are constructed once for a given platform and can be used to process many samples sequenced on the same platform, as shown in the workflow on the left. Steps to construct the off-target bins are shown at the top-right, and construction of the reference is shown at the lower-right.

The input to the program is one or more DNA sequencing read alignments in BAM format [23] and the capture bait locations or a pre-built “reference” file (Fig 1). All additional data files used in the workflow, such as GC content and the location of sequence repeats, can be extracted from user-supplied genome sequences in FASTA format using scripts included with the CNVkit distribution. The workflow is not restricted to the human genome, and can be run equally well on other genomes.

CNVkit uses both the on-target reads and the nonspecifically captured off-target reads to calculate log_2_ copy ratios across the genome for each sample. Briefly, off-target bins are assigned from the genomic positions between targeted regions, with the average off-target bin size being much larger than the average on-target bin to match their read counts (Table 1). Both the on– and off-target locations are then separately used to calculate the mean read depth within each interval. The on– and off-target read depths are then combined, normalized to a reference derived from control samples, corrected for several systematic biases to result in a final table of log_2_ copy ratios. A built-in segmentation algorithm can be run on the log_2_ ratio values to infer discrete copy number segments. The log_2_ ratios and segments can then be used for visualization and further analyses supported by CNVkit, exported to other formats, and used with third-party software.

**Table 1:** Binning statistics.

	TR		EX		C0902	
Statistic	on-target	off-target	on-target	off-target	on-target	off-target
Number of bins	8,216	19,434	301,249	55012	8,662	19,402
Total bin footprint (bp)	1,791,315	2,837,786,301	70,364,091	2,468,075,581	1,867,888	2,837,005,032
Mean bin size (bp)	218.0	146,021.7	233.6	44864.3	215.6	146,222.3
Min. bin size (bp)	36	10,012	114	6,000	42	10,089
1st quartile bin size (bp)	183	148,196	197	11,304	181	148,108
Median bin size (bp)	203	149,819	227	28,358	200	149,814.5
3rd quartile bin size (bp)	259	151,062	268	86,767	257	151,070
Max. bin size (bp)	397	223,781	399	134,972	398	224,678

The bins for the exome panel (EX) cover a slightly smaller total genomic footprint than the targeted panels (TR, C0902) because most introns are smaller than the minimum size allowed for off-target bins, and thus discarded from the exome bins, while the off-target bins in the targeted panels span both the introns and exons of non-targeted genes.

These steps are implemented entirely in CNVkit so that the complete workflow can be performed in a reasonable amount of time on a commodity workstation or laptop. The most computationally demanding step, read depth calculation, takes on the order of 20 minutes for an exome at 100-fold coverage or 2 minutes for a 293-gene target panel at 500-fold coverage using a single 3.7GHz CPU and a solid-state drive. Initial calculation of regional GC RepeatMasker content from the human genome takes about one minute, and all other steps complete in a few seconds at most. The implementation is designed to be memory-efficient, so that many samples can safely be run in parallel on a single machine.

### Calculation of off-target intervals

Genomic intervals for counting off-target reads are initially calculated from the genomic positions of the targeted intervals. The CNVkit antitarget command accepts a list of targeted regions, in Browser Extensible Data (BED) or GATK/Picard interval list format, and divides the off-target regions between each target into large bins, typically on the order of 100 kilobases. As an optional input, separate lists of the sequencing-accessible chromosomal regions and low-mappability regions can be used to exclude telomeres, centromeres and other sequencing-inaccessible or unmappable repetitive regions from the off-target intervals when creating the off-target bins.

Each contiguous off-target region is divided into equal-sized bins such that the average bin size within the region is as close as possible to the size specified by the user. The user can select an appropriate off-target bin size by calculating the product of the average target region size and the fold-enrichment of sequencing reads in targeted regions, such that roughly the same number of reads are mapped to on– and off-target bins on average. In an effort to maximize the number of bins, CNVkit will deviate from the user-specified bin size to fit bins into small regions, such as introns, that are restricted in size. The user can also specify a lower limit on bin size to avoid evaluating very small off-target regions, where it is expected that too few reads would be captured to give a reliable estimate of copy number. Once a satisfactory set of off-target bins have been generated and saved as a BED file, the same BED file can be reused with CNVkit for copy number analysis of other samples prepared with the same library preparation protocol and sequenced on the same platform.

### Estimation of copy number by read depth

The CNVkit coverage command computes the log_2_ mean read depth in each bin for a sample using an alignment of sequencing reads in BAM format and the positions of the on– or off-target bins in BED or interval list format. For each bin the read depths at each base pair in the bin are calculated and summed using pysam, a Python interface to samtools [23], and then divided by the size of the bin. The output is a table of the average read depths in each of the given bins log_2_-transformed and centered to the median read depth of all autosomes.

To produce the input BAM file, we recommend that an aligner such as BWA-MEM [24] be used with the option to mark secondary mappings of reads, and that PCR duplicates be flagged.

### Construction of a copy number reference

The reference command estimates the expected read depth of each on– and off-target bin across a panel of control or comparison samples to produce a reference copy-number profile that can then be used to correct other test samples. At each genomic bin, the read depths in each of the given control samples are extracted. Read-depth bias corrections (see below) are performed on each of the control samples. In each bin, a weighted average of the log_2_ read depths among the control samples is calculated to indicate bins that systematically have higher or lower coverage, and the spread or statistical dispersion of log2 read depths indicates bins that have erratic coverage so that they can be de-emphasized at the segmentation step. A single paired control sample can also be used, or, in absence of any control samples, a “generic” reference can be constructed with a log_2_ read depth and spread of 0 assigned to all bins. In all cases a “male reference” can be specified in which the expected read depth of X chromosome bins is half that of the autosomes.

Additional information can be associated with each bin for later use in bias correction and segmentation. If the user provides a FASTA file of the reference genome at this step, the GC content and repeat-masked fraction of each binned corresponding genomic region are calculated. CNVkit calculates the fraction of each bin that is masked and records this fraction in an additional column in the reference file, along with GC, average log2 read depth, and spread.

As with the target and off-target BED files, once a satisfactory reference file has been generated, it can be reused with CNVkit for copy number analysis of other similar samples sequenced with the same platform and protocol.

### Normalization of test samples to the reference

The fix command combines a single sample’s on– and off-target binned read depths, removes bins failing predefined criteria, corrects for systematic biases in bin coverage (see below), subtracts the reference log_2_ read depths, and finally median-centers the corrected copy ratios.

Each bin is then assigned a weight to be used in segmentation and plotting. Each bin’s weight is calculated according to bin size, difference from the global median coverage (if at least one control sample is provided), and the spread of normalized coverages in the control pool (if more than one control sample is provided). Finally, the overall variability of bin log2 ratio values is compared between on- and off-target bins, and the more variable of the two sets is downweighted in proportion.

### Correction of coverage biases

Read depth alone is an insufficient proxy for copy number because of systematic biases in coverage introduced during library preparation and sequencing. For example, read depth is affected by GC content, sequence complexity and the sizes of individual targeted intervals [15, 19, 25]. To account for each of these potential biases in depth of read coverage, CNVkit uses a rolling median technique to recenter each on– or off-target bin with other bins of similar GC content, repetitiveness, target size or distance from other targets, independently of genomic location.

Systematic coverage biases may be largely removed simply by normalization to a reference of one or more representative normal samples, and subsequent corrections for these biases then have relatively little effect. However, even after normalization to a pooled reference, biases in coverage typically do persist in an individual sample and must still be removed.

#### Genomic GC content

DNA regions with extreme GC content are less accessible to hybridization and amenable to amplification during library preparation [26, 27]. The degree of GC bias can vary between samples due to differences such as the quality of each sample’s DNA or efficiency of hybridization between library preparations. To remove this bias, CNVkit applies a rolling median correction (see below) to GC values on both the target and off-target bins, independently.

#### Sequence repeats

Repetitive sequences in the genome can complicate read-depth calculations, as these regions often show high variability in coverage from sample to sample [15]. This variability may be due to differences in the efficiency of the blocking step during library preparation (e.g. differences in the quantity of Cot-1 during blocking). The presence of sequence repeats serves as an indicator for regions prone to these biases.

In the reference genome sequences provided by the UCSC Genome Bioinformatics Site (http://genome.ucsc.edu/) and others, repetitive regions are masked out by RepeatMasker (http://repeatmasker.org). CNVkit calculates the proportion of each bin that is masked, similar to the method used in XHMM [13], and uses this information for bias correction. The CNVkit implementation applies the RepeatMasker correction to only the off-target bins. For most custom bait libraries, the on-target bins are much smaller, and usually are exonic, and therefore generally have no overlap with repeats. For those on-target bins that were identified as containing repeats (e.g. ~7% in our custom target panel, see Results), we found them mostly entirely covered by the repeat, leaving very few intermediate points to infer a continuous trend for correction by the rolling median.

#### Target density

**Fig 2:**
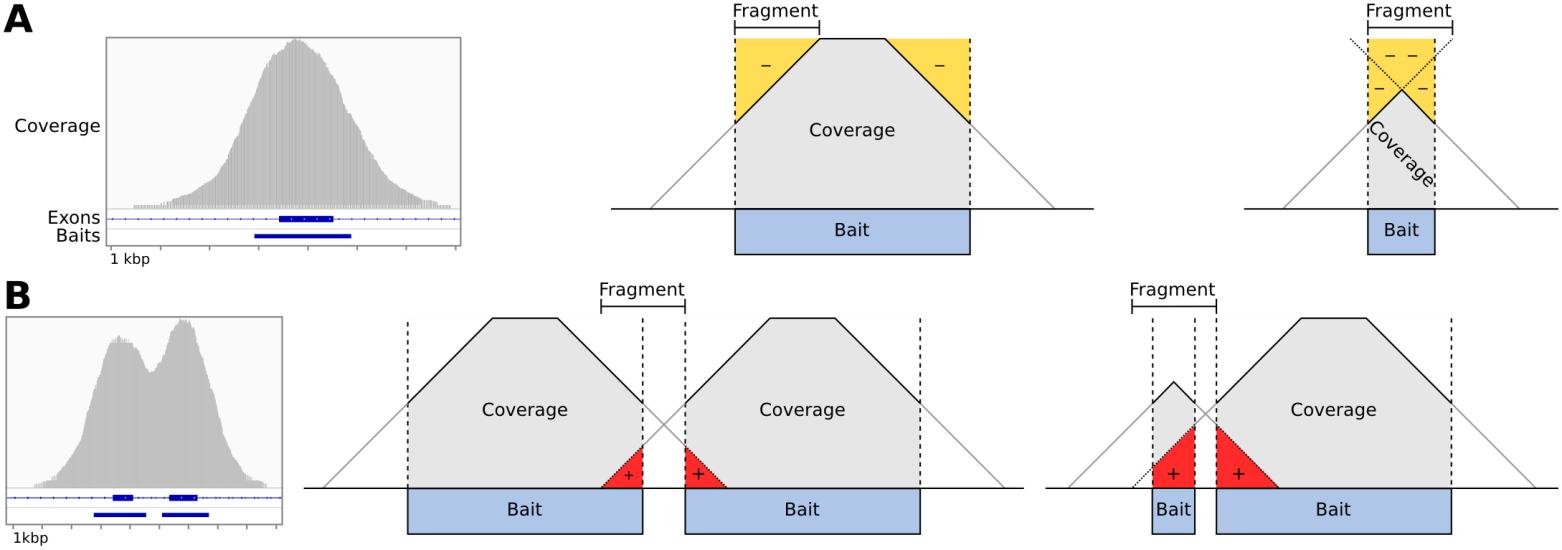
Baited region size and spacing affect read depth systematically. A: Example of typical coverage observed at a targeted exon, as viewed in IGV, and simplified geometric models of the negative coverage biases (yellow) that can occur as a function of the relative sizes of sequence fragments and the baited region. B: Coverage observed at two neighboring targeted exons, and models of the positive coverage biases (red) that can occur where intervals are separated by less than half the insert size of sequence fragments.

We observed two distortions to read depth consistently occurring at the edges of each targeted interval (Fig 2): The “shoulders” of each interval showed reduced read depth due to incomplete sequence match to the bait, creating a negative bias in the observed read depth inside the interval near each edge; this effect was greatest for short intervals (Fig 2A). Some off-target capture also occurred in the “flanks” of the baited interval due to the same mechanism. Where targets are closely spaced or adjacent, this flanking read depth may overlap with a neighboring target, creating a positive bias in its observed read depth (Fig 2B). We accounted for the negative bias at the interval “shoulders” and the positive bias in the interval “flank” regions in a single model that describes the “density” of targets around a bin.

CNVkit’s bias correction procedure needs only a monotonic function of the actual read depth bias, rather than the magnitude of the bias itself. For simplicity, we modeled the density biases as a linear decrease in read depth from inside the baited region to the same distance outside, calculated from the start and end positions of a bin and its immediate neighbors (Fig 2A and 2B). In the common case of no other targets within the window surrounding the given target, there is a one-to-one correspondence of the density value to target size. Thus, the density bias correction also accounts for the bias due to target size that has been described by others [15].

While the density bias can be significantly reduced by normalizing each sample to a reference, it may vary between samples due to differences in the insert sizes of sequence fragments introduced during the step of DNA fragmentation of the library preparation, and thus should still be accounted for even if a matched normal comparison exists. Density bias being related to the capture, CNVkit only applies this correction to the on-target bins.

#### Computational correction of biases

All of the information needed to calculate the biases at each bin is stored in the reference file. For each of the biases (GC content, repeat-masked fraction, target density), the bias value is calculated for each bin. Next, bins are sorted by bias value. A rolling median is then calculated across the bin log_2_ ratios ordered by bias value to obtain a midpoint log_2_ ratio value representing the expected bias for each bin. Finally, this value is subtracted from the original bin log_2_ ratio for the given sample to offset the observed bias. We also evaluated local regression (LOWESS) [28] and a Kaiser window function [29] in place of the rolling median to estimate the trend due to bias; all three functions produced similar fits on sample data, and we chose rolling median as the default for its simplicity and robustness.

### Segmentation and calling absolute copy number

The sample’s corrected bin-level copy ratio estimates can be segmented into discrete copy-number regions using the segment command. The bin log2 ratio values are first optionally filtered for outliers, defined as a fixed multiple of the 95th quartile in a rolling window, similar to BIC-seq [30]. The default segmentation algorithm used is circular binary segmentation (CBS) [31], via the R package PSCBS [32]. Alternatively, the HaarSeg algorithm [33] or Fused Lasso [34] can be used in place of CBS. In either case, the segmentation output is in a BED-like tabular format similar to that used for bin-level copy ratio tables.

Calling absolute copy number is implemented separately from segmentation. The rescale command, an optional step, can adjust a tumor sample’s log_2_ ratios given an estimate of normal-cell contamination (separately derived from cell count or DNA content, see S2 Text), and can re-center the log2_2_ ratios by median, mode, or other measures of central tendency. The call command rounds the log_2_ ratios to the nearest integer absolute copy number given the normal ploidy of each chromosome, or directly maps segment log_2_ ratios to absolute copy number states given a set of numeric thresholds.

### Data summarization, reporting and visualization

CNVkit generates several kinds of plots using the software libraries Biopython [35], Reportlab (http://www.reportlab.com/opensource/) and matplotlib (http://matplotlib.org):

- a “heatmap” of segmented results from multiple samples;
- a single-sample “scatter” plot of bin-level coverages with overlaid segments, either genome-wide or in selected chromosomal regions, optionally with single-nucleotide variant allele frequencies from a separately called Variant Call Format (VCF) file shown to indicate regions of loss of heterozygosity;
- a “diagram” of each chromosome drawn with bin-level copy ratios, segments, or both, labeled with the genes covered by copy number variants.

Copy number features can also be summarized as tabular text reports: Gene-level copy number information can be extracted with the gainloss command, and segmentation breakpoints that fall within a gene (possibly indicating translocation) with the breaks command. Statistics on the residual deviations of bin-level copy ratios from the segmentation calls are calculated per-sample with the metrics command, and per-segment with segmetrics.

### Integration and compatibility with other software

To ease integration into a variety of workflows and pipelines, CNVkit can convert between its native, BED-like file format and formats supported by other software. In particular, the standard SEG format used by GenePattern [36] and Integrative Genomics Viewer [37], and others is supported for both import and export, while standard BED, VCF and Clustered Data Table (CDT) and the native formats of Java TreeView [38] and Nexus Copy Number (BioDiscovery Inc.) are only exported. The per-target coverages reported by the CalculateHsMetrics script in Picard tools (http://picard.sourceforge.net/) can be imported as an alternative to CNVkit’s coverage command. Import and export compatibility with the tumor heterogeneity analysis program THetA2 [39] is implemented to allow fully automated estimation of tumor cell fraction and subclones.

Application wrappers are available for Galaxy [40], DNAnexus, and Docker. CNVkit is also included in the best-practices sequencing analysis pipeline bcbio-nextgen (https://bcbio-nextgen.readthedocs.org/en/latest/) and can be used with the ensemble structural-variant caller MetaSV [41].

## Results

We evaluated our method on DNA sequencing data from targeted sequencing of the melanoma cell line C0902 [42] and two sets of samples, referred to here as “TR” and “EX”, derived from a recent study of advanced melanomas [43]:

- Targeted sequencing (“TR”) of 82 samples, paired tumor and normal tissue from archived microdissected FFPE of 41 melanoma patients, sequenced with a custom 293-gene target capture protocol.
- Exome sequencing (“EX”) of 20 samples, paired fresh frozen tumor and matching blood samples from 10 melanoma patients, sequenced with a whole-exome capture protocol.

Sequencing methods are described in S1 Text.

For each panel of targets, on– and off-target genomic regions are each partitioned into bins (Table 1) in which unique reads are counted in the initial step of copy number estimation. The read counts and percentages in on– and off-target regions for each of these samples are shown in S1 Table.

### Correction of systematic biases in read depth improves copy ratio estimates

**Fig 3:**
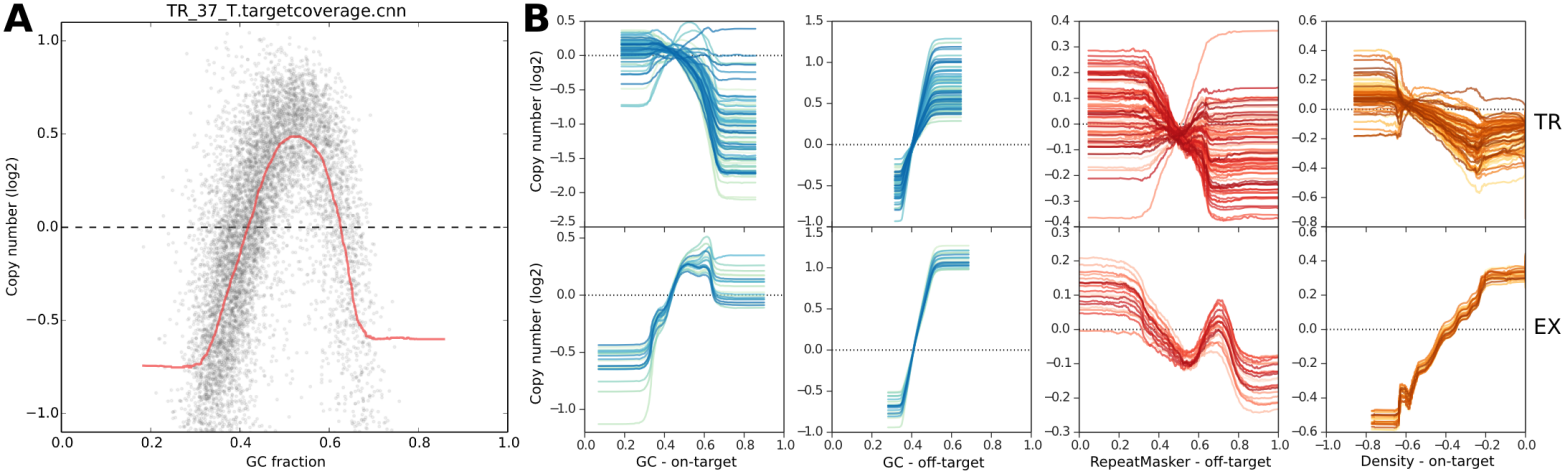
Bin read depths are systematically biased by GC content and other factors. A: GC coverage bias follows a unimodal distribution in sample TR_37_T. Target bins are sorted according to bin GC fraction (x-axis), and the uncorrected, median-centered log_2_ bin read depths are plotted (y-axis). A rolling median of the bin log_2_ read depths in order of GC value is drawn in red, showing a systematic deviation from 0 in the selected sample. B: Trendlines summarize each bias type in each sample. TR and EX samples are shown in the top and bottom rows, respectively. Columns show biases due to GC content in target bins and off-target bins, repeat content in off-target bins, and density bias in target bins.

While normalization to a reference reduces the coverage biases attributable to GC content, repetitive sequence, and target density introduced by library preparation and sequencing, the extent of each of these systematic biases varies from sample to sample (Fig 3), requiring additional correction measures of the residual biases.

**Fig 4:**
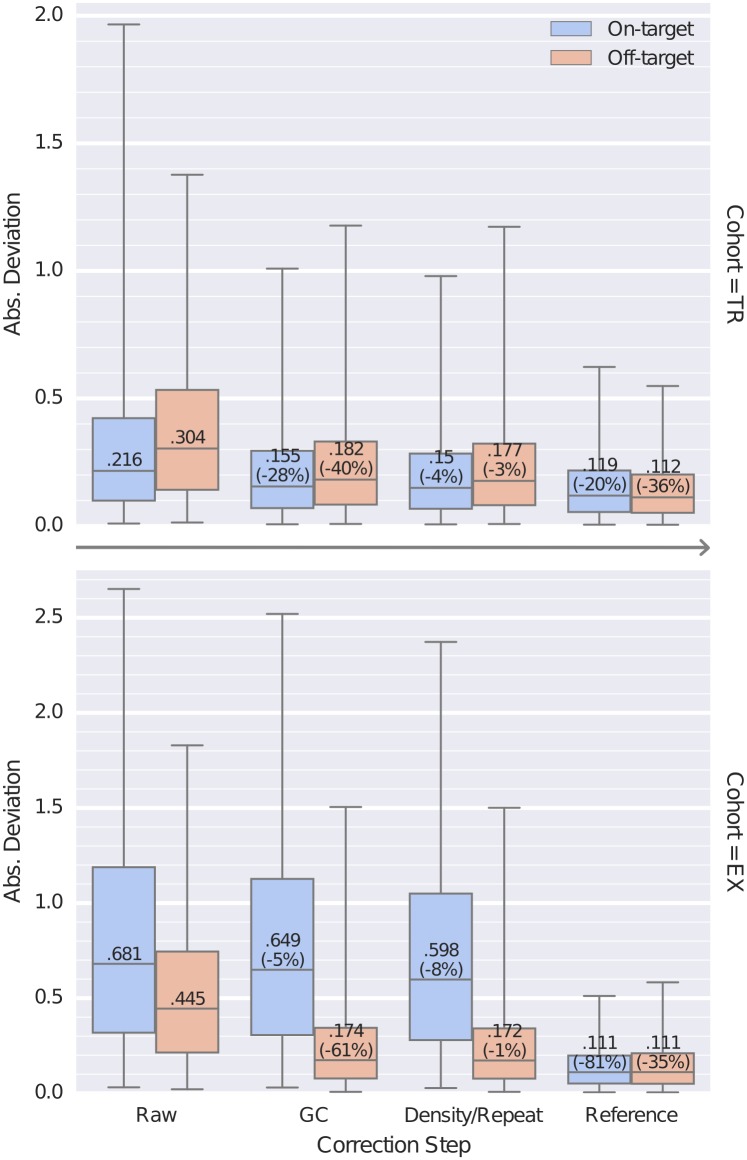
Bias corrections reduce the extraneous variation in bin read depths. Distributions of the absolute deviation of on– and off-target bins from the final, segmented copy ratio estimates are shown as box plots at each step of bias correction for all samples in the TR and EX sequencing cohorts. At each step, for on- and off-target bins separately, boxes show the median and interquartile range of absolute deviations and whiskers show the 95% range. Steps shown are the initial median-centered log_2_ read depth (“Raw”), correction of GC bias (“GC”), correction of on-target density and off-target repeat biases (“Density/Repeat”), and normalization to a pooled reference (“Reference“).

We evaluated the effect of each of our bias corrections by comparing the final segmented copy number data, separately determined with all corrections enabled, to the bin-level read depths or log_2_ ratios for on– and off-target bins at each processing step (Fig 4). For each sample in the TR and EX cohorts, we used CNVkit to perform each of the corrections described above sequentially to estimate bin-level log_2_ ratios. First, we subtracted the uncorrected, median-centered log_2_ read depth of each on– and off-target bin from the corresponding log_2_ copy ratio values of the final segmentation to obtain the deviations of each bin from our final estimate of true log_2_ copy ratio. We repeated this calculation at each of the subsequent steps of bias correction: (i) after GC bias correction; (ii) after the density and repeat corrections; and (iii) after normalizing to a pooled reference.

In these results, the deviation values decreased monotonically across all steps, indicating that each step of corrections reduces random deviations from the true copy number signal/value. The spread of deviation values also decreased overall, indicating that the improvements are seen consistently and are reliable; even outlier data points (representing samples with poor overall sequencing quality) were consistently improved. The greatest improvements were seen from GC bias correction and normalization to the pooled reference.

While each step reduced deviations of off-target bins similarly between the two cohorts, the on-target bins in the EX cohort appear to exhibit more variation in read depths that is independent of GC and targeting density, but consistently removed by refererence normalization. This difference between the two cohorts may be due to differences in the target capture kits’ probe design, the diversity of genes captured, and the type of samples sequenced. In particular, the Nimblegen custom panel used for TR primarily captures average-sized genes that are amenable to hybridization, while the Agilent exome panel used for EX captures nearly all protein-coding genes.

We also found that the deviation of the off-target bins was inversely related to the off-target bin size, or equivalently, directly related of the number of reads captured in each off-target bin. Thus, by choosing the off-target bin size to match the average read counts for on-target bins, we ensured that the deviations or random error in the read counts per bin was similar between on– and off-target bins.

### Validation by array CGH and FISH assays

We validated the segmented copy ratio estimates by CNVkit with respect to two widely used methods for copy number measurement, array CGH (Agilent 4x180K) and fluorescence in situ hybridization (FISH) (see S1 Text). For this validation we used the C0902 cell line, derived from a melanoma.

**Fig 5:**
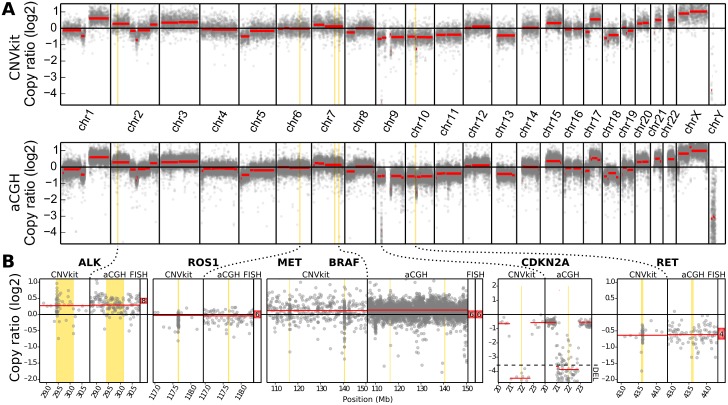
CNVkit copy ratios agree with experimental results array CGH and FISH on cell line DNA. A: Whole-genome profiles of log_2_ copy ratio by CNVkit (top) and array CGH (bottom) are shown. B: Genes additionally assayed by FISH are labeled with the detected absolute copy number. At CDKN2A, log_2_ ratios below the marked level of -3.58 indicate the site is entirely deleted in the majority of cells.

We compared CNVkit and array CGH copy ratios across the whole genome (Fig 5A). Segmentation by CBS yielded 70 segments from the CNVkit bins and 146 segments from the array CGH probes. The median absolute deviation (MAD) of the residual bin– or probe-level log_2_ ratio values from the corresponding array CGH segment means was 0.2002 by array CGH and 0.1531 by CNVkit.

One multi-copy gene-level deletion was detected by array CGH but not by CNVkit. In the gene CEBPA, a 27.5-kilobase loss with a log_2_ copy ratio of -1.5691 is detected by 8 array CGH probes, but the corresponding CNVkit bins showed neutral copy number. These bins cover sequence regions with very high GC content (73–82%) and the CNVkit reference indicated an expected read depth significantly below the genome-wide average, which may have masked any true copy number loss at this locus in the sequencing data. Further comparison of the CNV calls made by array CGH and CNVkit is presented in the next section and in S1 Text.

Next, we used FISH to determine the absolute copy number at loci harboring cancer-relevant genes: ALK, ROS1, MET, BRAF and RET.

We compared the log_2_ ratios obtained by both CNVkit and array CGH to the average signal counts per nucleus obtained by FISH. We transferred the average FISH signal counts into log_2_ copy ratios, by calculating the difference between the log_2_ of their average nuclear signal counts and the log_2_ of the cell’s ploidy, which we determined to be 6n. In all five of the genes assayed by FISH, the copy ratio inferred by CNVkit is close to the average value observed by FISH (Fig 5B).

### Comparison to related software

CNVkit is the first CNV caller to automatically combine copy number information from both on– and off-target regions. These two sources of copy number information have been separately considered in other methods and their software implementations. In particular, CONTRA [9] implemented a pipeline for inferring copy number from targeted regions alone, and CopywriteR [22] recently demonstrated that copy number information can be obtained from off-target reads alone, but neither attempted to combine the off-target and on-target information. Like CNVkit, CONTRA and CopywriteR both use the CBS algorithm to perform segmentation, and both report segment means without requiring an integer copy number value—a feature essential for reporting CNVs in heterogeneous samples. We therefore selected CONTRA and CopywriteR for evaluation alongside CNVkit on the same targeted and whole-exome sequencing datasets presented earlier in this text.

We performed array CGH on each sample in the TR cohort using the Agilent 180K array, and on the EX cohort using the Agilent 1-million-probe array. We used the GenePattern server [36] to segment the array CGH log_2_ ratio values by CBS. The analysis pipelines for CNVkit version 0.7.6, CopywriteR version 1.99.3 and CONTRA version 2.0.6 were run with default settings (see S1 Text). Each of the methods was evaluated using all of the available approaches for constructing a reference: All normal samples pooled (supported by CNVkit and CONTRA), matched tumor-normal pairs (all three methods), and tumor-only calling with no normal reference (CNVkit and CopywriteR). The CNVkit pipeline completed the fastest in all cases, while CopywriteR and CONTRA generally required about 2–4 times as long as CNVkit.

**Fig 6:**
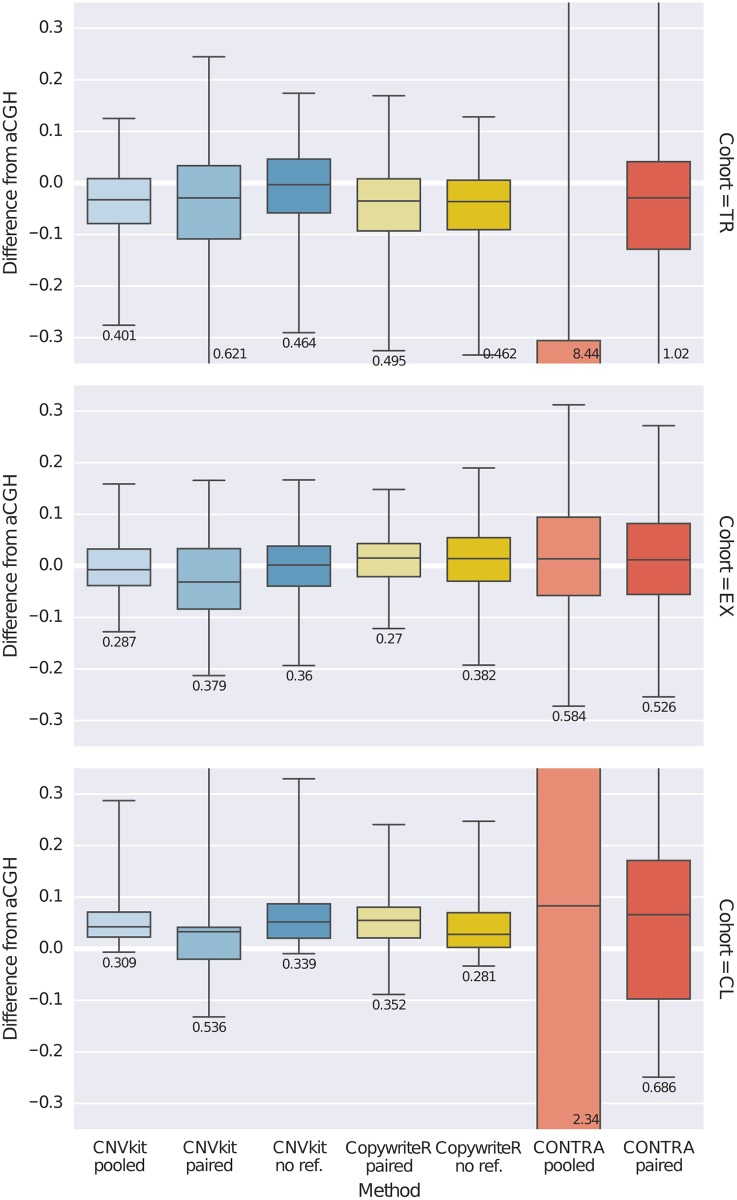
Comparison of CNVkit and other methods to array CGH. Log_2_ ratio estimates by CNVkit, CONTRA and CopywriteR were compared to those by array CGH at each of the targeted genes in the TR and EX cohorts as well as the C0902 cell line sample (CL). The distribution of differences of segmented log_2_ ratio estimates by each caller from that of array CGH at each targeted gene is shown as a box plot, where each box shows the median and interquartile range of absolute deviations, whiskers show the 95% range, and the magnitide of the 95% range (prediction interval) is printed under the box plot. Columns are CNV callers, and rows are the TR and EX cohorts and C0902 sample on which the callers were evaluated.

We compared the CNV calls from each program to those obtained by array CGH. Our primary interest in this evaluation was to see how accurately each method estimates copy ratio at targeted genes, as the inclusion of these genes in a target panel implies that they are the genomic regions of the most interest. We took the differences in segmented log_2_ ratio estimates by array CGH and CNVkit, CONTRA or CopywriteR at each of the targeted genes, and plotted the distributions of these values for comparison (Fig 6). We also calculated the median, 2.5-percentile and 97.5-percentile of each of these distributions to identify the prediction interval (PI) in which 95% of estimates by each method typically deviate from that of array CGH (S1 Table).

With CNVkit, the best estimates were consistently obtained using a pooled reference, then by a “generic” reference, while reference-free calling remained competitive in all cohorts. In the TR cohort CNVkit performed best overall, though when restricted to reference-free calling CNVkit and CopywriteR performed similarly (PI = 0.464 and 0.462, respectively); it is striking to note that in this cohort CopywriteR performed better with no reference than with a single matched normal reference. In the EX cohort CNVkit and CopywriteR achieve reference-free performance (PI = 0.36 and 0.382), and improved by a similar degree using a reference (CNVkit pooled PI = 0.287, CopywriteR paired PI = 0.27). CONTRA did not produce better results than CNVkit or CopywriteR under any conditions, and in the TR cohort and cell line, pooling the reference appeared to exacerbate the inconsistencies apparent in the paired normals.

**Fig 7:**
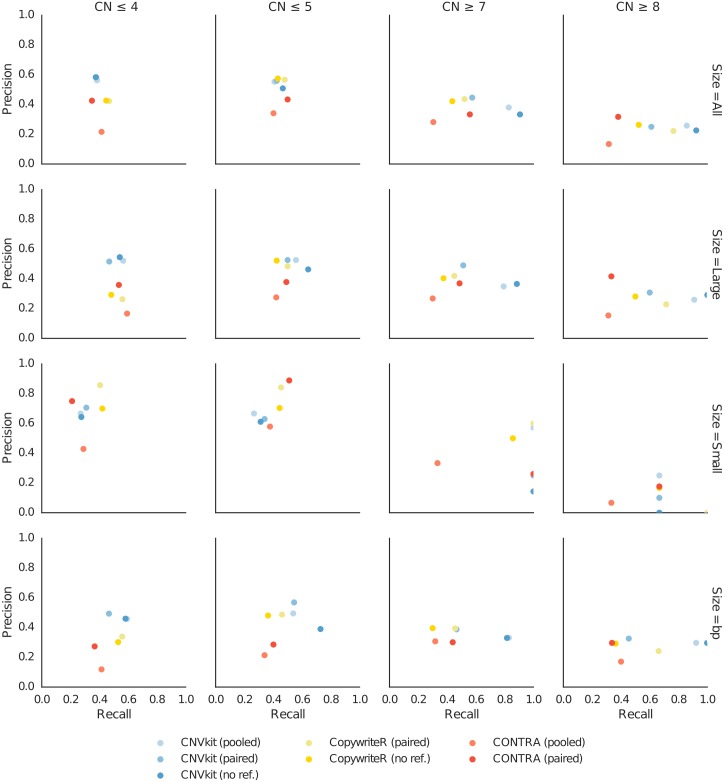
Precion and recall of absolute copy number calls. CNV calls obtained using each sequencing-based method are compared to those determined by array CGH to calculate precision and recall under several criteria for the C0902 cell line sample. Columns show detection of each copy number level versus the neutral hexaploid state. Rows show criteria for comparison: all CNVs, CNVs larger than 5 MB, CNVs smaller than 5MB, all CNV basepairs. Each subplot shows the calculated precision and recall of CNVkit, CopywriteR and CONTRA with each supported reference.

We also investigated genome-wide CNV calling of each method, quantifying performance in terms of precision (specificity) and recall (sensitivity). For the C0902 cell line we derived absolute integer copy numbers from the segmentation obtained by array CGH, CNVkit, CopywriteR, and CONTRA. Treating the array CGH calls as the truth set, we then compared the deletions and duplications from each caller at each copy number state between each caller and array CGH using BEDtools [44], using calls with at least 50% overlap as matches, and calculated the precision and recall for gains and losses of at least one copy and at least two copies (Fig 7). To evaluate performance on larger and smaller CNVs separately, we split the array CGH calls into subsets with CNV sizes above and below 5 megabases, the median size of CNV calls by array CGH, and recalculated precision and recall within each subset. As a check on these results, we also calculated precision and recall across each basepair in all CNVs in lieu of the 50% overlap criterion. As with the gene-level analysis presented above, under most of these metrics CNVkit appears to be competitive with or superior to the other methods.

This evaluation merely considers how well the copy number estimates by several callers agree with array CGH, and ignores key advantages that CNVkit offers—i.e. the ability to efficiently infer copy number from both on– and off-target genomic regions simultaneously, and CNVkit’s extreme flexibility in composing and summarizing the analyses. Nonetheless, CNVkit consistently performs at least as well as, and in some cases much better than, similar software under a range of conditions while maximally extracting copy number resolution from deep sequencing data.

## Availability and Future Directions

CNVkit source code is freely available from https://github.com/etal/cnvkit under the Apache License 2.0 (http://www.apache.org/licenses/LICENSE-2.0). Documentation is available at http://cnvkit.readthedocs.org/ and as S2 Text. Instructions and data files for recreating the analyses presented here are available at http://github.com/etal/cnvkit-examples.

CNVkit provides robust and efficient implementations of methods to improve estimates of copy number from high-throughput sequencing data, making use of both on– and off-target reads from hybrid captures. The flexible design also allows CNVkit to be readily adapted to different sequencing platforms such as Ion Torrent systems (Thermo Fisher Scientific Inc.), and to integrate well into existing analysis pipelines.

The software library underlying CNVkit serves as a basis for developing and benchmarking a variety of approaches to call, analyze and visualize copy number, not unlike the GenomicRanges framework in Bioconductor [45]. The library’s modular design accommodates multiple methods for copy ratio normalization, bias correction and segmentation, and can easily incorporate new methods at any point in the workflow. In particular, we are exploring additional normalization and segmentation approaches within CNVkit to better support whole-genome sequencing and targeted amplicon capture, in which off-target reads are not available to improve copy number estimates. Another current avenue of development is using single-nucleotide polymorphism allele frequencies to assign allele-specific copy number, detect copy-number-neutral loss of heterozygosity, and investigate the structure of tumor heterogeneity in terms of absolute copy number and ploidy in each subclonal cell population.

## Supporting Information

S1 Table Sequencing metrics. Read counts, average coverage and hybridization capture metrics obtained for each sample in the TR and EX cohorts using Picard CalculateHsMetrics.
(TSV)

S1 Text Supporting methods. Descriptions of the experimental procedures and materials used in this study.
(PDF)

S2 Text Documentation. An up-to-date copy is maintained online at http://cnvkit.readthedocs.org/.
(PDF)
