# Supplementary material for: CNVkit: Genome-Wide Copy Number Detection and Visualization from Targeted DNA Sequencing
Source: PLoS Comput Biol. 2016 Apr 21;12(4):e1004873. doi: 10.1371/journal.pcbi.1004873 (PMC4839673; doi:10.1371/journal.pcbi.1004873)
Supplement: S1 Text — Descriptions of the experimental procedures and materials used in this study. (PDF) [file pcbi.1004873.s002.pdf]

Supporting Methods

Eric Talevich, A. Hunter Shain, Thomas Botton, Boris C. Bastian

Accompanying the manuscript "CNVkit: Genome-wide copy number detection and visualization from targeted DNA sequencing"

1 Sequencing

The targeted sequencing cohort (TR) samples were obtained from the archives of the Dermatology Section of the Departments of Pathology and Dermatology at the University of California San Francisco. The exome sequencing cohort (EX) was acquired from Memorial Sloan Kettering Cancer Center. The study was approved by the Institutional Review Boards of both institutions.

Following the manufacturers' protocols, DNA was extracted using Qiagen DNeasy Blood & Tissue Kit and libraries were prepared for sequencing using the NuGen Ovation Ultralow DR Multiplex System 9-16 (p/n 0331-32). Hybrid capture of the whole exome (EX) and of a targeted panel of 293 cancer-associated genes (TR) was performed using the Agilent SureSelectXT Human All Exon V4+UTRs library (p/n 5190-4638) and the Roche Nimblegen SeqCap EZ Choice Library (p/n 06266339001), respectively. DNA library preparation on the C0902 cell line followed a protocol similar to the samples in cohort TR, and a custom target panel different from that used in the TR set, but similarly focused to about 380 genes (Table 1, main text). Multiplexed samples were sequenced on an Illumina HiSeq 2500 instrument.

Sequencing reads were aligned to the UCSC reference human genome (hg19; NCBI build 37) with the Burrows-Wheeler Aligner (BWA) version 0.7.5 [1]. PCR duplicates were flagged with Picard MarkDuplicates, and indel realignment and base quality recalibration were performed with the Genome Analysis Toolkit (GATK) to produce the BAM files used as input to CNVkit.

CNVkit analysis was performed with default settings and bin sizes shown in Table 1, main text. For the TR and EX cohorts, a pooled reference was constructed from the paired normal samples, and for C0902, a reference constructed from four unrelated normal tissue samples.

2 Array CGH

DNA was extracted from the C0902 cell line using a Flexigene DNA extraction kit (Qiagene, Germantown, MD, USA) according to manufacturer's protocol. Array CGH was carried out with 1000 ng of genomic DNA on Agilent 4x180K microarrays (Agilent, Santa Clara, CA, USA). The raw microarray images were processed with Agilent Feature Extraction software.

Probe copy ratio values were then converted to the CNVkit format using the `cnvlib` Python library, skipping unassigned contigs and "dummy" probes. Segmentation was performed on the raw array CGH probe-level $\log_2$ ratios using CBS with the same parameters as default in CNVkit.

3 Fluorescence in situ hybridization (FISH)

We used FISH to determine the absolute copy number at loci harboring the genes ALK, ROS1, MET, BRAF and RET. ROS1 and RET break-apart FISH probes were labeled commercial probes purchased from Kreatech Diagnostics (Amsterdam, The Netherlands). ALK probes were from Abbott Molecular (Des Plaines, Illinois, USA). BRAF, MET and NTRK1 break-apart FISH probes were prepared from BAC clones using standard procedures, and labeled with Fluorolink Cy3-dUTP (GE Healthcare, Waukesha, WI, USA) and ChromaTide Alexa Fluor 488-5-dUTP (Life Technologies, Gaithersburg, MD, USA). After 10 minutes of incubation at 37°C in a hypotonic solution of cell culture medium/distilled water (5:7), C0902 cells were fixed in methanol/glacial acetic acid (3:1) and dropped on a slide. After two days of aging, the slide was treated with RNAse and proteinase K before the probes were hybridized. The number and localization of the hybridization signals was assessed in interphase nuclei with well-delineated contours using a Zeiss fluorescence microscope.

4 Comparison of related software

The analysis pipelines for CNVkit version 0.7.6, CopywriteR version 1.99.3 [2] and CONTRA version 2.0.6 [3] were run on the prepared BAM files from the TR and EX cohorts. Default settings were used for all programs, with the exception that an off-target bin size of 150kb in the TR cohort and 90kb in the EX cohort was used for both CNVkit and CopywriteR. The default off-target bin size suggested by CopywriteR, 20kb, caused the CopywriteR pipeline to reject these samples, presumably because not enough off-target reads were present. In practice we have found that the choice of bin sizes has only a small effect on the segmented results of CNVkit, and this is likely also true for CopywriteR, as both methods use the CBS algorithm similarly for segmentation.

All pipelines were run on a single System76 workstation with 8 Intel CPU cores and 32 GB RAM running Ubuntu Linux 14.04.

Table 1: **Comparison of methods.**

Cohort	Method	Reference	N	Median	2.5%ile	97.5%ile	PI span	2 Std.Dev.	Max.Abs.Dev.
TR	CNVkit	pooled	9918	-0.03252	-0.27600	0.12517	**0.40118**	**0.21685**	**1.17580**
TR	CNVkit	paired	9918	-0.02894	-0.37619	0.24498	0.62116	0.33372	*1.54413*
TR	CNVkit	none	9918	-0.00332	-0.29019	0.17410	0.46428	0.44247	2.74195
TR	CopywriteR	paired	9863	-0.03498	-0.32522	0.16962	0.49484	*0.25616*	3.30901
TR	CopywriteR	none	9880	-0.03600	-0.33358	0.12832	*0.46190*	0.98555	5.76165
TR	CONTRA	pooled	9918	-0.43972	-1.67558	6.76739	8.44296	3.84555	8.33756
TR	CONTRA	paired	9918	-0.02880	-0.60003	0.42301	1.02304	0.47709	3.63433
EX	CNVkit	pooled	178578	-0.00742	-0.12779	0.15884	*0.28663*	*0.18249*	7.21099
EX	CNVkit	paired	178579	-0.03126	-0.21279	0.16581	0.37860	0.23635	**4.83219**
EX	CNVkit	none	178579	0.00147	-0.19327	0.16671	0.35997	0.49359	6.89531
EX	CopywriteR	paired	177757	0.01506	-0.12159	0.14821	**0.26980**	**0.16101**	6.06287
EX	CopywriteR	none	177096	0.01404	-0.19253	0.18978	0.38231	1.00843	6.35367
EX	CONTRA	pooled	178579	0.01361	-0.27209	0.31228	0.58436	0.34715	7.14429
EX	CONTRA	paired	178579	0.01161	-0.25400	0.27195	0.52595	0.26958	*4.99289*
CL	CNVkit	pooled	384	0.04249	-0.00730	0.30125	*0.30854*	0.35037	1.95162
CL	CNVkit	paired	384	0.03290	-0.13220	0.40348	0.53568	*0.24026*	**1.37841**
CL	CNVkit	none	384	0.05186	-0.00958	0.32955	0.33912	0.31311	1.61188
CL	CopywriteR	paired	380	0.05497	-0.08877	0.26308	0.35185	**0.21209**	*1.43783*
CL	CopywriteR	none	380	0.02781	-0.03407	0.24725	**0.28132**	0.27466	1.70973
CL	CONTRA	pooled	384	0.08318	-1.10353	1.23905	2.34258	1.35676	2.77963
CL	CONTRA	paired	384	0.06609	-0.24911	0.43651	0.68562	0.43487	1.60710

Segmented $\log_2$ ratio estimates by CNVkit, CopywriteR and CONTRA were compared to those by array CGH at each of the targeted genes in the TR and EX cohorts. The best estimate in each cohort is shown in bold text, and the second-best in italics.

5 Additional analyses

On– and off-target read depths similarly reflect copy number

Figure 1: **Uncorrected read depths in on– and off-target intervals show a moderate correlation.** A: A putative amplicon including the targeted gene MITF shows similarly increased read depth in both the targeted exons and the adjacent off-target regions. Top row: Coverage depth in and near the MITF region in a melanoma sample, visualized in logarithmic scale in the Integrative Genomics Viewer. Middle: Uncorrected read depths in on– and off-target bins, $\log_2$-transformed and median-centered separately. Bottom: RefGene exonic structure of the MITF gene, and baited intervals used for target enrichment. B: Correlation of mean on– and off-target bin coverages within selected genes across all tumor samples in the TR and EX sets. Density of data points along each axis is shown as a histogram at the top and right edge of each plot, and as color saturation in the EX plot.

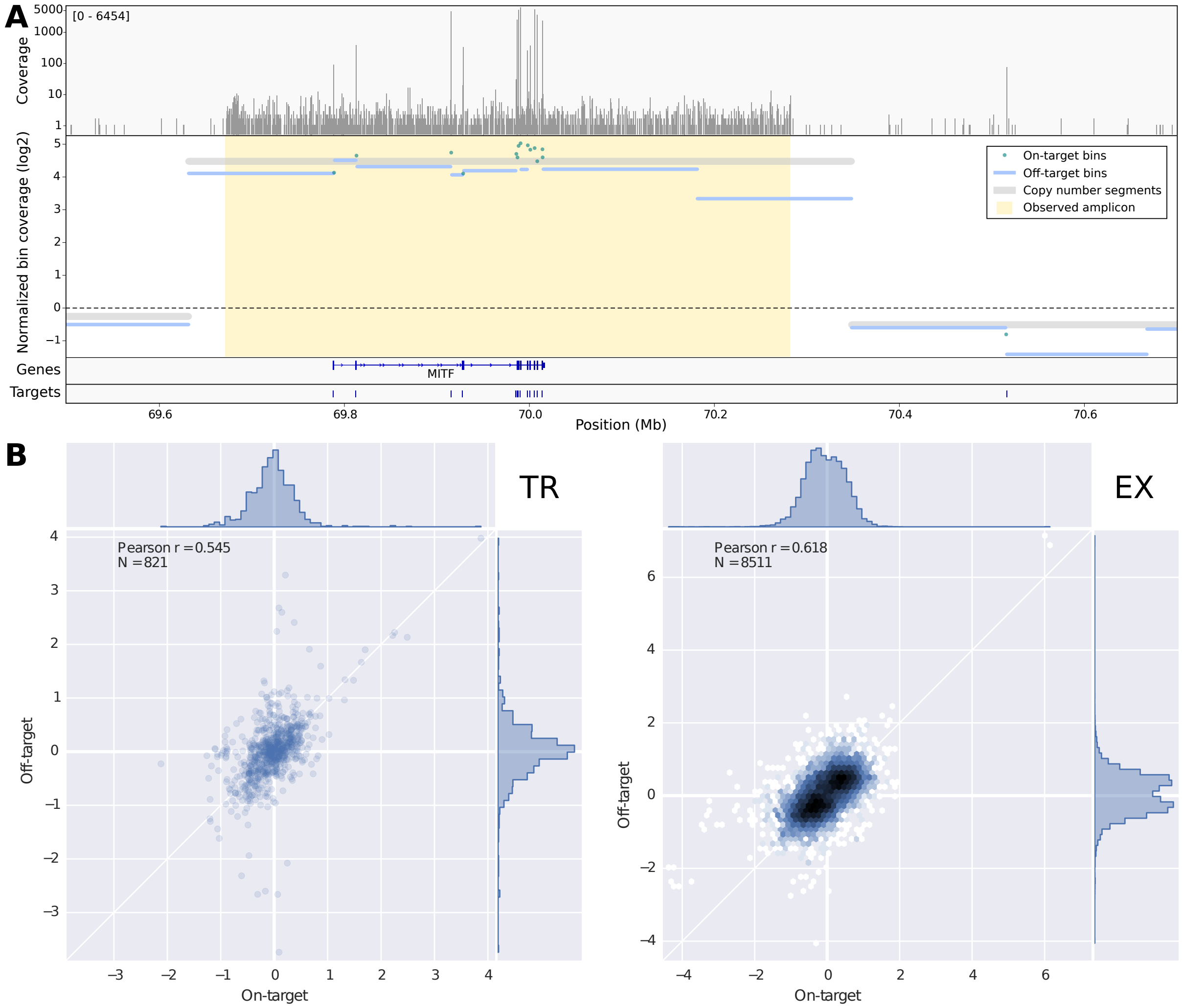

Empirically, we have observed extreme amplifications in off-target areas as sharply demarcated regions with increased read depth (Figure 1A). Since read depth and copy number have been previously shown to be closely correlated [4–7], we therefore hypothesized a proportional relationship between read depth and copy number in on– and off-target bins. The overall agreement in on– and off-target bin read depths can be visually verified within selected regions of an individual sample

by plotting both values together (Figure 1A).

We quantified the level of agreement between on– and off-target read depths more objectively using the TR and EX cohorts. In each cohort we identified the genes containing or adjacent to at least three on– and off-target bins each. For each sample, we performed copy number segmentation with CNVkit and identified the subset of genes in which segmentation indicated a copy number change of at least 0.4-fold at any point within the gene. For each of these genes in this subset, we then calculated the mean of the median-centered $\log_2$ read depths of the on– and off-target bins separately for each sample. We compared these values from all qualifying genes of all samples and confirmed that the mean on– and off-target $\log_2$ read depths correlated strongly within genes and appeared to be linearly related across a considerable range (Figure 1B). Thus, off-target read depth provides similar information on copy number status as the on-target read depth.

A substantial amount of noise remains in the relationship between on– and off-target read depths, however. To reduce systematic noise from the copy number signal derived from targeted sequencing data, we next sought to identify and remove extraneous sources of variation in read depth.

Off-target reads reliably map to the genome

In using off-target reads to estimate copy number, we raised the question of whether the discrepencies in read counts between on– and off-target regions are partly due to unreliable mapping of off-target reads; in particular, whether the mapping quality of off-target reads is significantly less than that of on-target reads.

The mapping quality score (MAPQ) assigned to each aligned read by BWA and similar software indicates the reliability of the read's position, taking into account read base qualities as well as the alignment scores of the best alignment and secondary or suboptimal alignments, and assigning lower scores for reads mapped to repetitive sequence regions of the reference genome [8, 9]. The maximum reported quality score (MAPQ = 60) indicates unambiguous mapping of a read.

For each of the sample cohorts described above (TR, EX), we extracted the mapping qualities of reads in target and off-target regions and compared them to address this question. In the TR samples 98.5% of on-target reads but slightly fewer of the off-target reads (88.3%) were mapped with the maximum quality, while in the EX set there was no overall difference in mapping qualities between on– and off-target reads, with 99.0% mapped with maximum quality in both cases (Figure 2).

Figure 2: **Mapping quality scores of on– and off-target reads are comparable.** For each sample, reads are counted and grouped by MAPQ score within each range labeled on the x-axis, then normalized to the total number of reads obtained from the sample. Bar height indicates the mean of these percentages within each bin; error lines indicate 95% confidence intervals. Scores are shown separately for the targeted (TR) and exome (EX) samples.

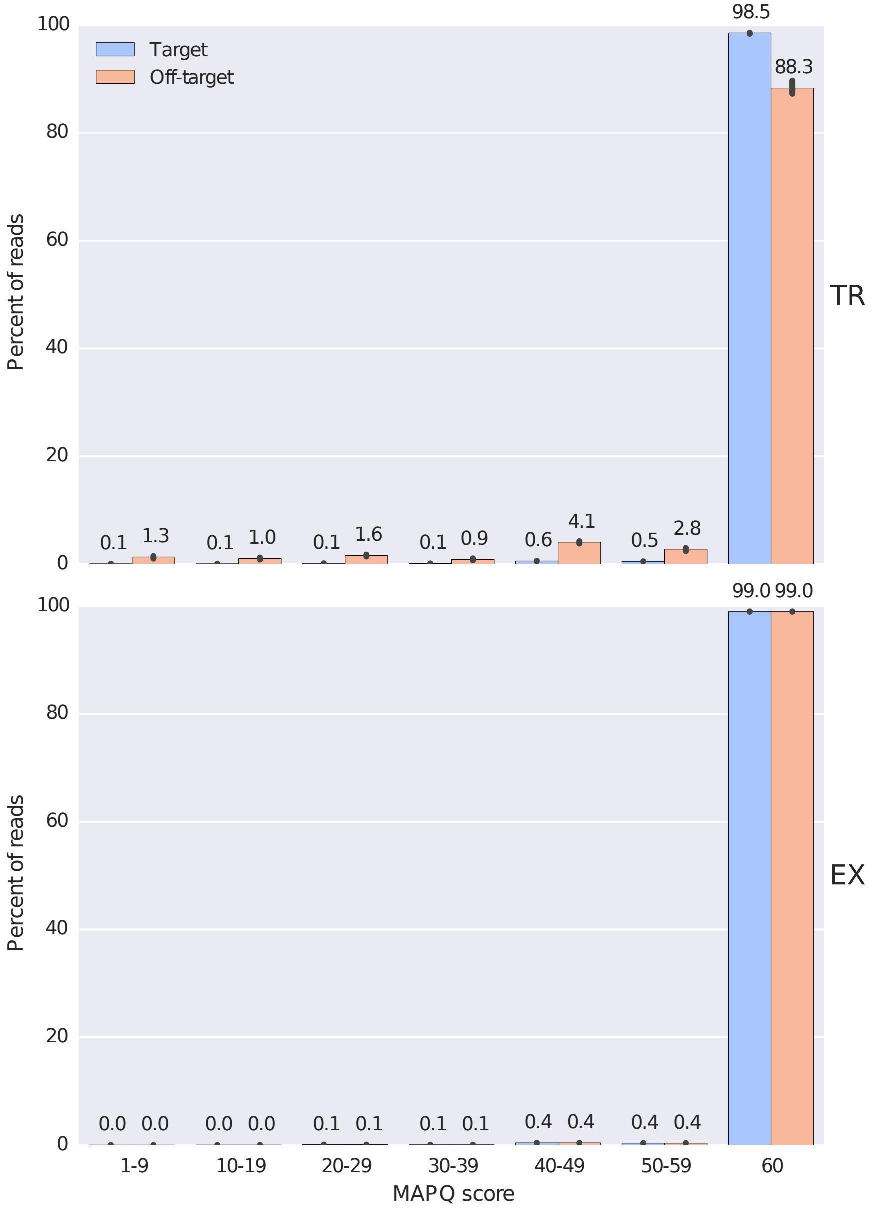

References

[1] Li H. Toward better understanding of artifacts in variant calling from high-coverage samples. Bioinformatics (Oxford, England). 2014 Apr;p. 1–9.

[2] Kuilman T, Velds A, Kemper K, Ranzani M, Bombardelli L, Hoogstraat M, et al. CopywriteR: DNA copy number detection from off-target sequence data. Genome Biology. 2015 Dec;16(1):49.

[3] Li J, Lupat R, Amarasinghe KC, Thompson ER, Doyle Ma, Ryland GL, et al. CONTRA: copy number analysis for targeted resequencing. Bioinformatics. 2012 May;28(10):1307–13.

[4] Alkan C, Kidd JM, Marques-Bonet T, Aksay G, Antonacci F, Hormozdiari F, et al. Personalized copy number and segmental duplication maps using next-generation sequencing. Nature Genetics. 2009 Oct;41(10):1061–7.

[5] Chiang DY, Getz G, Jaffe DB, O'Kelly MJT, Zhao X, Carter SL, et al. High-resolution mapping of copy-number alterations with massively parallel sequencing. Nature Methods. 2009 Jan;6(1):99–103.

[6] Xie C, Tammi MT. CNV-seq, a new method to detect copy number variation using high-throughput sequencing. BMC Bioinformatics. 2009 Jan;10:80.

[7] Yoon S, Xuan Z, Makarov V, Ye K, Sebat J. Sensitive and accurate detection of copy number variants using read depth of coverage. Genome Research. 2009 Sep;19(9):1586–92.

[8] Li H, Ruan J, Durbin R. Mapping short DNA sequencing reads and calling variants using mapping quality scores. Genome Research. 2008 Nov;18(11):1851–8.

[9] Li H, Durbin R. Fast and accurate long-read alignment with Burrows-Wheeler transform. Bioinformatics. 2010 Mar;26(5):589–95.
