# Supplementary material for: CNVkit: Genome-Wide Copy Number Detection and Visualization from Targeted DNA Sequencing
Source: PLoS Comput Biol. 2016 Apr 21;12(4):e1004873. doi: 10.1371/journal.pcbi.1004873 (PMC4839673; doi:10.1371/journal.pcbi.1004873)
Supplement: S2 Text — An up-to-date copy is maintained online at http://cnvkit.readthedocs.org/. (PDF) [file pcbi.1004873.s003.pdf]

CNVkit Documentation

Release 0.7.6

Eric Talevich

February 3, 2016

1 Quick start **3**
1.1 Install CNVkit . . . 3
1.2 Download the reference genome . . . 3
1.3 Map sequencing reads to the reference genome . . . 4
1.4 Build a reference from normal samples and infer tumor copy ratios . . . 4
1.5 Process more tumor samples . . . 5

2 Command line usage **7**
2.1 Copy number calling pipeline . . . 8
2.2 Plots and graphics . . . 16
2.3 Text and tabular reports . . . 22
2.4 Compatibility and other I/O . . . 25
2.5 Additional scripts . . . 27

3 FAQ & How To **29**
3.1 File formats . . . 29
3.2 Bias corrections . . . 31
3.3 Calling absolute copy number . . . 32
3.4 Tumor heterogeneity . . . 33
3.5 Whole-genome sequencing and targeted amplicon capture . . . 35

4 Python API **37**
4.1 Python API (cnvlib package) . . . 37

5 Citation **57**
5.1 Who is using CNVkit? . . . 57

6 Indices and tables **59**

Python Module Index **61**

Index **63**

Author Eric Talevich

Contact eric.talevich@ucsf.edu

License Apache License 2.0

Source code GitHub

Packages PyPI | Docker | Galaxy | DNAnexus

Q&A Biostars | SeqAnswers

CNVkit is a Python library and command-line software toolkit to infer and visualize copy number from targeted DNA sequencing data. It is designed for use with hybrid capture, including both whole-exome and custom target panels, and short-read sequencing platforms such as Illumina and Ion Torrent.

CHAPTER

ONE

QUICK START

If you would like to quickly try CNVkit without installing it, try our app on DNAnexus.

To run CNVkit on your own machine, keep reading.

1.1 Install CNVkit

Download the source code from GitHub:

https://github.com/etal/cnvkit

And read the README file.

1.2 Download the reference genome

Go to the UCSC Genome Bioinformatics website and download:

1. Your species' reference genome sequence, in FASTA format [required]
2. Gene annotation database, via RefSeq or Ensembl, in "flat" format (e.g. refFlat.txt) [optional]

You probably already have the reference genome sequence. If your species' genome is not available from UCSC, use whatever reference sequence you have. CNVkit only requires that your reference genome sequence be in FASTA format. Both the reference genome sequence and the annotation database must be single, uncompressed files.

Sequencing-accessible regions: If your reference genome is the UCSC human genome hg19, a BED file of the sequencing-accessible regions is included in the CNVkit distribution as `data/access-5kb-mappable.hg19.bed`. If you're not using hg19, consider building the "access" file yourself from your reference genome sequence (say, `mm10.fasta`) using the *access* command:

```
cnvkit.py access mm10.fasta -s 10000 -o access-10kb.mm10.bed
```

We'll use this file in the next step to ensure off-target bins ("antitargets") are allocated only in chromosomal regions that can be mapped.

Gene annotations: The gene annotations file (refFlat.txt) is useful to apply gene names to your baits BED file, if the BED file does not already have short, informative names for each bait interval. This file can be used in the next step.

If your targets look like:

```
chr1        1508981 1509154
chr1        2407978 2408183
chr1        2409866 2410095
```

Then you want refFlat.txt.

Otherwise, if they look like:

```
chr1        1508981 1509154 SSU72
chr1        2407978 2408183 PLCH2
chr1        2409866 2410095 PLCH2
```

Then you don't need refFlat.txt.

1.3 Map sequencing reads to the reference genome

If you haven't done so already, use a sequence mapping/alignment program such as BWA to map your sequencing reads to the reference genome sequence.

You should now have one or BAM files corresponding to individual samples.

1.4 Build a reference from normal samples and infer tumor copy ratios

Here we'll assume the BAM files are a collection of "tumor" and "normal" samples, although germline disease samples can be used equally well in place of tumor samples.

CNVkit uses the bait BED file (provided by the vendor of your capture kit), reference genome sequence, and sequencing-accessible regions along with your BAM files to:

1. Create a pooled reference of per-bin copy number estimates from several normal samples; then
2. Use this reference in processing all tumor samples that were sequenced with the same platform and library prep.

All of these steps are automated with the *batch* command. Assuming normal samples share the suffix "Normal.bam" and tumor samples "Tumor.bam", a complete command could be:

```
cnvkit.py batch *Tumor.bam --normal *Normal.bam \
    --targets my_baits.bed --fasta hg19.fasta \
    --split --access data/access-5kb-mappable.hg19.bed \
    --output-reference my_reference.cnn --output-dir example/
```

See the built-in help message to see what these options do, and for additional options:

```
cnvkit.py batch -h
```

If you have no normal samples to use for the reference, you can create a "flat" reference which assumes equal coverage in all bins by using the `--normal/-n` flag without specifying any additional BAM files:

```
cnvkit.py batch *Tumor.bam -n -t my_baits.bed -f hg19.fasta \
    --split --access data/access-5kb-mappable.hg19.bed \
    --output-reference my_flat_reference.cnn -d example2/
```

In either case, you should run this command with the reference genome sequence FASTA file to extract GC and RepeatMasker information for bias corrections, which enables CNVkit to improve the copy ratio estimates even without a paired normal sample.

If your targets are missing gene names, you can add them here with the `--annotate` argument:

```
cnvkit.py batch *Tumor.bam -n *Normal.bam -t my_baits.bed -f hg19.fasta \
    --annotate refFlat.txt --split --access data/access-5kb-mappable.hg19.bed \
    --output-reference my_flat_reference.cnn -d example3/
```

1.5 Process more tumor samples

You can reuse the reference file you've previously constructed to extract copy number information from additional tumor sample BAM files, without repeating the steps above. Assuming the new tumor samples share the suffix "Tumor.bam" (and let's also spread the workload across all available CPUs with the `-p` option, and generate some figures):

```
cnvkit.py batch *Tumor.bam -r my_reference.cnn -p 0 --scatter --diagram -d example4/
```

The coordinates of the target and antitarget bins, the gene names for the targets, and the GC and RepeatMasker information for bias corrections are automatically extracted from the reference .cnn file you've built.

See the command-line usage pages for additional visualization, reporting and import/export commands in CNVkit.

CHAPTER

TWO

COMMAND LINE USAGE

2.1 Copy number calling pipeline

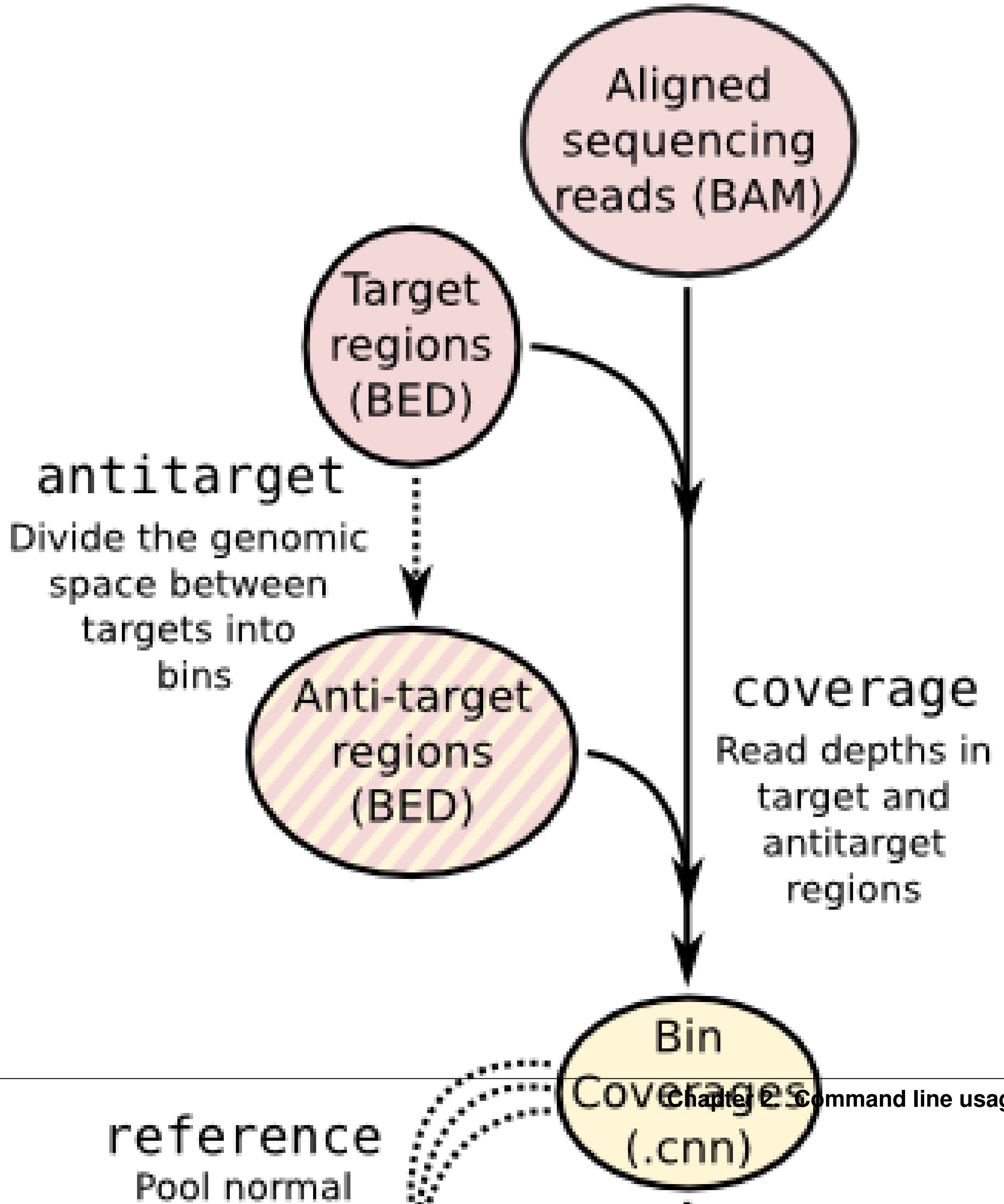

Each operation is invoked as a sub-command of the main script, `cnvkit.py`. A listing of all sub-commands can be obtained with `cnvkit --help` or `-h`, and the usage information for each sub-command can be shown with the `--help` or `-h` option after each sub-command name:

```
cnvkit.py -h
cnvkit.py target -h
```

A sensible output file name is normally chosen if it isn't specified, except in the case of the text reporting commands, which print to standard output by default, and the matplotlib-based plotting commands (not `diagram`), which will display the plots interactively on the screen by default.

2.1.1 batch

Run the CNVkit pipeline on one or more BAM files:

```
# From baits and tumor/normal BAMs
cnvkit.py batch *Tumor.bam --normal *Normal.bam \
    --targets my_baits.bed --split --annotate refFlat.txt \
    --fasta hg19.fasta --access data/access-5kb-mappable.hg19.bed \
    --output-reference my_reference.cnn --output-dir results/ \
    --diagram --scatter

# Reusing a reference for additional samples
cnvkit.py batch *Tumor.bam -r Reference.cnn -d results/

# Reusing targets and antitargets to build a new reference, but no analysis
cnvkit.py batch -n *Normal.bam --output-reference new_reference.cnn \
    -t my_targets.bed -a my_antitargets.bed --male-reference \
    -f hg19.fasta -g data/access-5kb-mappable.hg19.bed
```

With the `-p` option, process each of the BAM files in parallel, as separate subprocesses. The status messages logged to the console will be somewhat disorderly, but the pipeline will take advantage of multiple CPU cores to complete sooner.

```
cnvkit.py batch *.bam -r my_reference.cnn -p 8
```

The pipeline executed by the `batch` command is equivalent to:

```
cnvkit.py target baits.bed [--split --annotate --short-names] -o my_targets.bed
cnvkit.py antitarget my_targets.bed [--access] -o my_antitargets.bed

# For each sample...
cnvkit.py coverage Sample.bam my_targets.bed -o Sample.targetcoverage.cnn
cnvkit.py coverage Sample.bam my_antitargets.bed -o Sample.antitargetcoverage.cnn

# With all normal samples...
cnvkit.py reference *Normal.bam -t my_targets.bed -a my_antitargets.bed \
    [--fasta hg19.fa --male-reference] -o my_reference.cnn

# For each tumor sample...
cnvkit.py fix Sample.targetcoverage.cnn Sample.antitargetcoverage.cnn my_reference.cnn -o Sample.cnr
cnvkit.py segment Sample.cnr -o Sample.cns

# Optionally, with --scatter and --diagram
cnvkit.py scatter Sample.cnr -s Sample.cns -o Sample-scatter.pdf
cnvkit.py diagram Sample.cnr -s Sample.cns [--male-reference] -o Sample-diagram.pdf
```

See the rest of the commands below to learn about each of these steps and other functionality in CNVkit.

2.1.2 target

Prepare a BED file of baited regions for use with CNVkit.

```
cnvkit.py target my_baits.bed --annotate refFlat.txt --split -o my_targets.bed
```

The BED file should be the baited genomic regions for your target capture kit, as provided by your vendor. Since these regions (usually exons) may be of unequal size, the `--split` option divides the larger regions so that the average bin size after dividing is close to the size specified by `--average-size`. If any of these three (`--split`, `--annotate`, or `--short-names`) flags are used, a new target BED file will be created; otherwise, the provided target BED file will be used as-is.

Bin size and resolution

If you need higher resolution, you can select a smaller average size for your target and *antitarget* bins.

Exons in the human genome have an average size of about 200bp. The target bin size default of 267 is chosen so that splitting larger exons will produce bins with a minimum size of 200. Since bins that contain fewer reads result in a noisier copy number signal, this approach ensures the "noisiness" of the bins produced by splitting larger exons will be no worse than average.

Setting the average size of target bins to 100bp, for example, will yield about twice as many target bins, which might result in higher-resolution segmentation. However, the number of reads counted in each bin will be reduced by about half, increasing the variance or "noise" in bin-level coverages. An excess of noisy bins can make visualization difficult, and since the noise may not be Gaussian, especially in the presence of many bins with zero reads, the CBS algorithm could produce less accurate segmentation results on low-coverage samples. In practice we see good results with an average of 200-300 reads per bin; we therefore recommend an overall on-target sequencing coverage depth of at least 200x to 300x with a read length of 100 to justify reducing the average target bin size to 100bp.

Adding gene names

In case the vendor BED file does not label each region with a corresponding gene name, the `--annotate` option can add or replace these labels. Gene annotation databases, e.g. RefSeq or Ensembl, are available in "flat" format from UCSC (e.g. refFlat.txt for hg19).

In other cases the region labels are a combination of human-readable gene names and database accession codes, separated by commas (e.g. "ref|BRAF,mRNA|AB529216,ens|ENST00000496384"). The `--short-names` option splits these accessions on commas, then chooses the single accession that covers in the maximum number of consecutive regions that share that accession, and applies it as the new label for those regions. (You may find it simpler to just apply the refFlat annotations.)

2.1.3 access

Calculate the sequence-accessible coordinates in chromosomes from the given reference genome, output as a BED file.

```
cnvkit.py access hg19.fa -x excludes.bed -o access-hg19.bed
```

Many fully sequenced genomes, including the human genome, contain large regions of DNA that are inaccessable to sequencing. (These are mainly the centromeres, telomeres, and highly repetitive regions.) In the FASTA reference genome sequence these regions are filled in with large stretches of "N" characters. These regions cannot be mapped by resequencing, so we will want to avoid them when calculating the *antitarget* bin locations (for example).

The `access` command computes the locations of the accessible sequence regions for a given reference genome based on these masked-out sequences, treating long spans of 'N' characters as the inaccessible regions and outputting the coordinates of the regions between them.

Other known unmappable or poorly sequenced regions can be specified for exclusion with the `-x` option. This option can be used more than once to exclude several BED files listing different sets of regions. For example, "excludable" regions of poor mappability have been precalculated by others and are available from the UCSC FTP Server (see here for hg19).

If there are many small excluded/inaccessible regions in the genome, then small, less-reliable antitarget bins would be squeezed into the remaining accessible regions. The `-s` option tells the script to ignore short regions that would otherwise be excluded as inaccessible, allowing larger antitarget bins to overlap them.

An "access" file precomputed for the UCSC reference human genome build hg19, with some know low-mappability regions excluded, is included in the CNVkit source distribution under the `data/` directory (`data/access-5kb-mappable.hg19.bed`).

2.1.4 antitarget

Given a "target" BED file that lists the chromosomal coordinates of the tiled regions used for targeted resequencing, derive a BED file off-target/"antitarget"/"background" regions.

```
cnvkit.py antitarget my_targets.bed -g data/access-5kb-mappable.hg19.bed -o my_antitargets.bed
```

Certain genomic regions cannot be mapped by short-read resequencing (see *access*); we can avoid them when calculating the antitarget locations by passing the locations of the accessible sequence regions with the `-g` or `--access` option. CNVkit will then compute "antitarget" bins only within the accessible genomic regions specified in the "access" file.

CNVkit uses a cautious default off-target bin size that, in our experience, will typically include more reads than the average on-target bin. However, we encourage the user to examine the coverage statistics reported by CNVkit and specify a properly calculated off-target bin size for their samples in order to maximize copy number information.

Off-target bin size

An appropriate off-target bin size can be computed as the product of the average target region size and the fold-enrichment of sequencing reads in targeted regions, such that roughly the same number of reads are mapped to on– and off-target bins on average — roughly proportional to the level of on-target enrichment.

The preliminary coverage information can be obtained with the script CalculateHsMetrics in the Picard suite (http://picard.sourceforge.net/), or from the console output of the CNVkit *coverage* command when run on the target regions.

2.1.5 coverage

Calculate coverage in the given regions from BAM read depths.

With the -p option, calculates mean read depth from a pileup; otherwise, counts the number of read start positions in the interval and normalizes to the interval size.

```
cnvkit.py coverage Sample.bam Tiled.bed -o Sample.targetcoverage.cnn
cnvkit.py coverage Sample.bam Background.bed -o Sample.antitargetcoverage.cnn
```

Summary statistics of read counts and their binning are printed to standard error when CNVkit finishes calculating the coverage of each sample (through either the *batch* or *coverage* commands).

BAM file preparation

For best results, use an aligner such as BWA-MEM, with the option to mark secondary mappings of reads, and flag PCR duplicates with a program such as SAMBLASTER, SAMBAMBA, or the MarkDuplicates script in Picard tools, so that CNVkit will skip these reads when calculating read depth.

You will probably want to index the finished BAM file using samtools or SAMBAMBA. But if you haven't done this beforehand, CNVkit will automatically do it for you.

Note: The BAM file must be sorted. CNVkit will check that the first few reads are sorted in positional order, and raise an error if they are not. However, CNVkit might not notice if reads later in the file are unsorted; it will just silently ignore the out-of-order reads and the coverages will be zero after that point. So be safe, and sort your BAM file properly.

Note: If you've prebuilt the BAM index file (.bai), make sure its timestamp is later than the BAM file's. CNVkit will automatically index the BAM file if needed – that is, if the .bai file is missing, *or* if the timestamp of the .bai file is older than that of the corresponding .bam file. This is done in case the BAM file has changed after the index was initially created. (If the index is wrong, CNVkit will not catch this, and coverages will be mysteriously truncated to zero after a certain point.) *However,* if you copy a set of BAM files and their index files (.bai) together over a network, the smaller .bai files will typically finish downloading first, and so their timestamp will be earlier than the corresponding BAM or FASTA file. CNVkit will then consider the index files to be out of date and will attempt to rebuild them. To prevent this, use the Unix command `touch` to update the timestamp on the index files after all files have been downloaded.

2.1.6 reference

Compile a copy-number reference from the given files or directory (containing normal samples). If given a reference genome (-f option), also calculate the GC content of each region.

```
cnvkit.py reference -o Reference.cnn -f ucsc.hg19.fa *targetcoverage.cnn
```

The reference can be constructed from zero, one or multiple control samples. A reference should be constructed specifically for each target capture panel (i.e. set of baits) and, ideally, match the type of sample (e.g. FFPE-extracted or fresh DNA) and library preparation protocol or kit used.

Paired or pooled normals

To analyze a cohort sequenced on a single platform, we recommend combining all normal samples into a pooled reference, even if matched tumor-normal pairs were sequenced – our benchmarking showed that a pooled reference performed slightly better than constructing a separate reference for each matched tumor-normal pair. Furthermore, even matched normals from a cohort sequenced together can exhibit distinctly different copy number biases (see Plagnol et al. 2012 and Backenroth et al. 2014); reusing a pooled reference across the cohort provides some consistency to help diagnose such issues.

Notes on sample selection:

- You can use `cnvkit.py metrics *.cnr -s *.cns` to see if any samples are especially noisy. See the *metrics* command.
- CNVkit will usually call larger CNAs reliably down to about 10x on-target coverage, but there will tend to be more spurious segments, and smaller-scale or subclonal CNAs can be hard to infer below that point. This is well below the minimum coverage thresholds typically used for SNV calling, especially for targeted sequencing of tumor samples that may have significant normal-cell contamination and subclonal tumor-cell populations. So, a

normal sample that passes your other QC checks will probably be OK to use in building a CNVkit reference – assuming it was sequenced on the same platform as the other samples you're calling.

If normal samples are not available, it will sometimes be acceptable to build the reference from a collection of tumor samples. You can use the `scatter` command on the raw `.cnn` coverage files to help choose samples with relatively minimal and non-recurrent CNVs for use in the reference.

With no control samples

Alternatively, you can create a "flat" reference of neutral copy number (i.e. log2 0.0) for each probe from the target and antitarget interval files. This still computes the GC content of each region if the reference genome is given.

```
cnvkit.py reference -o FlatReference.cnn -f ucsc.hg19.fa -t Tiled.bed -a Background.bed
```

Possible uses for a flat reference include:

1. Extract copy number information from one or a small number of tumor samples when no suitable reference or set of normal samples is available. The copy number calls will not be quite as accurate, but large-scale CNVs should still be visible.
2. Create a "dummy" reference to use as input to the `batch` command to process a set of normal samples. Then, create a "real" reference from the resulting `*.targetcoverage.cnn` and `*.antitargetcoverage.cnn` files, and re-run `batch` on a set of tumor samples using this updated reference.
3. Evaluate whether a given paired or pooled reference is suitable for an analysis by repeating the CNVkit analysis with a flat reference and comparing the CNAs found with both the original and flat reference for the same samples.

How it works

CNVkit uses robust methods to extract a usable signal from the reference samples.

At each on– and off-target genomic bin, the read depths in each of the given normal samples are calculated and used to estimate the expected read depth and the reliability of this estimate. Specifically, CNVkit calculates Tukey's biweight location, a weighted average of the normalized log2 coverages in each of the input samples, and biweight midvariance, the spread or statistical dispersion of read depth values using a similar weighting scheme. For background on these statistical methods see Lax (1985) and Randal (2008).

To adjust for the lower statistical reliability of a smaller number of samples for estimating parameters, a "pseudocount" equivalent to one sample of neutral copy number is included in the dataset when calculating these values.

If a FASTA file of the reference genome is given, for each genomic bin the fraction of GC (proportion of "G" and "C" characters among all "A", "T", "G" and "C" characters in the subsequence, ignoring "N" and any other ambiguous characters) and repeat-masked values (proportion of lowercased non-"N" characters in the sequence) are calculated and stored in the output reference .cnn file. For efficiency, the samtools FASTA index file (.fai) is used to locate the binned sequence regions in the FASTA file.

The same read-depth bias corrections used in the *fix* command are performed on each of the normal samples here. The result is a reference copy-number profile that can then be used to correct other individual samples.

Note: As with BAM files, CNVkit will automatically index the FASTA file if the corresponding .fai file is missing or out of date. If you have copied the FASTA file and its index together over a network, you may need to use the `touch` command to update the .fai file's timestamp so that CNVkit will recognize it as up-to-date.

2.1.7 fix

Combine the uncorrected target and antitarget coverage tables (.cnn) and correct for biases in regional coverage and GC content, according to the given reference. Output a table of copy number ratios (.cnr).

```
cnvkit.py fix Sample.targetcoverage.cnn Sample.antitargetcoverage.cnn Reference.cnn -o Sample.cnr
```

How it works

The "observed" on- and off-target read depths are each median-centered and bias-corrected, as when constructing the *reference*. The corresponding "expected" normalized log2 read-depth values from the reference are then subtracted for each set of bins.

CNVkit filters out bins failing certain predefined criteria: those where the reference log2 read depth is below a threshold (default -5), the spread of read depths among all normal samples in the reference is above a threshold (default 1.0), or the RepeatMasker-covered proportion of the bin is above a threshold (default 99%).

A weight is assigned to each remaining bin depending on:

1. The size of the bin;
2. The deviation of the bin's log2 value in the reference from 0;
3. The "spread" of the bin in the reference.

(The latter two only apply if at least one normal/control sample was used to build the reference.)

Finally, the corrected on- and off-target bin-level copy ratios with associated weights are concatenated, sorted, and written to a .cnr file.

2.1.8 segment

Infer discrete copy number segments from the given coverage table:

```
cnvkit.py segment Sample.cnr -o Sample.cns
```

By default this uses the circular binary segmentation algorithm (CBS), which performed best in our benchmarking. But with the `-m` option, the faster HaarSeg (`haar`) or Fused Lasso (`flasso`) algorithms can be used instead.

If you do not have R or the R package dependencies installed, but otherwise do have CNVkit properly installed, then `haar` will work for you. The other two methods use R internally.

Fused Lasso additionally performs significance testing to distinguish CNAs from regions of neutral copy number, whereas CBS and HaarSeg by themselves only identify the supported segmentation breakpoints.

2.1.9 rescale

If there is a known level of normal-cell DNA contamination in the analyzed tumor sample (see the page on tumor heterogeneity), you can opt to rescale the log2 copy ratio estimates in your .cnr or .cns file to remove the impact of this contamination, so the resulting log2 ratio values in the file match what would be observed in a completely pure tumor sample.

The calculation of new log2 values for the sex chromosomes depends on the chromosomal gender of the sample and whether a male reference was used, while for autosomes the specified ploidy (default 2, diploid) is used. For example, with tumor purity of 60% and a male reference, letting CNVkit guess the sample's chromosomal gender:

```
cnvkit.py rescale Sample.cns --purity 0.6 -y -o Sample.rescaled.cns
```

This can be done before or after segmentation, using a .cnr or .cns file; the resulting .cns file should be essentially the same.

The `rescale` command can also optionally re-center the log2 values, though this will typically not be needed since the .cnr files are automatically median-centered by the *fix* command when normalizing to a reference and correcting biases. However, if the analyzed genome is highly aneuploid and contains widespread copy number losses or gains unequally, median centering may place copy-number-neutral regions slightly off-center from the expected log2 value of 0.0. To address such cases, alternative centering approaches can be specified with the `--center` option:

```
cnvkit.py rescale Sample.cns --center mode
```

2.1.10 call

Given segmented log2 ratio estimates (.cns), round the copy ratio estimates to integer values using either:

- A list of threshold log2 values for each copy number state, or
- *Rescaling* for a given known tumor cell fraction and normal ploidy, then simple rounding to the nearest integer copy number.

```
cnvkit.py call Sample.cns -o Sample.call.cns
cnvkit.py call Sample.cns -y -m threshold -t=-1.1,-0.4,0.3,0.7 -o Sample.call.cns
cnvkit.py call Sample.cns -y -m clonal --purity 0.65 -o Sample.call.cns
```

The output is another .cns file, where the values in the log2 column are still log2-transformed and relative to the reference ploidy (by default: diploid autosomes, haploid Y or X/Y depending on reference gender). The segment log2 values are simply rounded to what they would be if the estimated copy number were an integer – e.g. a neutral diploid state is represented as 0.0, and a copy number of 3 on a diploid chromosome is represented as 0.58. The output .cns file is still compatible with the other CNVkit commands that accept .cns files, and can be plotted the same way with the *scatter*, *heatmap* and *diagram* commands.

To get the absolute integer copy number values in a human-readable form, use the command *export* `bed`.

Calling methods

The “clonal” method uses the same calculation as the *rescale* command. It considers the observed log2 ratios in the input .cns file as a mix of some fraction of tumor cells (specified by `--purity`), possibly with altered copy number, and a remainder of normal cells with neutral copy number (specified by `--ploidy` for autosomes). This equation is rearranged to find the absolute copy number of the tumor cells alone, rounded to the nearest integer. The expected and observed ploidy of the sex chromosomes (X and Y) is different, so it’s important to specify `-y`/`--male-reference` if a male reference was used; the sample gender can be specified if known, otherwise it will be guessed from the average log2 ratio of chromosome X.

The “threshold” method simply applies fixed log2 ratio cutoff values for each integer copy number state. This method therefore does not require the tumor cell fraction or purity to be known. The default cutoffs are reasonable for a tumor sample with purity of at least 40% or so. For germline samples, the `-t` values shown above may yield more accurate calls.

The thresholds work like:

If $\log_2$ value $\leq$	Copy number
-1.1	0
-0.4	1
0.3	2
0.7	3
...	...

For homogeneous samples of known ploidy, you can calculate cutoffs from scatch by log-transforming the integer copy number values of interest, plus .5 (for rounding), divided by the ploidy. For a diploid genome:

```
>>> import numpy as np
>>> copy_nums = np.arange(5)
>>> print(np.log2((copy_nums+.5) / 2)
[-2.         -0.4150375   0.32192809  0.80735492  1.169925  ]
```

Or, in R:

```
> log2( (0:4 + .5) / 2)
[1] -2.0000000 -0.4150375  0.3219281  0.8073549  1.1699250
```

2.2 Plots and graphics

2.2.1 scatter

Plot bin-level log2 coverages and segmentation calls together. Without any further arguments, this plots the genome-wide copy number in a form familiar to those who have used array CGH.

```
cnvkit.py scatter Sample.cnr -s Sample.cns
# Shell shorthand
cnvkit.py scatter -s TR_95_T.cn{s,r}
```

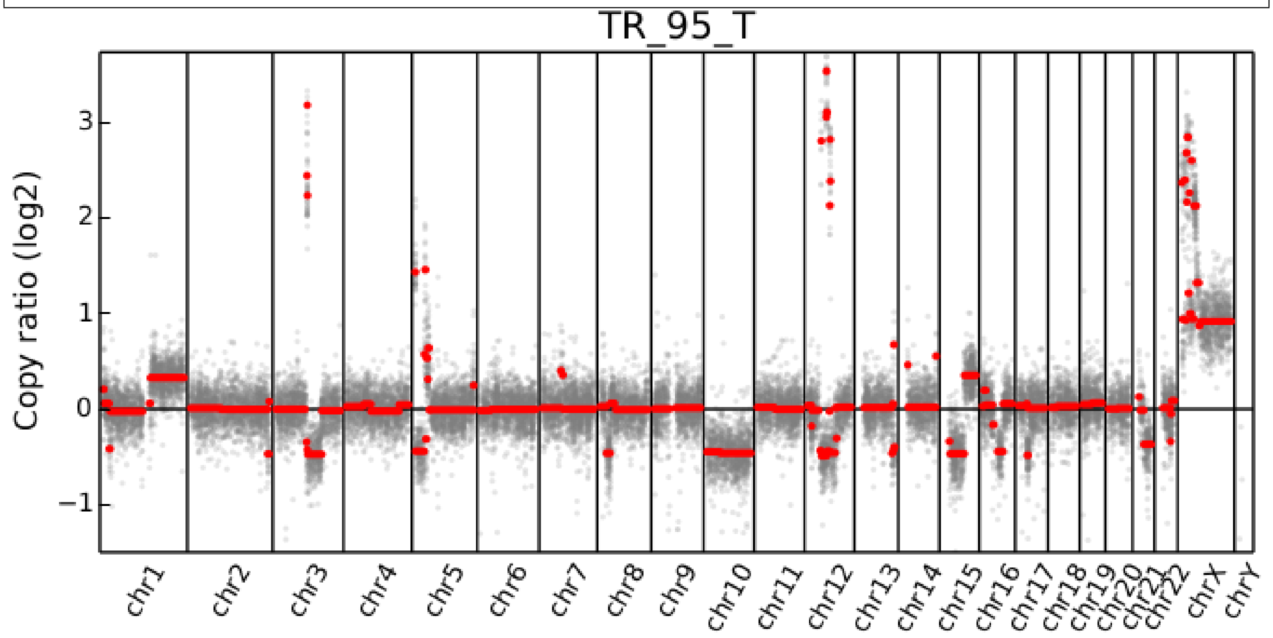

The options `--chromosome` and `--gene` (or their single-letter equivalents) focus the plot on the specified region:

```
cnvkit.py scatter -s Sample.cn{s,r} -c chr7
cnvkit.py scatter -s Sample.cn{s,r} -c chr7:140434347-140624540
cnvkit.py scatter -s Sample.cn{s,r} -g BRAF
```

In the latter two cases, the genes in the specified region or with the specified names will be highlighted and labeled in the plot. The `--width` (`-w`) argument determines the size of the chromosomal regions to show flanking the selected region. Note that only targeted genes can be highlighted and labeled; genes that are not included in the list of targets are not labeled in the .cnn or .cnr files and are therefore invisible to CNVkit.

The arguments `-c` and `-g` can be combined to e.g. highlight specific genes in a larger context:

```
# Show a chromosome arm, highlight one gene
cnvkit.py scatter -s Sample.cn{s,r} -c chr5:100-50000000 -g TERT
# Show the whole chromosome, highlight two genes
cnvkit.py scatter -s Sample.cn{s,r} -c chr7 -g BRAF,MET
# Highlight two genes in a specified range
cnvkit.py scatter -s TR_95_T.cn{s,r} -c chr12:50000000-80000000 -g CDK4,MDM2
```

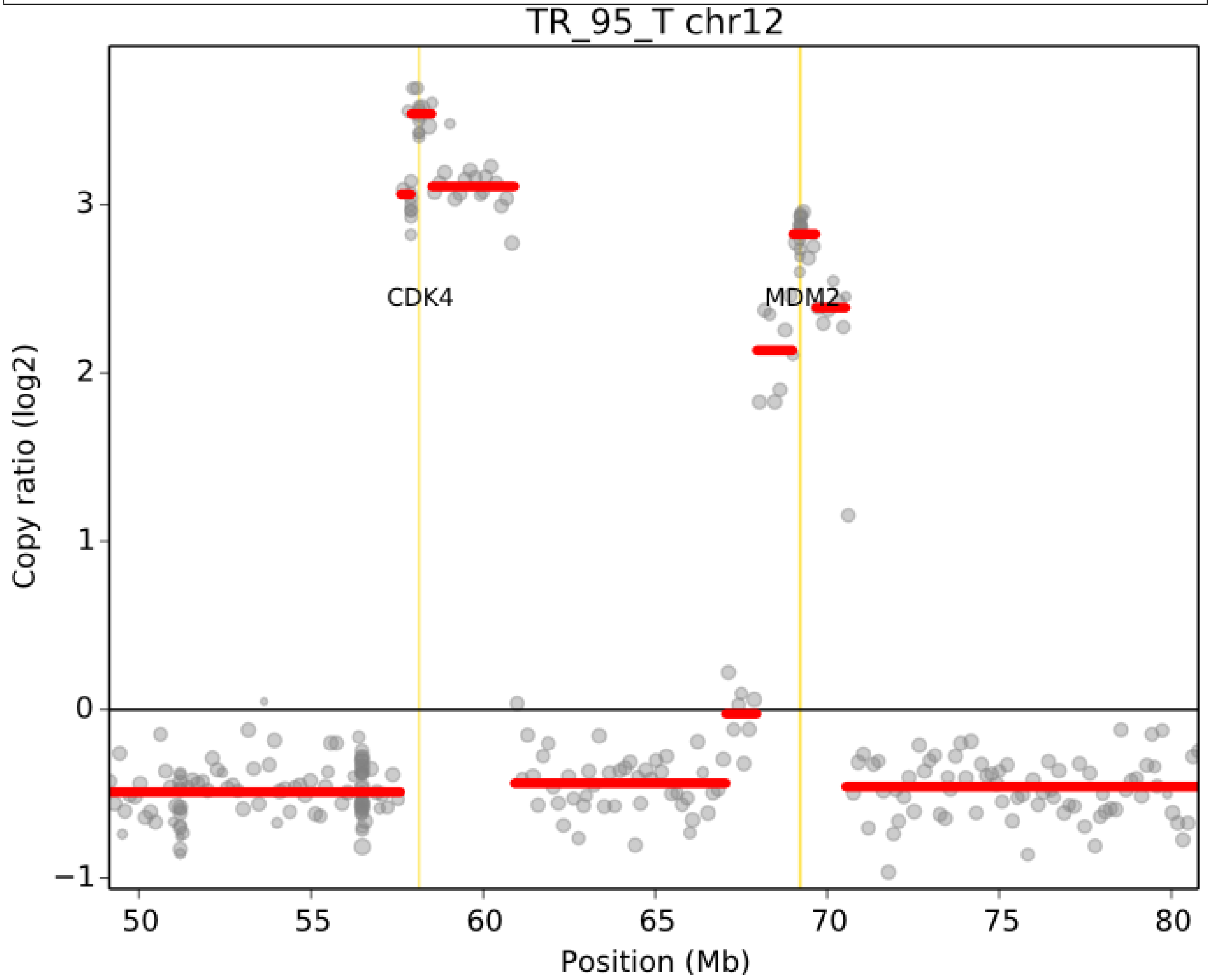

When a chromosomal region is plotted with CNVkit's "scatter" command , the size of the plotted datapoints is proportional to the weight of each point used in segmentation – a relatively small point indicates a less reliable bin. Therefore, if you see a cluster of smaller points in a short segment (or where you think there ought to be a segment, but there isn't one), then you can cast some doubt on the copy number call in that region. The dispersion of points around the segmentation line also visually indicates the level of noise or uncertainty.

To create multiple region-specific plots at once, the regions of interest can be listed in a separate file and passed to the `scattter` command with the `-l`/`--range-list` option. This is equivalent to creating the plots separately with the `-c` option and then combining the plots into a single multi-page PDF.

Loss of heterozygosity (LOH) can be viewed alongside copy number by passing variants as a VCF file with the `-v` option. Heterozygous SNP allelic frequencies are shown in a subplot below the CNV scatter plot. (Also see the *loh* command.)

```
cnvkit.py scatter Sample.cnr -s Sample.cns -v Sample.vcf
```

The bin-level log2 ratios or coverages can also be plotted without segmentation calls:

```
cnvkit.py scatter Sample.cnr
```

This can be useful for viewing the raw, un-corrected coverage depths when deciding which samples to use to build a profile, or simply to see the coverages without being helped/biased by the called segments.

The `--trend` option (`-t`) adds a smoothed trendline to the plot. This is fairly superfluous if a valid segment file is given, but could be helpful if the CBS dependency is not available, or if you're skeptical of the segmentation in a region.

2.2.2 loh

Plot allelic frequencies at each variant position in a VCF file. Given segments, show the mean b-allele frequency values above and below 0.5 of SNVs falling within each segment. Divergence from 0.5 indicates LOH in the tumor sample.

```
cnvkit.py loh Sample.vcf
cnvkit.py loh Sample.vcf -s Sample.cns -i Sample_Tumor -n Sample_Normal
```

Regions with LOH are reflected in heterozygous germline SNPs in the tumor sample with allele frequencies shifted away from the expected 0.5 value. Given a VCF with only the tumor sample called, it is difficult to focus on just the informative SNPs because it's not known which SNVs are present and heterozygous in normal, germline cells. Better results can be had by giving CNVkit more information:

- Call somatic mutations using paired tumor and normal samples. In the VCF, the somatic variants should be flagged in the INFO column with the string "SOMATIC". (MuTect does this automatically.) Then CNVkit will skip these for plotting.
- Add a "PEDIGREE" tag to the VCF header, listing the tumor sample as "Derived" and the normal as "Original". (MuTect doesn't do this, but it does add a nonstandard GATK header that CNVkit can extract the same information from.)
- In lieu of a PEDIGREE tag, tell CNVkit which sample IDs are the tumor and normal using the `-i` and `-n` options, respectively.
- If no paired normal sample is available, you can still filter for likely informative SNPs by intersecting your tumor VCF with a set of known SNPs such as 1000 Genomes, ESP6500, or ExAC. Drop the private SNVs that don't appear in these databases to create a VCF more amenable to LOH detection.

2.2.3 diagram

Draw copy number (either individual bins (.cnn, .cnr) or segments (.cns)) on chromosomes as an ideogram. If both the bin-level log2 ratios and segmentation calls are given, show them side-by-side on each chromosome (segments on the left side, bins on the right side).

```
cnvkit.py diagram Sample.cnr
cnvkit.py diagram -s Sample.cns
cnvkit.py diagram -s Sample.cns Sample.cnr
```

If bin-level log2 ratios are provided (.cnr), genes with log2 ratio values beyond a fixed threshold will be labeled on the plot. This plot style works best with target panels of a few hundred genes at most; with whole-exome sequencing there are often so many genes affected by CNAs that the individual gene labels become difficult to read.

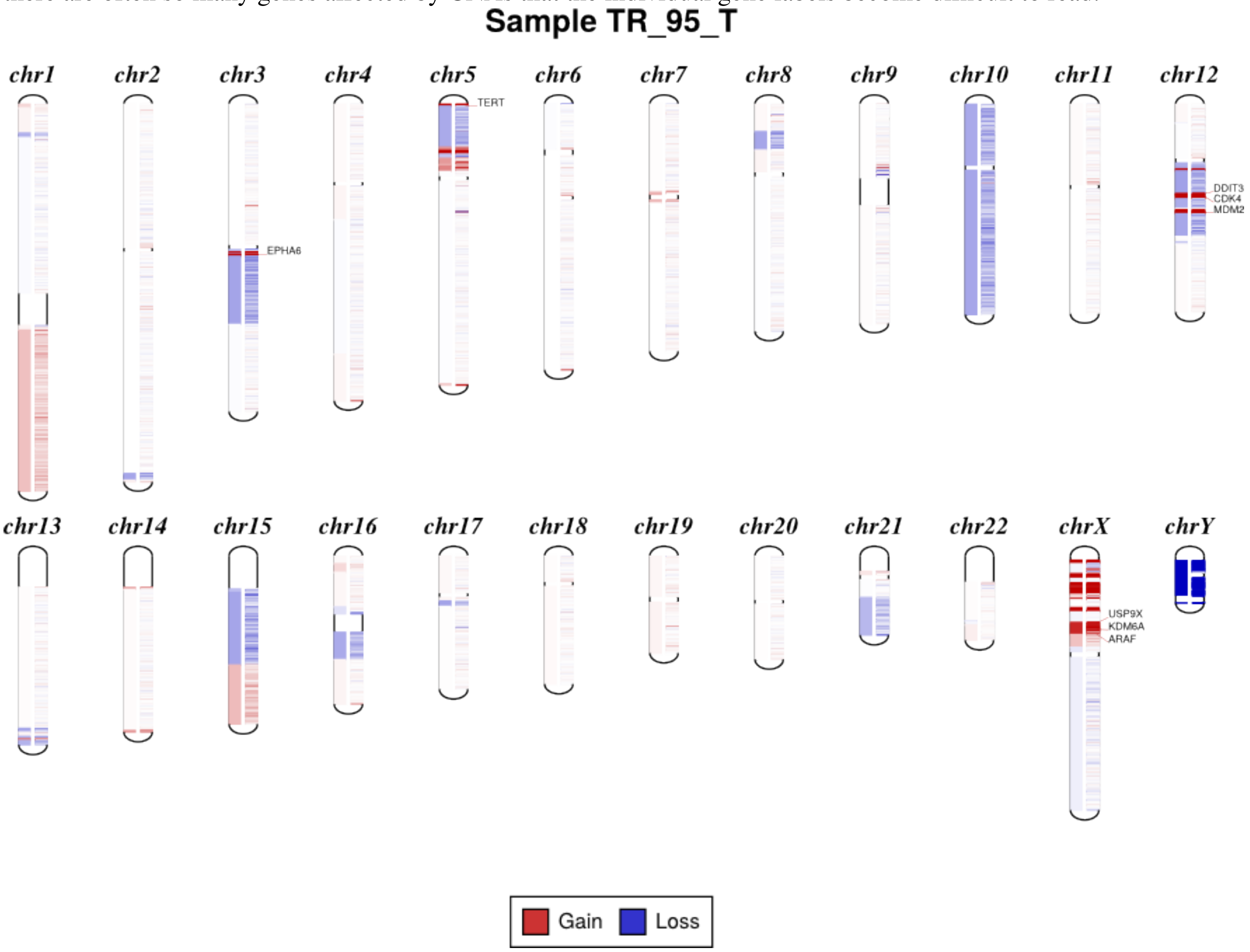

If only segments are provided (-s), gene labels are not shown. This plot is then equivalent to the `heatmap` command, which effectively summarizes the segmented values from many samples.

2.2.4 heatmap

Draw copy number (either bins (.cnn, .cnr) or segments (.cns)) for multiple samples as a heatmap.

To get an overview of the larger-scale CNVs in a cohort, use the "heatmap" command on all .cns files:

```
cnvkit.py heatmap *.cns
```

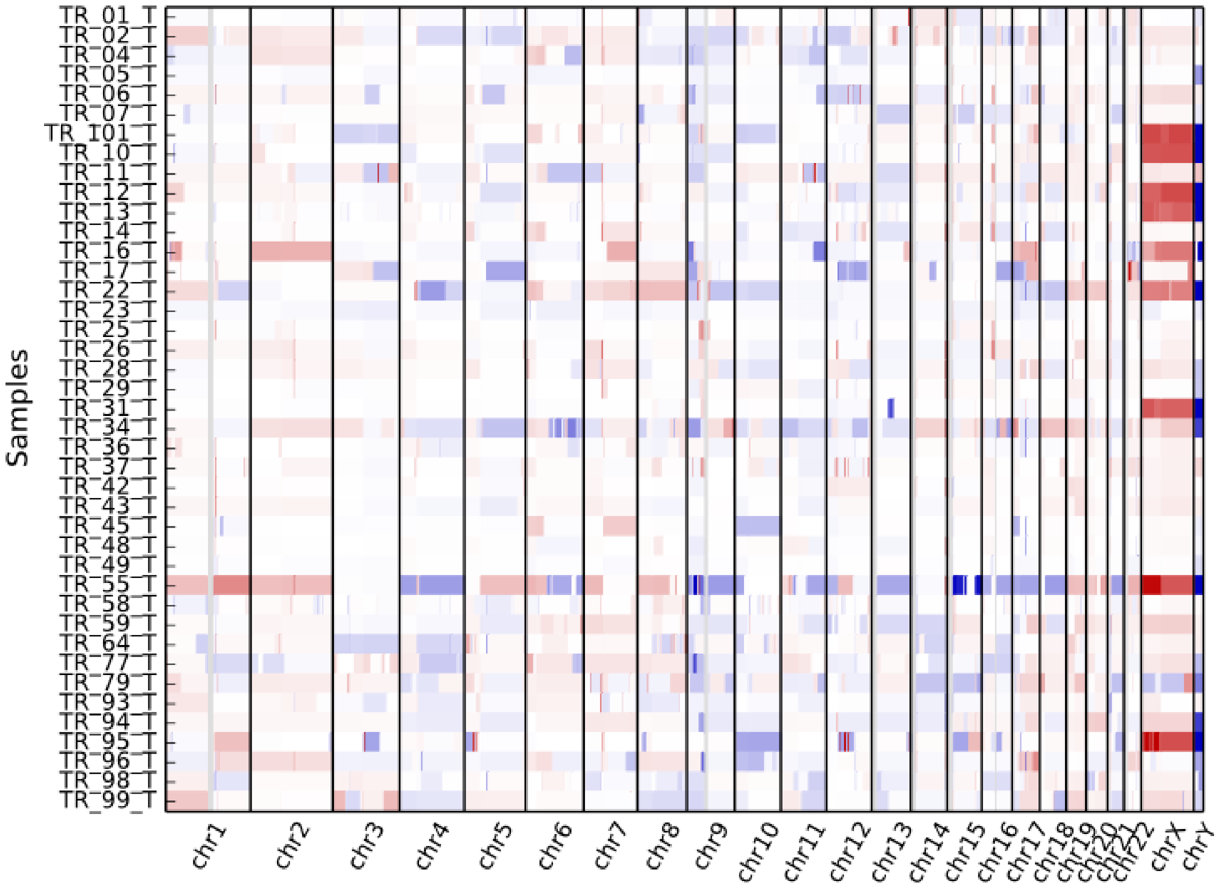

The color range can be subtly rescaled with the `-d` option to de-emphasize low-amplitude segments, which are likely spurious CNAs:

```
cnvkit.py heatmap *.cns -d
```

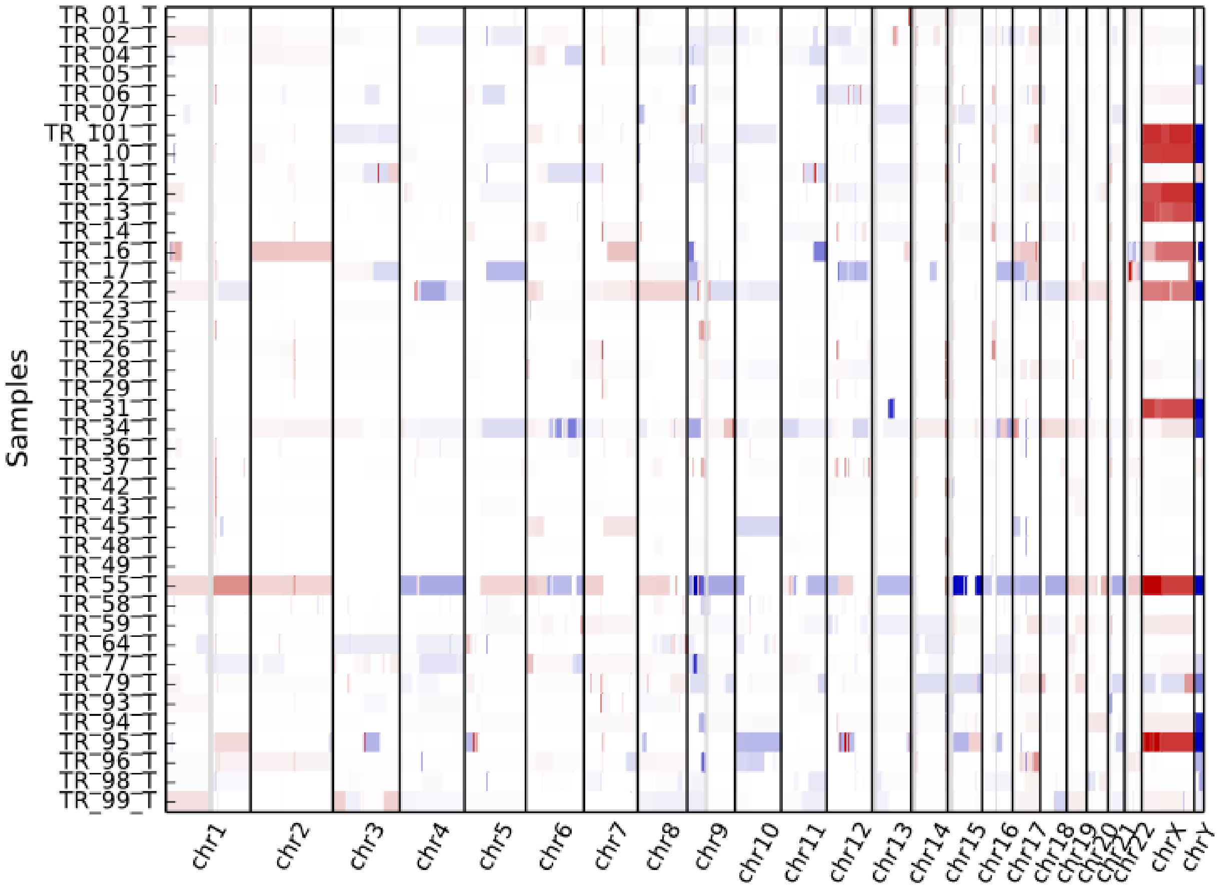

A heatmap can also be drawn from bin-level log2 coverages or copy ratios (.cnn, .cnr), but this will be extremely slow at the genome-wide level. Consider doing this with a smaller number of samples and only for one chromosome or chromosomal region at a time, using the `-c` option:

```
cnvkit.py heatmap TR_9*T.cnr -c chr12  # Slow!
cnvkit.py heatmap TR_9*T.cnr -c chr7:125000000-145000000
```

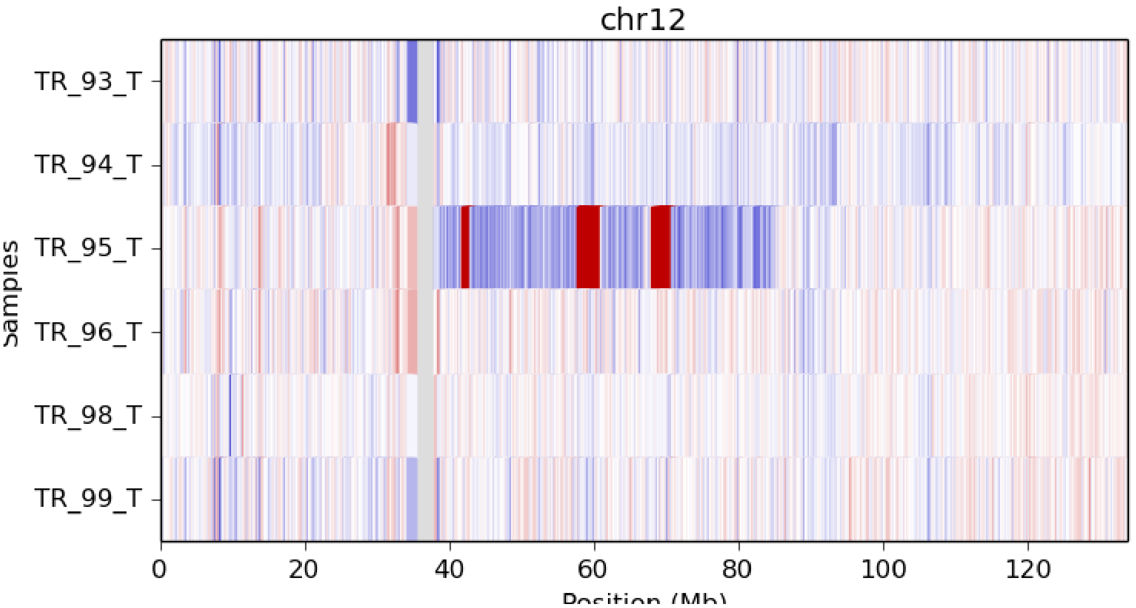

If an output file name is not specified with the `-o` option, an interactive matplotlib window will open, allowing you to select smaller regions, zoom in, and save the image as a PDF or PNG file.

2.3 Text and tabular reports

2.3.1 breaks

List the targeted genes in which a segmentation breakpoint occurs.

```
cnvkit.py breaks Sample.cnr Sample.cns
```

This helps to identify genes in which (a) an unbalanced fusion or other structural rearrangement breakpoint occured, or (b) CNV calling is simply difficult due to an inconsistent copy number signal.

The output is a text table of tab-separated values, which is amenable to further processing by scripts and standard Unix tools such as `grep`, `sort`, `cut` and `awk`.

For example, to get a list of the names of genes that contain a possible copy number breakpoint:

```
cnvkit.py breaks Sample.cnr Sample.cns | cut -f1 | sort -u > gene-breaks.txt
```

2.3.2 gainloss

Identify targeted genes with copy number gain or loss above or below a threshold.

```
cnvkit.py gainloss Sample.cnr
cnvkit.py gainloss Sample.cnr -s Sample.cns -t 0.4 -m 5 -y
```

If segments are given, the log2 ratio value reported for each gene will be the value of the segment covering the gene. Where more than one segment overlaps the gene, i.e. if the gene contains a breakpoint, each segment's value will be reported as a separate row for the same gene. If a large-scale CNA covers multiple genes, each of those genes will be listed individually.

If segments are not given, the median of the log2 ratio values of the bins within each gene will be reported as the gene's overall log2 ratio value. This mode will not attempt to identify breakpoints within genes.

The threshold (`-t`) and minimum number of bins (`-m`) options are used to control which genes are reported. For example, a threshold of .2 (the default) will report single-copy gains and losses in a completely pure tumor sample (or germline CNVs), but a lower threshold would be necessary to call somatic CNAs if significant normal-cell contamination is present. Some likely false positives can be eliminated by dropping CNVs that cover a small number of bins (e.g. with `-m 3`, genes where only 1 or 2 bins show copy number change will not be reported), at the risk of missing some true positives.

Specify the reference gender (`-y` if male) to ensure CNVs on the X and Y chromosomes are reported correctly; otherwise, a large number of spurious gains or losses on the sex chromosomes may be reported.

The output is a text table of tab-separated values, like that of *breaks*. Continuing the Unix example, we can try `gainloss` both with and without the segment files, take the intersection of those as a list of "trusted" genes, and visualize each of them with *scatter*:

```
cnvkit.py gainloss -y Sample.cnr -s Sample.cns | tail -n+2 | cut -f1 | sort > segment-gainloss.txt
cnvkit.py gainloss -y Sample.cnr | tail -n+2 | cut -f1 | sort > ratio-gainloss.txt
comm -12 ratio-gainloss.txt segment-gainloss.txt > trusted-gainloss.txt
for gene in `cat trusted-gainloss.txt`
do
    cnvkit.py scatter -s Sample.cn{s,r} -g $gene -o Sample-$gene-scatter.pdf
done
```

(The point is that it's possible.)

2.3.3 gender

Guess samples' gender from the relative coverage of chromoxsome X. A table of the sample name (derived from the filename), guessed chromosomal gender (string "Female" or "Male"), and log2 ratio value of chromosome X is printed.

```
cnvkit.py gender *.cnn *.cnr *.cns
cnvkit.py gender -y *.cnn *.cnr *.cns
```

If there is any confusion in specifying either the gender of the sample or the construction of the reference copy number profile, you can check what happened using the "gender" command. If the reference and intermediate .cnn files are available (.targetcoverage.cnn and .antitargetcoverage.cnn, which are created before most of CNVkit's corrections), CNVkit can report the reference gender and the apparent chromosome X copy number that appears in the sample:

```
cnvkit.py gender reference.cnn Sample.targetcoverage.cnn Sample.antitargetcoverage.cnn
```

The output looks like this, where columns are filename, apparent gender, and log2 ratio of chrX:

```
cnv_reference.cnn    Female  -0.176
Sample.targetcoverage.cnn    Female  -0.0818
Sample.antitargetcoverage.cnn       Female  -0.265
```

If the `-y` option was not specified when constructing the reference (e.g. `cnvkit.py batch ...`), then you have a female reference, and in the final plots you will see chrX with neutral copy number in female samples and around -1 log2 ratio in male samples.

2.3.4 metrics

Calculate the spread of bin-level copy ratios from the corresponding final segments using several statistics. These statistics help quantify how "noisy" a sample is and help to decide which samples to exclude from an analysis, or to

select normal samples for a reference copy number profile.

For a single sample:

```
cnvkit.py metrics Sample.cnr -s Sample.cns
```

(Note that the order of arguments and options matters here, unlike the other commands: Everything after the `-s` flag is treated as a segment dataset.)

Multiple samples can be processed together to produce a table:

```
cnvkit.py metrics S1.cnr S2.cnr -s S1.cns S2.cns
cnvkit.py metrics *.cnr -s *.cns
```

Several bin-level log2 ratio estimates for a single sample, such as the uncorrected on- and off-target coverages and the final bin-level log2 ratios, can be compared to the same final segmentation (reusing the given segments for each coverage dataset):

```
cnvkit.py metrics Sample.targetcoverage.cnn Sample.antitargetcoverage.cnn Sample.cnr -s Sample.cns
```

In each case, given the bin-level copy ratios (.cnr) and segments (.cns) for a sample, the log2 ratio value of each segment is subtracted from each of the bins it covers, and several estimators of spread are calculated from the residual values. The output table shows for each sample:

- Total number of segments (in the .cns file) – a large number of segments can indicate that the sample has either many real CNAs, or noisy coverage and therefore many spurious segments.
- Uncorrected sample standard deviation – this measure is prone to being inflated by a few outliers, such as may occur in regions of poor coverage or if the targets used with CNVkit analysis did not exactly match the capture. (Also note that the log2 ratio data are not quite normally distributed.) However, if a sample's standard deviation is drastically higher than the other estimates shown by the `metrics` command, that helpfully indicates the sample has some outlier bins.
- Median absolute deviation (MAD) – very robust against outliers, but less statistically efficient.
- Interquartile range (IQR) – another robust measure that is easy to understand.
- Tukey's biweight midvariance – a robust and efficient measure of spread.

Note that many small segments will fit noisy data better, shrinking the residuals used to calculate the other estimates of spread, even if many of the segments are spurious. One possible heuristic for judging the overall noisiness of each sample in a table is to multiply the number of segments by the biweight midvariance – the value will tend to be higher for unreliable samples. Check questionable samples for poor coverage (using e.g. bedtools, chanjo, IGV or Picard CalculateHsMetrics).

Finally, visualizing a sample with CNVkit's *scatter* command will often make it apparent whether a sample or the copy ratios within a genomic region can be trusted.

2.3.5 segmetrics

Calculate summary statistics of the residual bin-level log2 ratio estimates from the segment means, similar to the existing *metrics* command, but for each segment individually.

Results are output in the same format as the CNVkit segmentation file (.cns), with the stat names and calculated values printed in additional columns.

```
cnvkit.py segmetrics Sample.cnr -s Sample.cns --iqr
cnvkit.py segmetrics -s Sample.cn{s,r} --ci --pi
```

Supported stats:

- As in *metrics*: standard deviation (`--std`), median absolute deviation (`--mad`), inter-quartile range (`--iqr`), Tukey's biweight midvariance (`--bivar`)
- confidence interval (`--ci`), estimated by bootstrap (100 resamples)
- prediction interval (`--pi`), estimated by the range between the 2.5-97.5 percentiles of bin-level log2 ratio values within the segment.

2.4 Compatibility and other I/O

2.4.1 import-picard

Convert Picard CalculateHsMetrics per-target coverage files (.csv) to the CNVkit .cnn format:

```
cnvkit.py import-picard *.hsmetrics.targetcoverages.csv *.hsmetrics.antitargetcoverages.csv
cnvkit.py import-picard picard-hsmetrics/ -d cnvkit-from-picard/
```

You can use Picard tools to perform the bin read depth and GC calculations that CNVkit normally performs with the *coverage* and *reference* commands, if need be.

Procedure:

1. Use the *target* and *antitarget* commands to generate the "targets.bed" and "antitargets.bed" files.
2. Convert those BED files to Picard's "interval list" format by adding the BAM header to the top of the BED file and rearranging the columns – see the Picard command BedToIntervalList.
3. Run Picard CalculateHsMetrics on each of your normal/control BAM files with the "targets" and "antitargets" interval lists (separately), your reference genome, and the "PER_TARGET_COVERAGE" option.
4. Use *import-picard* to convert all of the PER_TARGET_COVERAGE files to CNVkit's .cnn format.
5. Use *reference* to build a CNVkit reference from those .cnn files. It will retain the GC values Picard calculated; you don't need to provide the reference genome sequence again to get GC (but you if you do, it will also calculate the RepeatMaster fraction values)
6. Use *batch* with the `-r`/`--reference` option to process the rest of your test samples.

2.4.2 import-seg

Convert a file in the SEG format (e.g. the output of standard CBS or the GenePattern server) into one or more CNVkit .cns files.

The chromosomes in a SEG file may have been converted from chromosome names to integer IDs. Options in `import-seg` can help recover the original names.

- To add a "chr" prefix, use "-p chr".
- To convert chromosome indices 23, 24 and 25 to the names "X", "Y" and "M" (a common convention), use "-c human".
- To use an arbitrary mapping of indices to chromosome names, use a comma-separated "key:value" string. For example, the human convention would be: "-c 23:X,24:Y,25:M".

2.4.3 import-theta

Convert the ".results" output of THetA2 to one or more CNVkit .cns files representing subclones with integer absolute copy number in each segment.

2.4.4 export

Convert copy number ratio tables (.cnr files) or segments (.cns) to another format.

bed

Segments can be exported to BED format to support a variety of other uses, such as viewing in a genome browser. The log2 ratio value of each segment is converted and rounded to an integer value, as required by the BED format. To get accurate copy number values, see the commands *rescale* and *call*.

```
# Estimate integer copy number of each segment
cnvkit.py call Sample.cns -y -o Sample.call.cns
# Show estimated integer copy number of all regions
cnvkit.py export bed Sample.call.cns --show all -y -o Sample.bed
```

The same format can also specify CNV regions to the FreeBayes variant caller with FreeBayes's `--cnv-map` option:

```
# Show only CNV regions
cnvkit.py export bed Sample.call.cns -o all-samples.cnv-map.bed
```

By default only regions with copy number different from the given ploidy (default 2) are output. (Notice what this means for allosomes.) To output all segments, use the `--show all` option.

vcf

Convert segments, ideally already adjusted by the *rescale* and/or *call* commands, to a *VCF* file. Copy ratios are converted to absolute integers, as with BED export, and VCF records are created for the segments where the copy number is different from the expected ploidy (e.g. 2 on autosomes, 1 on haploid sex chromosomes, depending on sample gender).

Gender can be specified with the `-g`/`--gender` option, or will be guessed automatically. If a male reference is used, use `-y`/`--male-reference` to say so. Note that these are different: If a female sample is run with a male reference, segments on chromosome X with log2-ratio +1 will be skipped, because that's the expected copy number, while an X-chromosome segment with log2-ratio 0 will be printed as a hemizygous loss.

```
cnvkit.py export vcf Sample.cns -y -g female -i "SampleID" -o Sample.cnv.vcf
```

cdt, jtv

A collection of probe-level copy ratio files (`*.cnr`) can be exported to Java TreeView via the standard CDT format or a plain text table:

```
cnvkit.py export jtv *.cnr -o Samples-JTV.txt
cnvkit.py export cdt *.cnr -o Samples.cdt
```

seg

Similarly, the segmentation files for multiple samples (`*.cns`) can be exported to the standard SEG format to be loaded in the Integrative Genomic Viewer (IGV):

```
cnvkit.py export seg *.cns -o Samples.seg
```

nexus-basic

The format `nexus-basic` can be loaded directly by the commercial program Biodiscovery Nexus Copy Number, specifying the "basic" input format in that program. This allows viewing CNVkit data as if it were from array CGH.

This is a tabular format very similar to .cnr files, with the columns:

1. chromosome
2. start
3. end
4. log2

nexus-ogt

The format `nexus-ogt` can be loaded directly by the commercial program Biodiscovery Nexus Copy Number, specifying the "Custom-OGT" input format in that program. This allows viewing CNVkit data as if it were from a SNP array.

This is a tabular format similar to .cnr files, but with B-allele frequencies (BAFs) extracted from a corresponding VCF file. The format's columns are (with .cnr equivalents):

1. "Chromosome" (chromosome)
2. "Position" (start)
3. "Position" (end)
4. "Log R Ratio" (log2)
5. "B-Allele Frequency" (from VCF)

The positions of each heterozygous variant record in the given VCF are matched to bins in the given .cnr file, and the variant allele frequencies are extracted and assigned to the matching bins.

- If a bin contains no variants, the BAF field is left blank
- If a bin contains multiple variants, the BAFs of those variants are "mirrored" to be all above .5 (e.g. BAF of .3 becomes .7), then the median is taken as the bin-wide BAF.

2.4.5 version

Print CNVkit's version as a string on standard output:

```
cnvkit.py version
```

2.5 Additional scripts

refFlat2bed.py Generate a BED file of the genes or exons in the reference genome given in UCSC refFlat.txt format. (Download the input file from UCSC Genome Bioinformatics).

This script can be used in case the original BED file of targeted intervals is unavailable. Subsequent steps of the pipeline will remove probes that did not receive sufficient coverage, including those exons or genes that were not targeted by the sequencing library. However, CNVkit will give much better results if the true targeted intervals can be provided.

reference2targets.py Extract target and antitarget BED files from a CNVkit reference file. While the *batch* command does this step automatically when an existing reference is provided, you may find this standalone script useful to recover the target and antitarget BED files that match the reference if those BED files are missing or you're not sure which ones are correct.

Alternatively, once you have a stable CNVkit reference for your platform, you can use this script to drop the "bad" bins from your target and antitarget BED files (and subsequently built references) to avoid unnecessarily calculating coverage in those bins during future runs.

CHAPTER

THREE

FAQ & HOW TO

3.1 File formats

We've tried to use standard file formats where possible in CNVkit. However, in a few cases we have needed to extend the standard BED format to accommodate additional information.

All of the non-standard file formats used by CNVkit are tab-separated plain text and can be loaded in a spreadsheet program, R or other statistical analysis software for manual analysis, if desired.

3.1.1 BED and GATK/Picard Interval List

- UCSC Genome Browser's BED definition and FAQ
- GATK's Interval List description and FAQ

Note that BED genomic coordinates are 0-indexed, like C or Python code – for example, the first nucleotide of a 1000-basepair sequence has position 0, the last nucleotide has position 999, and the entire region is indicated by the range 0-1000.

Interval list coordinates are 1-indexed, like R or Matlab code. In the same example, the first nucleotide of a 1000-basepair sequence has position 1, the last nucleotide has position 1000, and the entire region is indicated by the range 1-1000.

3.1.2 VCF

See the VCF specifications.

CNVkit currently uses VCF files in two ways:

- To *export* CNVs, describing/encoding each CNV segment as a structural variant (SV).
- To plot single-nucleotide variant (SNV) allele frequencies in the *scatter* and *loh* commands, or export these allele frequencies to the "nexus-ogt" format.

3.1.3 Target and antitarget bin-level coverages (.cnn)

Coverage of binned regions is saved in a tabular format similar to BED but with additional columns. Each row in the file indicates an on-target or off-target (antitarget, background) bin. Genomic coordinates are 0-indexed, like BED.

Column names are shown as the first line of the file:

- Chromosome or reference sequence name (`chromosome`)

- Start position (`start`)
- End position (`end`)
- Gene name (`gene`)
- Log2 mean coverage depth (`log2`)

Essentially the same tabular file format is used for coverages (.cnn), ratios (.cnr) and segments (.cns) emitted by CNVkit.

3.1.4 Copy number reference profile (.cnn)

In addition to the columns present in the "target" and "antitarget" .cnn files, the reference .cnn file has the columns:

- GC content of the sequence region (`gc`)
- RepeatMasker-masked proportion of the sequence region (`rmask`)
- Statistical spread or dispersion (`spread`)

The log2 coverage depth is the weighted average of coverage depths, excluding extreme outliers, observed at the corresponding bin in each the sample .cnn files used to construct the reference. The spread is a similarly weighted estimate of the standard deviation of normalized coverages in the bin.

To manually review potentially problematic genes in the built reference, you can sort the file by the "spread" column; bins with higher values are the noisy ones.

It is important to keep the copy number reference file consistent for the duration of a project, reusing the same reference for bias correction of all tumor samples in a cohort. If your library preparation protocol changes, it's usually best to build a new reference file and use the new file to analyze the samples prepared under the new protocol.

3.1.5 Bin-level log2 ratios (.cnr)

In addition to the BED-like `chromosome`, `start`, `end` and `gene` columns present in .cnn files, the .cnr file has the columns:

- Log2 ratio (`log2`)
- Proportional weight to be used for segmentation (`weight`)

The weight value is the inverse of the variance (i.e. square of `spread` in the reference) of normalized log2 coverage values seen among all normal samples at that bin. This value is used to weight the bin log2 ratio values during segmentation.

Also, when a genomic region is plotted with CNVkit's "scatter" command, the size of the plotted datapoints is proportional to the weight of each point used in segmentation – a relatively small point indicates a less reliable bin.

3.1.6 Segmented log2 ratios (.cns)

In addition to the `chromosome`, `start`, `end`, `gene` and `log2` columns present in .cnr files, the .cns file format has the additional column `probes`, indicating the number of bins covered by the segment.

The `gene` column does not contain actual gene names. Rather, the sign of the segment's copy ratio value is indicated by 'G' if greater than zero or 'L' if less than zero.

3.2 Bias corrections

The sequencing coverage depth obtained at different genomic regions is variable, particularly for targeted capture. Much of this variability is due to known biochemical effects related to library prep, target capture and sequencing. Normalizing the read depth in each on– and off-target bin to the expected read depths derived from a reference of normal samples removes much of the biases attributable to GC content, target density. However, these biases also vary between samples, and must still be corrected even when a normal reference is available.

To correct each of these known effects, CNVkit calculates the relationship between observed bin-level read depths and the values of some known biasing factor, such as GC content. This relationship is fitted using a simple rolling median, then subtracted from the original read depths in a sample to yield corrected estimates.

In the case of many similarly sized target regions, there is the potential for the bias value to be identical for many targets, including some spatially near each other. To ensure that the calculated biases are independent of genomic position, the probes are randomly shuffled before being sorted by bias value.

The GC content and repeat-masked fraction of each bin are calculated during generation of the *reference* from the user-supplied genome. The bias corrections are then performed in the *reference* and *fix* commands.

3.2.1 GC content

Genomic regions with extreme GC content, the fraction of sequence composed of guanine or cytosine bases, are less amenable to hybridization, amplification and sequencing, and will generally appear to have lower coverage than regions of average GC content.

To correct this bias in each sample, CNVkit calculates the association between each bin's GC content (stored in the reference) and observed read depth, fits a trendline through the bin read depths ordered by GC value, and subtracts this trend from the original read depths.

3.2.2 Sequence repeats

Repetitive elements in the genome can be masked out with RepeatMasker – and the genome sequences provided by the UCSC Genome Bioinformatics Site have this masking applied already. The fraction of each genomic bin masked out for repetitiveness indicates both low mappability and the susceptibility to Cot-1 blocking, both of which can reduce the bin's observed coverage.

CNVkit removes the association between repeat-masked fraction and bin read depths for each sample similarly to the GC correction.

3.2.3 Targeting density

In hybridization capture, two biases occur near the edge of each baited region:

- Within the baited region, read depth is lower at the "shoulders" where sequence fragments are not completely captured.
- Just outside the baited region, in the "flanks", read depth is elevated to nearly that of the adjacent baited sites due to the same effect. If two targets are very close together, the sequence fragments captured for one target can increase the read depth in the adjacent target.

CNVkit roughly estimates the potential for these two biases based on the size and position of each baited region and its immediate neighbors. The biases are modeled together as a linear decrease in read depth from inside the target region to the same distance outside. These biases occur within a distance of the interval edges equal to the sequence fragment size (also called the insert size for paired-end sequencing reads). Density biases are calculated from the start

and end positions of a bin and its neighbors within a fixed window around the target's genomic coordinates equal to the sequence fragment size.

Shoulder effect: Letting i be the average insert size and t be the target interval size, the negative bias at interval shoulders is calculated as $i/4t$ at each side of the interval, or $i/2t$ for the whole interval. When the interval is smaller than the sequence fragment size, the portion of the fragment extending beyond the opposite edge of the interval should not be counted in this calculation. Thus, if $t < i$, the negative bias value must be increased (absolute value reduced) by $\frac{(i-t)^2}{2it}$.

Flank effect: Additionally letting g be the size of the gap between consecutive intervals, the positive bias that occurs when the gap is smaller than the insert size ($g < i$) is $\frac{(i-g)^2}{4it}$. If the target interval and gap together are smaller than the insert size, the reads flanking the neighboring interval may extend beyond the target, and this flanking portion beyond the target should not be counted. Thus, if $t + g < i$, the positive value must be reduced by $\frac{(i-g-t)^2}{4it}$. If a target has no close neighbors ($g > i$, the common case), the "flank" bias value is 0.

These values are combined into a single value by subtracting the estimated shoulder biases from the flank biases. The result is a negative number between -1 and 0, or 0 for a target with immediately adjacent targets on both sides. Thus, subdividing a large targeted interval into a consecutive series of smaller targets does not change the net "density" calculation value.

The association between targeting density and bin read depths is then fitted and subtracted, as with GC and RepeatMasker.

CNVkit applies the density bias correction to only the on-target bins; the negative "shoulder" bias is not expected to occur in off-target regions because those regions are not specifically captured by baits, and the positive "flank" bias from neighboring targets is avoided by allocating off-target bins around existing targets with a margin of twice the expected insert size.

3.3 Calling absolute copy number

The relationship between the observed copy ratio and the true underlying copy number depends on tumor cell fraction (purity), genome ploidy (which may be heterogeneous in a tissue sample), and the size of the subclonal population containing the CNA. Because of these ambiguities, CNVkit only reports the estimated log2 copy ratio, and does not currently attempt a formal statistical test for estimating integer copy number.

In a diploid genome, a single-copy gain in a perfectly pure, homogeneous sample has a copy ratio of 3/2. In log2 scale, this is log2(3/2) = 0.585, and a single-copy loss is log2(1/2) = -1.0.

In the *diagram* plot, for the sake of providing a clean visualization of confidently called CNAs, the default threshold to label genes is 0.5. This threshold will tend to display gene amplifications, fully clonal single-copy gains in fairly pure samples, most single-copy losses, and complete deletions.

When using the *gainloss* command, choose a threshold to suit your needs depending on your knowledge of the sample's purity, heterogeneity, and likely features of interest. As a starting point, try 0.1 or 0.2 if you are going to do your own filtering downstream, or 0.3 if not.

The *call* command implements two simple methods to convert the log2 ratios in a segmented .cns file to absolute integer copy number values. The *segmetrics* command computes segment-level summary statistics that can be used to evaluate the reliability of each segment. Future releases of CNVkit will integrate further statistical testing to help make meaningful variant calls from the log2 copy ratio data.

After using *rescale* and/or *call*, the adjusted .cns file can then be converted to BED or VCF format using the *export* command. These output styles display the inferred absolute integer copy number value of each segment.

3.4 Tumor heterogeneity

DNA samples extracted from solid tumors are rarely completely pure. Stromal or other normal cells and distinct subclonal tumor-cell populations are typically present in a sample, and can confound attempts to fit segmented log2 ratio values to absolute integer copy numbers.

CNVkit provides several points of integration with existing tools and methods for dealing with tumor heterogeneity and normal-cell contamination.

3.4.1 Estimating tumor purity and normal contamination

A rough estimate of tumor purity can usually be obtained using one or more of these approaches:

1. A pathologist can visually estimate the purity of an sample taken from a solid tumor by examination under a microscope, counting stromal and neoplastic cells.
2. If the tumor is belived to be driven by a somatic point mutation, e.g. BRAF V600E in melanoma, then that mutation is assumed to be fully clonal and its allele frequency indicates the tumor purity. This can be complicated by copy number alterations at the same site and whether the point mutation is homozygous or heterozygous, but the frequencies of other somatic mutations in the same sample may resolve this satisfactorily.
3. Larger-scale, hemizygous losses that cover germline heterozygous SNPs shift the allele frequencies of the same SNPs as they are present in the tumor sample. In a 50% pure tumor sample, for example, these SNP b-allele frequencies would shift from 50% to 67% or 33%, assuming a diploid sample (i.e. 1 of 2 copies from the normal sample and 0 or 1 of 1 copy from the tumor, depending on whether the variant allele was lost or retained). The general calculation is a bit more complicated than in #1 or #2, and can be done similarly for copy number gains and homozygous deletions.
4. The log2 ratio values of CNAs in a tumor sample correspond to integer copy numbers in tumor cells, and in aggregate these log2 values will cluster around values that indicate subclone populations, each with a given ploidy and clonality. For example, a single-copy loss in a 50% pure tumor sample will have 3/4 the coverage of a neutral site (2/2 normal copies, 1/2 tumor copies), for a log2 value of log2(.75) = -0.415. This calculation can also be generalized to other copy number states.

Software implementations of the latter three approaches can be used directly on DNA sequencing data.

3.4.2 Inferring tumor purity and subclonal population fractions from sequencing

While inferring the tumor population structure is currently out of the scope of CNVkit, this work can be done using other third-party programs such as THetA2, PyClone, or BubbleTree. Each of these programs can be used to estimate tumor cell content and infer integer copy number of tumor subclones in a sample.

Using CNVkit with THetA2

CNVkit provides wrappers for exporting segments to THetA2's input format and importing THetA2's result file as CNVkit's segmented .cns files.

THetA2's input file is a BED-like file, typically with the extension `.input`, listing the read counts within each copy-number segment in a pair of tumor and normal samples. CNVkit can generate this file given the CNVkit-inferred tumor segmentation (.cns) and normal copy log2-ratios (.cnr) or copy number reference file (.cnn). This bypasses the initial step of THetA2, CreateExomeInput, which counts the reads in each sample's BAM file.

After running the CNVkit Copy number calling pipeline on a sample, create the THetA2 input file:

```
# From a paired normal sample
cnvkit.py export theta Sample_Tumor.cns Sample_Normal.cnr -o Sample.theta2.input
# From an existing CNVkit reference
cnvkit.py export theta Sample_Tumor.cns reference.cnn -o Sample.theta2.input
```

Then, run THetA2 (assuming the program was unpacked at `/path/to/theta2/`):

```
# Generates Sample.theta2.BEST.results:
/path/to/theta2/bin/RunTHetA Sample.theta2.input
# Parameters for low-quality samples:
/path/to/theta2/python/RunTHetA.py Sample.theta2.input -n 2 -k 4 -m .90 --FORCE --NUM_PROCESSES `npro
```

Finally, import THetA2's results back into CNVkit's .cns format, matching the original segmentation (.cns) to the THetA2-inferred absolute copy number values.:

```
cnvkit.py import-theta Sample_Tumor.cns Sample.theta2.BEST.results
```

THetA2 adjusts the segment log2 values to the inferred cellularity of each detected subclone; this can result in one or two .cns files representing subclones if more than one clonal tumor cell population was detected. THetA2 also performs some significance testing of each segment representing a CNA, so there may be fewer segments derived from THetA2 than were originally found by CNVkit.

The segment values are still log2-transformed in the resulting .cns files, for convenience in plotting etc. with CNVkit. These files are also easily converted to other formats using the *export* command.

3.4.3 Adjusting copy ratios and segments for normal cell contamination

CNVkit's *rescale* command uses an estimate of tumor fraction (from any source) to directly rescale segment log2 ratio values to the value that would be seen a completely pure, uncontaminated sample. Example with tumor purity of 60% and a male reference:

```
cnvkit.py rescale Sample.cns --purity 0.6 -y -o Sample.rescale.cns
```

CNVkit's *call* command then converts the segmented log2 ratio estimates to absolute integer copy numbers. Note that the rescaling step is optional; either way, hard thresholds can be used:

```
# With CNVkit's default cutoffs
cnvkit.py call -m threshold Sample.cns -y -o Sample.call.cns
# Or, using a custom set of cutoffs
cnvkit.py call -t=-1.1,-0.4,0.3,0.7 Sample.cns -y -o Sample.call.cns
```

Alternatively, if the tumor cell fraction is known confidently, then use the `clonal` method to simply round the log2 ratios to the nearest integer copy number:

```
cnvkit.py call -m clonal Sample.cns -y --purity 0.65 -o Sample.call.cns
# Or, if already rescaled
cnvkit.py call -m clonal Sample.rescale.cns -y -o Sample.call.cns
```

3.4.4 Export integer copy numbers as BED or VCF

The *export* `bed` command emits integer copy number calls in standard BED format:

```
cnvkit.py export bed Sample.call.cns -y -o Sample.bed
cnvkit.py export vcf Sample.call.cns -y -o Sample.vcf
```

The rounding of the .cns file's log2 ratios to integer copy numbers here is the same as in the *call* command with the `clonal` method.

3.5 Whole-genome sequencing and targeted amplicon capture

CNVkit is designed for use on **hybrid capture** sequencing data, where off-target reads are present and can be used improve copy number estimates.

If necessary, CNVkit can be used on **whole-genome sequencing** (WGS) datasets by specifying the genome's sequencing-accessible regions as the "targets", avoiding "antitargets", and using a gene annotation database to label genes in the resulting BED file:

```
cnvkit.py batch ... -t data/access-5kb-mappable.hg19.bed -g data/access-5kb-mappable.hg19.bed --split
```

Or:

```
cnvkit.py target data/access-5kb-mappable.hg19.bed --split --annotate refFlat.txt -o Targets.bed
cnvkit.py antitarget data/access-5kb-mappable.hg19.bed -g data/access-5kb-mappable.hg19.bed -o Backgr
```

This produces a "target" binning of the entire sequencing-accessible area of the genome, and empty "antitarget" files which CNVkit will handle safely from version 0.3.4 onward.

Similarly, to use CNVkit on **targeted amplicon sequencing** data instead – although this is not recommended – you can exclude all off-target regions from the analysis by passing the target BED file as the "access" file as well:

```
cnvkit.py batch ... -t Targeted.bed -g Targeted.bed ...
```

Or:

```
cnvkit.py antitarget Targeted.bed -g Targeted.bed -o Background.bed
```

However, this approach does not collect any copy number information between targeted regions, so it should only be used if you have in fact prepared your samples with a targeted amplicon sequencing protocol. It also does not attempt to normalize each amplicon at the gene level, though this may be addressed in a future version of CNVkit.

CHAPTER

FOUR

PYTHON API

4.1 Python API (cnvlib package)

4.1.1 Module `cnvlib` contents

`cnvlib.`**`read`**(*fname*)
Parse a file as a copy number or copy ratio table (.cnn, .cnr).

The one function exposed at the top level, read, loads a file in CNVkit's native format and returns a CopyNumArray instance. For your own scripting, you can usually accomplish what you need using the CopyNumArray and GenomicArray methods available on this returned object.

4.1.2 Core classes

The core objects used throughout CNVkit. The base class is GenomicArray. All of these classes wrap a pandas DataFrame instance accessible through the `.data` attribute which can be used for any manipulations that aren't already provided by methods in the wrapper class.

gary

A generic array of genomic positions.

class `cnvlib.gary.`**`GenomicArray`**(*data_table*, *meta_dict=None*)
Bases: `object`

An array of genomic intervals. Base class for genomic data structures.

Can represent most BED-like tabular formats with arbitrary additional columns.

`add`(*other*)
Combine this array's data with another GenomicArray (in-place).

Any optional columns must match between both arrays.

`add_columns`(***columns*)
Create a new CNA, adding the specified extra columns to this CNA.

`as_columns`(***columns*)
Wrap the named columns in this instance's metadata.

`as_dataframe`(*dframe*)
Wrap the given pandas dataframe in this instance's metadata.

as_rows (*rows*)
Wrap the given rows in this instance's metadata.

autosomes (*also=()*)
Select chromosomes w/ integer names, ignoring any 'chr' prefixes.

by_chromosome ()
Iterate over bins grouped by chromosome name.

by_ranges (*other*, *mode='inner'*, *keep_empty=True*)
Group rows by another GenomicArray's bin coordinate ranges.

Returns an iterable of (bin, GenomicArray of overlapping rows)). Usually used for grouping probes or SNVs by CNV segments.

mode determines what to do with bins that overlap a boundary of the selection. Values are:

- `inner`: Drop the bins on the selection boundary, don't emit them.
- `outer`: Keep/emit those bins as they are.
- `trim`: Emit those bins but alter their boundaries to match the selection; the bin start or end position is replaced with the selection boundary position. [default]

Bins in this array that fall outside the other array's bins are skipped.

chromosome

concat (*others*)
Concatenate several GenomicArrays, keeping this array's metadata.

This array's data table is not implicitly included in the result.

coords (*also=()*)
Iterate over plain coordinates of each bin: chromosome, start, end.

With *also*, also include those columns.

Example, yielding rows in BED format:

```
>>> probes.coords(also=["name", "strand"])
```

copy ()
Create an independent copy of this object.

drop_extra_columns ()
Remove any optional columns from this GenomicArray.

Returns a new copy with only the core columns retained: log2 value, chromosome, start, end, bin name.

end

classmethod **from_columns** (*columns*, *meta_dict=None*)
Create a new instance from column arrays, given as a dict.

classmethod **from_rows** (*rows*, *columns=None*, *meta_dict=None*)
Create a new instance from a list of rows, as tuples or arrays.

in_range (*chrom=None*, *start=None*, *end=None*, *mode='inner'*)
Get the GenomicArray portion within the given genomic range.

mode works as in *by_ranges*: `outer` includes bins straddling the range boundaries, `trim` additionally alters the straddling bins' endpoints to match the range boundaries, and `inner` excludes those bins.

in_ranges(*chrom=None*, *starts=None*, *ends=None*, *mode='inner'*)
Get the GenomicArray portion within the given array's ranges.

keep_columns(*columns*)
Extract a subset of columns, reusing this instance's metadata.

labels()

match_to_bins(*other*, *key*, *default=0.0*, *fill=False*, *summary_func=<function median>*)
Take values of the other array at each of this array's bins.

Assign *default* to indices that fall outside the other array's bins, or chromosomes that appear in *self* but not *other*.

Return an array of the *key* column values in *other* corresponding to this array's bin locations, the same length as this array.

classmethod read(*infile*, *sample_id=None*)

static row2label(*row*)

sample_id

select(*selector=None*, ***kwargs*)
Take a subset of rows where the given condition is true.

Arguments can be a function (lambda expression) returning a bool, which will be used to select True rows, and/or keyword arguments like gene="Background" or chromosome="chr7", which will select rows where the keyed field equals the specified value.

shuffle()
Randomize the order of bins in this array (in-place).

sort()
Sort this array's bins in-place, with smart chromosome ordering.

sort_columns()
Sort this array's columns in-place, per class definition.

start

write(*outfile=None*)
Write the wrapped data table to a file or handle in tabular format.

The format is BED-like, but with a header row included and with arbitrary extra columns.

To combine multiple samples in one file and/or convert to another format, see the 'export' subcommand.

cnary

CNVkit's core data structure, a copy number array.

class cnvlib.cnary.**CopyNumArray**(*data_table*, *meta_dict=None*)
Bases: cnvlib.gary.GenomicArray

An array of genomic intervals, treated like aCGH probes.

Required columns: chromosome, start, end, gene, log2

Optional columns: gc, rmask, spread, weight, probes

by_gene(*ignore=('-', '.', 'CGH')*)
Iterate over probes grouped by gene name.

Emits pairs of (gene name, CNA of rows with same name)

Groups each series of intergenic bins as a ‘Background’ gene; any ‘Background’ bins within a gene are grouped with that gene. Bins with names in *ignore* are treated as ‘Background’ bins, but retain their name.

`center_all`(*estimator=<function median>*)
Recenter coverage values to the autosomes’ average (in-place).

`drop_low_coverage`()
Drop bins with extremely low log2 coverage values.

These are generally bins that had no reads mapped, and so were substituted with a small dummy log2 value to avoid divide-by-zero errors.

`expect_flat_cvg`(*is_male_reference=None*)
Get the uninformed expected copy ratios of each bin.

Create an array of log2 coverages like a “flat” reference.

This is a neutral copy ratio at each autosome (log2 = 0.0) and sex chromosomes based on whether the reference is male (XX or XY).

`get_relative_chrx_cvg`()
Get the relative log-coverage of chrX in a sample.

`guess_average_depth`(*segments=None*, *window=100*)
Estimate the effective average read depth from variance.

Assume read depths are Poisson distributed, converting log2 values to absolute counts. Then the mean depth equals the variance , and the average read depth is the estimated mean divided by the estimated variance. Use robust estimators (Tukey’s biweight location and midvariance) to compensate for outliers and overdispersion.

With *segments*, take the residuals of this array’s log2 values from those of the segments to remove the confounding effect of real CNVs.

If *window* is an integer, calculate and subtract a smoothed trendline to remove the effect of CNVs without segmentation (skipped if *segments* are given).

See: http://www.evanmiller.org/how-to-read-an-unlabeled-sales-chart.html

`guess_xx`(*male_reference=False*, *verbose=True*)
Guess whether a sample is female from chrX relative coverages.

Recommended cutoff values: -0.5 – raw target data, not yet corrected +0.5 – probe data already corrected on a male profile

`log2`

`residuals`(*segments=None*)
Difference in log2 value of each bin from its segment mean.

If segments are just regions (e.g. RegionArray) with no log2 values precalculated, subtract the median of this array’s log2 values within each region. If no segments are given, subtract each chromosome’s median.

`shift_xx`(*male_reference=False*)
Adjust chrX coverages (divide in half) for apparent female samples.

`squash_genes`(*summary_func=<function biweight_location>*, *squash_background=False*, *ignore=(‘-‘, ‘.’, ‘CGH’)*)
Combine consecutive bins with the same targeted gene name.

The *ignore* parameter lists bin names that not be counted as genes to be output.

Parameter *summary_func* is a function that summarizes an array of coverage values to produce the “squashed” gene’s coverage value. By default this is the biweight location, but you might want median, mean, max, min or something else in some cases.

rary

An array of genomic regions or features.

class cnvlib.rary.**RegionArray**(*data_table*, *meta_dict=None*)

Bases: *cnvlib.gary.GenomicArray*

An array of genomic intervals.

classmethod read(*fname*, *sample_id=None*, *fmt=None*)

Read regions in any of the expected file formats.

Iterates over tuples of the tabular contents. Header lines are skipped.

Start and end coordinates are base-0, half-open.

write(*outfile=<open file '<stdout>', mode 'w'>*, *fmt='bed'*, *verbose=True*)

vary

An array of genomic intervals, treated as variant loci.

class cnvlib.vary.**VariantArray**(*data_table*, *meta_dict=None*)

Bases: *cnvlib.gary.GenomicArray*

An array of genomic intervals, treated as variant loci.

Required columns: chromosome, start, end, ref, alt

mirrored_baf(*above_half=True*, *tumor_boost=True*)

Mirrored B-allele frequencies (BAFs).

Flip BAFs to be all above 0.5 (if *above_half*) or below 0.5, for consistency.

With *tumor_boost*, normalize tumor-sample allele frequencies to the matched normal allele frequencies.

classmethod read_vcf(*infile*, *sample_id=None*, *normal_id=None*, *min_depth=None*, *skip_hom=False*, *skip_reject=False*, *skip_somatic=False*)

Parse SNV coordinates from a VCF file into a VariantArray.

tumor_boost()

TumorBoost normalization of tumor-sample allele frequencies.

De-noises the signal for detecting LOH.

4.1.3 Interface to CNVkit sub-commands

commands

The public API for each of the commands defined in the CNVkit workflow. Command-line interface and corresponding API for CNVkit.

cnvlib.commands.**batch_make_reference**(*normal_bams*, *target_bed*, *antitarget_bed*, *male_reference*, *fasta*, *annotate*, *short_names*, *split*, *target_avg_size*, *access*, *antitarget_avg_size*, *antitarget_min_size*, *output_reference*, *output_dir*, *processes*, *by_count*)

Build the CN reference from normal samples, targets and antitargets.

cnvlib.commands.**batch_run_sample**(*bam_fname*, *target_bed*, *antitarget_bed*, *ref_fname*, *output_dir*, *male_reference=False*, *scatter=False*, *diagram=False*, *rlibpath=None*, *by_count=False*)
Run the pipeline on one BAM file.

cnvlib.commands.**batch_write_coverage**(*bed_fname*, *bam_fname*, *out_fname*, *by_count*)
Run coverage on one sample, write to file.

cnvlib.commands.**csvstring**(*text*)

cnvlib.commands.**do_access**(*fa_fname*, *exclude_fnames=()*, *min_gap_size=5000*)
List the locations of accessible sequence regions in a FASTA file.

cnvlib.commands.**do_antitarget**(*target_bed*, *access_bed=None*, *avg_bin_size=100000*, *min_bin_size=None*)
Derive a background/antitarget BED file from a target BED file.

cnvlib.commands.**do_breaks**(*probes*, *segments*, *min_probes=1*)
List the targeted genes in which a copy number breakpoint occurs.

cnvlib.commands.**do_call**(*segments*, *method*, *ploidy=2*, *purity=None*, *is_reference_male=False*, *is_sample_female=False*, *thresholds=(-1.1*, *-0.25*, *0.2*, *0.7)*)

cnvlib.commands.**do_coverage**(*bed_fname*, *bam_fname*, *by_count=False*, *min_mapq=0*)
Calculate coverage in the given regions from BAM read depths.

cnvlib.commands.**do_fix**(*target_raw*, *antitarget_raw*, *reference*, *do_gc=True*, *do_edge=True*, *do_rmask=True*)
Combine target and antitarget coverages and correct for biases.

cnvlib.commands.**do_gainloss**(*probes*, *segments=None*, *male_reference=False*, *threshold=0.2*, *min_probes=3*, *skip_low=False*)
Identify targeted genes with copy number gain or loss.

cnvlib.commands.**do_heatmap**(*cnarrs*, *show_range=None*, *do_desaturate=False*)
Plot copy number for multiple samples as a heatmap.

cnvlib.commands.**do_reference**(*target_fnames*, *antitarget_fnames*, *fa_fname=None*, *male_reference=False*)
Compile a coverage reference from the given files (normal samples).

cnvlib.commands.**do_reference_flat**(*targets*, *antitargets*, *fa_fname=None*, *male_reference=False*)
Compile a neutral-coverage reference from the given intervals.

Combines the intervals, shifts chrX values if requested, and calculates GC and RepeatMasker content from the genome FASTA sequence.

cnvlib.commands.**do_rescale**(*cnarr*, *ploidy=2*, *purity=None*, *is_reference_male=False*, *is_sample_female=False*)

cnvlib.commands.**do_scatter**(*cnarr*, *segments=None*, *variants=None*, *show_range=None*, *show_gene=None*, *background_marker=None*, *do_trend=False*, *window_width=1000000.0*, *y_min=None*, *y_max=None*, *title=None*)
Plot probe log2 coverages and CBS calls together.

show_gene: name of gene to highligh show_range: chromosome name or coordinate string like "chr1:20-30"

cnvlib.commands.**do_targets**(*bed_fname*, *annotate=None*, *do_short_names=False*, *do_split=False*, *avg_size=266.6666666666667*)
Transform bait intervals into targets more suitable for CNVkit.

cnvlib.commands.**parse_args**(*args=None*)
Parse the command line.

`cnvlib.commands.`**`print_version`**`(`*_args*`)`
Display this program's version.

`cnvlib.commands.`**`verify_gender_arg`**`(`*cnarr*, *gender_arg*, *is_male_reference*`)`

The following modules implement lower-level functionality specific to each of the CNVkit sub-commands.

antitarget

Supporting functions for the 'antitarget' command.

`cnvlib.antitarget.`**`find_background_regions`**`(`*access_chroms*, *target_chroms*, *pad_size*`)`
Take coordinates of accessible regions and targets; emit antitargets.

`cnvlib.antitarget.`**`get_background`**`(`*target_bed*, *access_bed*, *avg_bin_size*, *min_bin_size*`)`
Generate background intervals from target intervals.

Procedure:

- Invert target intervals
- Subtract the inverted targets from accessible regions
- For each of the resulting regions:
  - Shrink by a fixed margin on each end
  - If it's smaller than min_bin_size, skip
  - Divide into equal-size (region_size/avg_bin_size) portions
  - Emit the (chrom, start, end) coords of each portion

`cnvlib.antitarget.`**`guess_chromosome_regions`**`(`*target_chroms*, *telomere_size*`)`
Determine (minimum) chromosome lengths from target coordinates.

call

Call copy number variants from segmented log2 ratios.

`cnvlib.call.`**`absolute_clonal`**`(`*cnarr*, *ploidy*, *purity*, *is_reference_male*, *is_sample_female*`)`
Calculate absolute copy number values from segment or bin log2 ratios.

`cnvlib.call.`**`absolute_dataframe`**`(`*cnarr*, *ploidy*, *purity*, *is_reference_male*, *is_sample_female*`)`
Absolute, expected and reference copy number in a DataFrame.

`cnvlib.call.`**`absolute_pure`**`(`*cnarr*, *ploidy*, *is_reference_male*`)`
Calculate absolute copy number values from segment or bin log2 ratios.

`cnvlib.call.`**`absolute_threshold`**`(`*cnarr*, *ploidy*, *thresholds*, *is_reference_male*`)`
Call integer copy number using hard thresholds for each level.

Integer values are assigned for log2 ratio values less than each given threshold value in sequence, counting up from zero. Above the last threshold value, integer copy numbers are called assuming full purity, diploidy, and rounding up.

Default thresholds follow this heuristic for calling CNAs in a tumor sample: For single-copy gains and losses, assume 50% tumor cell clonality (including normal cell contamination). Then:

```
R> log2(2:6 / 4)
-1.0  -0.4150375  0.0  0.3219281  0.5849625
```

Allowing for random noise of +/- 0.1, the cutoffs are:

```
DEL(0)  <  -1.1
LOSS(1) <  -0.25
GAIN(3) >=  +0.2
AMP(4)  >=  +0.7
```

For germline samples, better precision could be achieved with:

```
LOSS(1) <  -0.4
GAIN(3) >=  +0.3
```

cnvlib.call.**round_log2_ratios**(*cnarr*, *absolutes*, *ploidy*, *is_reference_male*, *min_abs_val=0.001*)
Convert absolute integer copy numbers to log2 ratios.

Account for reference gender & ploidy of sex chromosomes.

coverage

Supporting functions for the 'antitarget' command.

cnvlib.coverage.**bam_total_reads**(*bam_fname*)
Count the total number of mapped reads in a BAM file.

Uses the BAM index to do this quickly.

cnvlib.coverage.**bedcov**(*bed_fname*, *bam_fname*, *min_mapq*)
Calculate depth of all regions in a BED file via samtools (pysam) bedcov.

i.e. mean pileup depth across each region.

cnvlib.coverage.**interval_coverages**(*bed_fname*, *bam_fname*, *by_count*, *min_mapq*)
Calculate log2 coverages in the BAM file at each interval.

cnvlib.coverage.**interval_coverages_count**(*bed_fname*, *bam_fname*, *min_mapq*)
Calculate log2 coverages in the BAM file at each interval.

cnvlib.coverage.**interval_coverages_pileup**(*bed_fname*, *bam_fname*, *min_mapq*)
Calculate log2 coverages in the BAM file at each interval.

cnvlib.coverage.**region_depth_count**(*bamfile*, *chrom*, *start*, *end*, *min_mapq*)
Calculate depth of a region via pysam count.

i.e. counting the number of read starts in a region, then scaling for read length and region width to estimate depth.

Coordinates are 0-based, per pysam.

diagram

Chromosome diagram drawing functions.

This uses and abuses Biopython's BasicChromosome module. It depends on ReportLab, too, so we isolate this functionality here so that the rest of CNVkit will run without it. (And also to keep the codebase tidy.)

cnvlib.diagram.**bc_chromosome_draw_label**(*self*, *cur_drawing*, *label_name*)
Monkeypatch to Bio.Graphics.BasicChromosome.Chromosome._draw_label.

Draw a label for the chromosome. Mod: above the chromosome, not below.

cnvlib.diagram.**bc_organism_draw**(*org*, *title*, *wrap=12*)
Modified copy of Bio.Graphics.BasicChromosome.Organism.draw.

Instead of stacking chromosomes horizontally (along the x-axis), stack rows vertically, then proceed with the chromosomes within each row.

Arguments:

- title: The output title of the produced document.

cnvlib.diagram.**build_chrom_diagram**(*features*, *chr_sizes*, *sample_id*)
Create a PDF of color-coded features on chromosomes.

cnvlib.diagram.**create_diagram**(*cnarr*, *segarr*, *threshold*, *min_probes*, *outfname*, *male_reference*)
Create the diagram.

export

Export CNVkit objects and files to other formats.

cnvlib.export.**create_chrom_ids**(*segments*)
Map chromosome names to integers in the order encountered.

cnvlib.export.**export_bed**(*segments*, *ploidy*, *is_reference_male*, *is_sample_female*, *label*, *show*)
Convert a copy number array to a BED-like DataFrame.

For each region in each sample (possibly filtered according to *show*), the columns are:

- reference sequence name
- start (0-indexed)
- end
- sample name or given label
- integer copy number

By default (show="ploidy"), skip regions where copy number is the default ploidy, i.e. equal to 2 or the value set by –ploidy. If show="variant", skip regions where copy number is neutral, i.e. equal to the reference ploidy on autosomes, or half that on sex chromosomes.

cnvlib.export.**export_nexus_basic**(*sample_fname*)
Biodiscovery Nexus Copy Number "basic" format.

Only represents one sample per file.

cnvlib.export.**export_nexus_ogt**(*sample_fname*, *vcf_fname*)
Biodiscovery Nexus Copy Number "Custom-OGT" format.

To create the b-allele frequencies column, alterate allele frequencies from the VCF are aligned to the .cnr file bins. Bins that contain no variants are left blank; if a bin contains multiple variants, then the frequencies are all "mirrored" to be above .5, then the median of those values is taken.

cnvlib.export.**export_seg**(*sample_fnames*)
SEG format for copy number segments.

Segment breakpoints are not the same across samples, so samples are listed in serial with the sample ID as the left column.

cnvlib.export.**export_theta**(*tumor*, *reference*)
Convert tumor segments and normal .cnr or reference .cnn to THetA input.

Follows the THetA segmentation import script but avoid repeating the pileups, since we already have the mean depth of coverage in each target bin.

The options for average depth of coverage and read length do not matter crucially for proper operation of THetA; increased read counts per bin simply increase the confidence of THetA's results.

THetA2 input format is tabular, with columns: ID, chrm, start, end, tumorCount, normalCount

where chromosome IDs ("chrm") are integers 1 through 24.

`cnvlib.export.`**`export_vcf`**(*segments*, *ploidy*, *is_reference_male*, *is_sample_female*, *sample_id=None*)
Convert segments to Variant Call Format.

For now, only 1 sample per VCF. (Overlapping CNVs seem tricky.)

Spec: https://samtools.github.io/hts-specs/VCFv4.2.pdf

`cnvlib.export.`**`fmt_cdt`**(*sample_ids*, *table*)
Format as CDT.

`cnvlib.export.`**`fmt_gct`**(*sample_ids*, *table*)

`cnvlib.export.`**`fmt_jtv`**(*sample_ids*, *table*)
Format for Java TreeView.

`cnvlib.export.`**`format_theta_row`**(*seg*, *cnarr*, *chrom_id*, *log2_to_count*)
Convert a segment's info to a row of THetA input.

For the normal/reference bin count, take the mean of the bin values within each segment so that segments match between tumor and normal.

`cnvlib.export.`**`merge_samples`**(*filenames*)
Merge probe values from multiple samples into a 2D table (of sorts).

Input: dict of {sample ID: (probes, values)}

Output: list-of-tuples: (probe, log2 coverages...)

`cnvlib.export.`**`segments2vcf`**(*segments*, *ploidy*, *is_reference_male*, *is_sample_female*)
Convert copy number segments to VCF records.

fix

Supporting functions for the 'fix' command.

`cnvlib.fix.`**`apply_weights`**(*cnarr*, *ref_matched*, *epsilon=0.0001*)
Calculate weights for each bin.

Weights are derived from:

- bin sizes
- average bin coverage depths in the reference
- the "spread" column of the reference.

`cnvlib.fix.`**`center_by_window`**(*cnarr*, *fraction*, *sort_key*)
Smooth out biases according to the trait specified by sort_key.

E.g. correct GC-biased probes by windowed averaging across similar-GC probes; or for similar interval sizes.

`cnvlib.fix.`**`edge_gains`**(*target_sizes*, *gap_sizes*, *insert_size*)
Calculate coverage gain from neighboring baits' flanking reads.

Letting i = insert size, t = target size, g = gap to neighboring bait, the gain of coverage due to a nearby bait, if g < i, is:

```
(i-g)^2 / 4it
```

If the neighbor flank extends beyond the target (t+g < i), reduce by:

```
(i-t-g)^2 / 4it
```

If a neighbor overlaps the target, treat it as adjacent (gap size 0).

cnvlib.fix.**edge_losses**(*target_sizes*, *insert_size*)
Calculate coverage losses at the edges of baited regions.

Letting i = insert size and t = target size, the proportional loss of coverage near the two edges of the baited region (combined) is:

```
i/2t
```

If the "shoulders" extend outside the bait $(t < i), reduce by:

```
(i-t)^2 / 4it
```

on each side, or (i-t)^2 / 2it total.

cnvlib.fix.**get_edge_bias**(*cnarr*, *margin*)
Quantify the "edge effect" of the target tile and its neighbors.

The result is proportional to the change in the target's coverage due to these edge effects, i.e. the expected loss of coverage near the target edges and, if there are close neighboring tiles, gain of coverage due to "spill over" reads from the neighbor tiles.

(This is not the actual change in coverage. This is just a tribute.)

cnvlib.fix.**load_adjust_coverages**(*pset*, *ref_pset*, *fix_gc*, *fix_edge*, *fix_rmask*)
Load and filter probe coverages; correct using reference and GC.

cnvlib.fix.**mask_bad_probes**(*probes*)
Flag the probes with excessively low or inconsistent coverage.

Returns a bool array where True indicates probes that failed the checks.

cnvlib.fix.**match_ref_to_probes**(*ref_pset*, *probes*)
Filter the reference probes to match the target or antitarget probe set.

importers

Import from other formats to the CNVkit format.

cnvlib.importers.**find_picard_files**(*file_and_dir_names*)
Search the given paths for 'targetcoverage' CSV files.

Per the convention we use in our Picard applets, the target coverage file names end with '.targetcoverage.csv'; anti-target coverages end with '.antitargetcoverage.csv'.

cnvlib.importers.**import_picard_pertargetcoverage**(*fname*)
Parse a Picard CalculateHsMetrics PER_TARGET_COVERAGE file.

Return a CopyNumArray.

Input column names: chrom (str), start, end, length (int), name (str), %gc, mean_coverage, normalized_coverage (float)

`cnvlib.importers.`**`import_seg`**(*segfname*, *chrom_names*, *chrom_prefix*, *from_log10*)

Parse a SEG file as an iterable of CopyNumArray instances.

chrom_names**:** Map (string) chromosome IDs to names. (Applied before chrom_prefix.) e.g. {'23': 'X', '24': 'Y', '25': 'M'}

chrom_prefix**: prepend this string to chromosome names** (usually 'chr' or None)

from_log10: Convert values from log10 to log2.

`cnvlib.importers.`**`parse_theta_results`**(*fname*)

Parse THetA results into a data structure.

Columns: NLL, mu, C, p*

`cnvlib.importers.`**`unpipe_name`**(*name*)

Fix the duplicated gene names Picard spits out.

Return a string containing the single gene name, sans duplications and pipe characters.

Picard CalculateHsMetrics combines the labels of overlapping intervals by joining all labels with '|', e.g. 'BRAF|BRAF' – no two distinct targeted genes actually overlap, though, so these dupes are redundant. Meaningless target names are dropped, e.g. 'CGH|FOO|-' resolves as 'FOO'. In case of ambiguity, the longest name is taken, e.g. "TERT|TERT Promoter" resolves as "TERT Promoter".

reference

Supporting functions for the 'reference' command.

`cnvlib.reference.`**`bed2probes`**(*bed_fname*)

Create neutral-coverage probes from intervals.

`cnvlib.reference.`**`calculate_gc_lo`**(*subseq*)

Calculate the GC and lowercase (RepeatMasked) content of a string.

`cnvlib.reference.`**`combine_probes`**(*filenames*, *fa_fname*, *is_male_reference*)

Calculate the median coverage of each bin across multiple samples.

Input: List of .cnn files, as generated by 'coverage' or 'import-picard'. *fa_fname*: fil columns for GC and RepeatMasker genomic values.

Returns: A single CopyNumArray summarizing the coverages of the input samples, including each bin's "average" coverage, "spread" of coverages, and genomic GC content.

`cnvlib.reference.`**`get_fasta_stats`**(*probes*, *fa_fname*)

Calculate GC and RepeatMasker content of each bin in the FASTA genome.

`cnvlib.reference.`**`reference2regions`**(*reference*, *coord_only=False*)

Extract iterables of target and antitarget regions from a reference.

Like loading two BED files with ngfrills.parse_regions.

`cnvlib.reference.`**`warn_bad_probes`**(*probes*)

Warn about target probes where coverage is poor.

Prints a formatted table to stderr.

reports

Supports the sub-commands *breaks* and *gainloss*. Supporting functions for the text/tabular-reporting commands.

Namely: breaks, gainloss.

`cnvlib.reports.`**`gainloss_by_gene`**(*probes*, *threshold*, *skip_low=False*)
Identify genes where average bin copy ratio value exceeds *threshold*.

NB: Must shift sex-chromosome values beforehand with shift_xx, otherwise all chrX/chrY genes may be reported gained/lost.

`cnvlib.reports.`**`gainloss_by_segment`**(*probes*, *segments*, *threshold*, *skip_low=False*)
Identify genes where segmented copy ratio exceeds *threshold*.

NB: Must shift sex-chromosome values beforehand with shift_xx, otherwise all chrX/chrY genes may be reported gained/lost.

`cnvlib.reports.`**`get_breakpoints`**(*intervals*, *segments*, *min_probes*)
Identify CBS segment breaks within the targeted intervals.

`cnvlib.reports.`**`get_gene_intervals`**(*all_probes*, *ignore=('-', '.', 'CGH')*)
Tally genomic locations of each targeted gene.

Return a dict of chromosomes to a list of tuples: (gene name, start, end).

`cnvlib.reports.`**`group_by_genes`**(*probes*, *skip_low*)
Group probe and coverage data by gene.

Return an iterable of genes, in chromosomal order, associated with their location and coverages:

[(gene, chrom, start, end, [coverages]), ...]

segmentation

Segmentation of copy number values.

`cnvlib.segmentation.`**`do_segmentation`**(*cnarr*, *method*, *threshold=None*, *variants=None*, *skip_low=False*, *skip_outliers=10*, *save_dataframe=False*, *rlibpath=None*)
Infer copy number segments from the given coverage table.

`cnvlib.segmentation.`**`drop_outliers`**(*cnarr*, *width*, *factor*)
Drop outlier bins with log2 ratios too far from the trend line.

Outliers are the log2 values *factor* times the 90th quantile of absolute deviations from the rolling average, within a window of given *width*. The 90th quantile is about 1.2 standard deviations if the log2 values are Gaussian, so this is similar to calling outliers 'factor'*1.2 standard deviations from the rolling mean. For a window size of 50, the breakdown point is 5 outliers within a window, which is plenty robust for our needs.

`cnvlib.segmentation.`**`repair_segments`**(*segments*, *orig_probes*)
Post-process segmentation output.

1. Ensure every chromosome has at least one segment.
2. Ensure first and last segment ends match 1st/last bin ends (but keep log2 as-is).
3. Store probe-level gene names, comma-separated, as the segment name.

`cnvlib.segmentation.`**`seg2cns`**(*seg_text*)
Convert R dataframe contents (SEG) to our native tabular format.

Return a pandas.Dataframe with CNA columns.

`cnvlib.segmentation.`**`squash_segments`**(*seg_pset*)
Combine contiguous segments.

`cnvlib.segmentation.`**`transfer_names_weights`**(*segments*, *cnarr*, *ignore=('-', '.', 'CGH')*)
Copy gene names from *cnarr* to the segmented *segarr*.

Segment name is the comma-separated list of bin gene names.

target

Transform bait intervals into targets more suitable for CNVkit.

`cnvlib.target.`**`add_refflat_names`**(*region_rows*, *refflat_fname*)
Apply RefSeq gene names to a list of targeted regions.

`cnvlib.target.`**`assign_names`**(*region_rows*, *refflat_fname*, *default_name='-'*)
Replace the interval gene names with those at the same loc in refFlat.txt

`cnvlib.target.`**`emit`**(*coords*, *names*)
Try filtering names again. Format the row for yielding.

`cnvlib.target.`**`filter_names`**(*names*, *exclude=('mRNA',)*)
Remove less-meaningful accessions from the given set.

`cnvlib.target.`**`parse_refflat_line`**(*line*)
Parse one line of refFlat.txt; return relevant info.

Pair up the exon start and end positions. Add strand direction to each chromosome as a "+"/"-" suffix (it will be stripped off later).

`cnvlib.target.`**`read_refflat_genes`**(*fname*)
Parse genes; merge those with same name and overlapping regions.

Returns a dict of: {(chrom, strand): [(gene start, gene end, gene name), ...]}

`cnvlib.target.`**`shorten_labels`**(*interval_rows*)
Reduce multi-accession interval labels to the minimum consistent.

So: BED or interval_list files have a label for every region. We want this to be a short, unique string, like the gene name. But if an interval list is instead a series of accessions, including additional accessions for sub-regions of the gene, we can extract a single accession that covers the maximum number of consecutive regions that share this accession.

e.g.

... chr1 879125 879593 + mRNA|JX093079,ens|ENST00000342066,mRNA|JX093077,ref|SAMD11,mRNA|AF161376,mRNA|JX
chr1 880158 880279 + ens|ENST00000483767,mRNA|AF161376,ccds|CCDS3.1,ref|NOC2L ...

becomes:

chr1 879125 879593 + mRNA|AF161376 chr1 880158 880279 + mRNA|AF161376

`cnvlib.target.`**`shortest_name`**(*names*)
Return the shortest trimmed name from the given set.

`cnvlib.target.`**`split_targets`**(*region_rows*, *avg_size*)
Split large tiled intervals into smaller, consecutive targets.

For each of the tiled regions:

- Divide into equal-size (tile_size/avg_size) portions
- Emit the (chrom, start, end) coords of each portion

Bin the regions according to avg_size.

4.1.4 Helper modules

core

CNV utilities.

`cnvlib.core.`**`assert_equal`**(*msg*, ***values*)

Evaluate and compare two or more values for equality.

Sugar for a common assertion pattern. Saves re-evaluating (and retyping) the same values for comparison and error reporting.

Example:

```
>>> assert_equal("Mismatch", expected=1, saw=len(['xx', 'yy']))
...
ValueError: Mismatch: expected = 1, saw = 2
```

`cnvlib.core.`**`check_unique`**(*items*, *title*)

Ensure all items in an iterable are identical; return that one item.

`cnvlib.core.`**`fbase`**(*fname*)

Strip directory and all extensions from a filename.

`cnvlib.core.`**`rbase`**(*fname*)

Strip directory and final extension from a filename.

`cnvlib.core.`**`sorter_chrom`**(*label*)

Create a sorting key from chromosome label.

Sort by integers first, then letters or strings. The prefix "chr" (case-insensitive), if present, is stripped automatically for sorting.

E.g. chr1 < chr2 < chr10 < chrX < chrY < chrM

`cnvlib.core.`**`sorter_chrom_at`**(*index*)

Create a sort key function that gets chromosome label at a list index.

`cnvlib.core.`**`write_dataframe`**(*outfname*, *dframe*, *header=True*)

Write a pandas.DataFrame to a tabular file.

`cnvlib.core.`**`write_text`**(*outfname*, *text*, **more_texts*)

Write one or more strings (blocks of text) to a file.

`cnvlib.core.`**`write_tsv`**(*outfname*, *rows*, *colnames=None*)

Write rows, with optional column header, to tabular file.

metrics

Robust estimators of central tendency and scale.

For use in evaluating performance of copy number estimation.

See: https://en.wikipedia.org/wiki/Robust_measures_of_scale https://astropy.readthedocs.org/en/latest/_modules/astropy/stats/funcs.htm

`cnvlib.metrics.`**`biweight_location`**(*a*, *initial=None*, *c=6.0*, *epsilon=0.0001*)

Compute the biweight location for an array.

The biweight is a robust statistic for determining the central location of a distribution.

`cnvlib.metrics.`**`biweight_midvariance`**(*a*, *initial=None*, *c=9.0*, *epsilon=0.0001*)

Compute the biweight midvariance for an array.

The biweight midvariance is a robust statistic for determining the midvariance (i.e. the standard deviation) of a distribution.

See: https://en.wikipedia.org/wiki/Robust_measures_of_scale#The_biweight_midvariance https://astropy.readthedocs.org/en/latest/_modules/astropy/stats/funcs.html

`cnvlib.metrics.`**`confidence_interval_bootstrap`**(*bins*, *alpha*, *bootstraps=100*)

Confidence interval for segment mean log2 value, estimated by bootstrap.

`cnvlib.metrics.`**`ests_of_scale`**(*deviations*)

Estimators of scale: standard deviation, MAD, biweight midvariance.

Calculates all of these values for an array of deviations and returns them as a tuple.

`cnvlib.metrics.`**`interquartile_range`**(*a*)

Compute the difference between the array's first and third quartiles.

`cnvlib.metrics.`**`median_absolute_deviation`**(*a*, *scale_to_sd=True*)

Compute the median absolute deviation (MAD) of array elements.

The MAD is defined as: `median(abs(a - median(a)))`.

See: https://en.wikipedia.org/wiki/Median_absolute_deviation

`cnvlib.metrics.`**`modal_location`**(*arr*)

Return the modal value of an array's values.

The "mode" is the location of peak density among the values, estimated using a Gaussian kernel density estimator.

arr is a 1-D array of floating-point values, e.g. bin log2 ratio values.

`cnvlib.metrics.`**`prediction_interval`**(*bins*, *alpha*)

Prediction interval, estimated by percentiles.

`cnvlib.metrics.`**`q_n`**(*a*)

Rousseeuw & Croux's (1993) Q_n, an alternative to MAD.

```
Qn := Cn first quartile of (|x_i - x_j|:  i < j)
```

where Cn is a constant depending on n.

Finite-sample correction factors must be used to calibrate the scale of Qn for small-to-medium-sized samples.

n E[Qn] – —– 10 1.392 20 1.193 40 1.093 60 1.064 80 1.048 100 1.038 200 1.019

`cnvlib.metrics.`**`segment_mean`**(*cnarr*, *skip_low=False*)

Weighted average of bin log2 values.

ngfrills

NGS utilities.

`cnvlib.ngfrills.`**`call_quiet`**(**args*)

Safely run a command and get stdout; print stderr if there's an error.

Like subprocess.check_output, but silent in the normal case where the command logs unimportant stuff to stderr. If there is an error, then the full error message(s) is shown in the exception message.

`cnvlib.ngfrills.`**`ensure_path`**(*fname*)
Create dirs and move an existing file to avoid overwriting, if necessary.

If a file already exists at the given path, it is renamed with an integer suffix to clear the way.

`cnvlib.ngfrills.`**`safe_write`**(**args*, ***kwds*)
Write to a filename or file-like object with error handling.

If given a file name, open it. If the path includes directories that don't exist yet, create them. If given a file-like object, just pass it through.

`cnvlib.ngfrills.`**`temp_write_text`**(**args*, ***kwds*)
Save text to a temporary file.

NB: This won't work on Windows b/c the file stays open.

parallel

Utilities for multi-core parallel processing.

class `cnvlib.parallel.`**`SerialPool`**
Bases: `object`

Mimic the multiprocessing.Pool interface, but run in serial.

`apply_async`(*func*, *args*)
Just call the function.

`close`()

`join`()

`cnvlib.parallel.`**`pick_pool`**(*nprocs*)

params

Defines several constants used in the pipeline. Hard-coded parameters for CNVkit. These should not change between runs.

plots

Plotting utilities.

`cnvlib.plots.`**`chromosome_sizes`**(*probes*, *to_mb=False*)
Create an ordered mapping of chromosome names to sizes.

`cnvlib.plots.`**`cnv_on_chromosome`**(*axis*, *probes*, *segments*, *genes*, *background_marker=None*, *do_trend=False*, *y_min=None*, *y_max=None*)
Draw a scatter plot of probe values with CBS calls overlaid.

Argument 'genes' is a list of tuples: (start, end, gene name)

`cnvlib.plots.`**`cnv_on_genome`**(*axis*, *probes*, *segments*, *pad*, *do_trend=False*, *y_min=None*, *y_max=None*)
Plot coverages and CBS calls for all chromosomes on one plot.

`cnvlib.plots.`**`cvg2rgb`**(*cvg*, *desaturate*)
Choose a shade of red or blue representing log2-coverage value.

`cnvlib.plots.`**`gene_coords_by_name`**(*probes*, *names*)
Find the chromosomal position of each named gene in probes.

Returns a dict: {chromosome: [(start, end, gene name), ...]}

`cnvlib.plots.`**`gene_coords_by_range`**(*probes*, *chrom*, *start*, *end*, *ignore=('-', '.', 'CGH')*)
Find the chromosomal position of all genes in a range.

Returns a dict: {chromosome: [(start, end, gene), ...]}

`cnvlib.plots.`**`group_snvs_by_segments`**(*snv_posns*, *snv_freqs*, *segments*, *chrom=None*)
Group SNP allele frequencies by segment.

Return an iterable of: start, end, value.

`cnvlib.plots.`**`parse_range_text`**(*text*)
Parse a chromosomal range specification.

Range spec string should look like: 'chr1:1234-5678'

`cnvlib.plots.`**`partition_by_chrom`**(*chrom_snvs*)
Group the tumor shift values by chromosome (for statistical testing).

`cnvlib.plots.`**`plot_x_dividers`**(*axis*, *chrom_sizes*, *pad*)
Plot vertical dividers and x-axis labels given the chromosome sizes.

Returns a table of the x-position offsets of each chromosome.

Draws vertical black lines between each chromosome, with padding. Labels each chromosome range with the chromosome name, centered in the region, under a tick. Sets the x-axis limits to the covered range.

`cnvlib.plots.`**`setup_chromosome`**(*axis*, *probes=None*, *segments=None*, *variants=None*, *y_min=None*, *y_max=None*, *y_label=None*)
Configure axes for plotting a single chromosome's data.

probes, *segments*, and *variants* should already be subsetted to the region that will be plotted.

`cnvlib.plots.`**`setup_genome`**(*axis*, *probes*, *segments*, *variants*, *y_min=None*, *y_max=None*)
Configure axes for plotting a whole genomes's data.

`cnvlib.plots.`**`snv_on_chromosome`**(*axis*, *variants*, *segments*, *genes*, *do_trend*, *do_boost=False*)

`cnvlib.plots.`**`snv_on_genome`**(*axis*, *variants*, *chrom_sizes*, *segments*, *do_trend*, *pad*, *do_boost=False*)
Plot a scatter-plot of SNP chromosomal positions and shifts.

`cnvlib.plots.`**`test_loh`**(*bins*, *alpha=0.0025*)
Test each chromosome's SNP shifts and the combined others'.

The statistical test is Mann-Whitney, a one-sided non-parametric test for difference in means.

`cnvlib.plots.`**`unpack_range`**(*a_range*)
Extract chromosome, start, end from a string or tuple.

Examples:

> "chr1" -> ("chr1", None, None) "chr1:100-123" -> ("chr1", 100, 123) ("chr1", 100, 123) -> ("chr1", 100, 123)

smoothing

Signal smoothing functions.

`cnvlib.smoothing.`**`check_inputs`**`(`*x*, *width*`)`

Transform width into a half-window size.

width is either a fraction of the length of *x* or an integer size of the whole window. The output half-window size is truncated to the length of *x* if needed.

`cnvlib.smoothing.`**`fit_edges`**`(`*x*, *y*, *wing*, *polyorder=3*`)`

Apply polynomial interpolation to the edges of y, in-place.

Calculates a polynomial fit (of order *polyorder*) of *x* within a window of width twice *wing*, then updates the smoothed values *y* in the half of the window closest to the edge.

`cnvlib.smoothing.`**`outlier_iqr`**`(`*a*, *c=3.0*`)`

Detect outliers as a multiple of the IQR from the median.

By convention, "outliers" are points more than 1.5 * IQR from the median, and "extremes" or extreme outliers are those more than 3.0 * IQR.

`cnvlib.smoothing.`**`outlier_mad_median`**`(`*a*`)`

MAD-Median rule for detecting outliers.

Returns: a boolean array of the same size, where outlier indices are True.

X_i is an outlier if:

```
 | X_i - M |
_____________  > K ~= 2.24

 MAD / 0.6745
```

where $K = sqrt(X^2_{0.975,1})$, the square root of the 0.975 quantile of a chi-squared distribution with 1 degree of freedom.

This is a very robust rule with the highest possible breakdown point of 0.5.

See:

- Davies & Gather (1993) The Identification of Multiple Outliers.
- Rand R. Wilcox (2012) Introduction to robust estimation and hypothesis testing. Ch.3: Estimating measures of location and scale.

`cnvlib.smoothing.`**`rolling_median`**`(`*x*, *width*`)`

Rolling median with mirrored edges.

Contributed by Peter Otten to comp.lang.python. This is (somehow) faster than pandas' Cythonized skip-list implementation for arrays smaller than ~100,000 elements.

Source: https://bitbucket.org/janto/snippets/src/tip/running_median.py https://groups.google.com/d/msg/comp.lang.python/0OAR glW4t6gJ

`cnvlib.smoothing.`**`rolling_outlier_iqr`**`(`*x*, *width*, *c=3.0*`)`

Detect outliers as a multiple of the IQR from the median.

By convention, "outliers" are points more than 1.5 * IQR from the median (~2 SD if values are normally distributed), and "extremes" or extreme outliers are those more than 3.0 * IQR (~4 SD).

`cnvlib.smoothing.`**`rolling_outlier_quantile`**`(`*x*, *width*, *q*, *m*`)`

Return a boolean mask of outliers by multiples of a quantile in a window.

Outliers are the array elements outside *m* times the *q*'th quantile of deviations from the smoothed trend line, as calculated from the trend line residuals. (For example, take the magnitude of the 95th quantile times 5, and mark any elements greater than that value as outliers.)

This is the smoothing method used in BIC-seq (doi:10.1073/pnas.1110574108) with the parameters width=200, q=.95, m=5 for WGS.

`cnvlib.smoothing.`**`rolling_outlier_std`**`(`*x*, *width*, *stdevs*`)`
Return a boolean mask of outliers by stdev within a rolling window.

Outliers are the array elements outside *stdevs* standard deviations from the smoothed trend line, as calculated from the trend line residuals.

`cnvlib.smoothing.`**`rolling_quantile`**`(`*x*, *width*, *quantile*`)`
Rolling quantile (0–1) with mirrored edges.

`cnvlib.smoothing.`**`rolling_std`**`(`*x*, *width*`)`
Rolling quantile (0–1) with mirrored edges.

`cnvlib.smoothing.`**`smoothed`**`(`*x*, *width*, *do_fit_edges=False*`)`
Smooth the values in *x* with the Kaiser windowed filter.

See: https://en.wikipedia.org/wiki/Kaiser_window

Parameters:

x [array-like] 1-dimensional numeric data set.

width [float] Fraction of x's total length to include in the rolling window (i.e. the proportional window width), or the integer size of the window.

CHAPTER

FIVE

CITATION

We are in the process of publishing a manuscript describing CNVkit. If you use this software in a publication, for now, please cite our preprint manuscript by DOI, like so:

> Talevich, E., Shain, A.H., Botton, T., & Bastian, B.C. (2014). CNVkit: Copy number detection and visualization for targeted sequencing using off-target reads. *bioRxiv* doi: http://dx.doi.org/10.1101/010876

A poster presentation can be viewed at F1000 Research.

5.1 Who is using CNVkit?

Google Scholar lists some of the references where CNVkit has been used by other researchers.

We'd like to highlight:

- McCreery, M.Q. *et al.* (2015). Evolution of metastasis revealed by mutational landscapes of chemically induced skin cancers. *Nature Medicine*
- Shain, A.H. *et al.* (2015). Exome sequencing of desmoplastic melanoma identifies recurrent NFKBIE promoter mutations and diverse activating mutations in the MAPK pathway. *Nature Genetics*, 47(10), 1194-1199
- Shain, A.H. *et al.* (2015). The Genetic Evolution of Melanoma from Precursor Lesions. *New England Journal of Medicine*, 373(20), 1926-1936

Specific support for CNVkit is included in bcbio-nextgen, THetA2, and MetaSV; CNVkit can also *export* files to several standard formats that can be used with many other software packages.

CHAPTER

SIX

INDICES AND TABLES

- genindex
- modindex
- search

PYTHON MODULE INDEX

C

cnvlib, 37
cnvlib.antitarget, 43
cnvlib.call, 43
cnvlib.cnary, 39
cnvlib.commands, 41
cnvlib.core, 51
cnvlib.coverage, 44
cnvlib.diagram, 44
cnvlib.export, 45
cnvlib.fix, 46
cnvlib.gary, 37
cnvlib.importers, 47
cnvlib.metrics, 51
cnvlib.ngfrills, 52
cnvlib.parallel, 53
cnvlib.params, 53
cnvlib.plots, 53
cnvlib.rary, 41
cnvlib.reference, 48
cnvlib.reports, 48
cnvlib.segmentation, 49
cnvlib.smoothing, 54
cnvlib.target, 50
cnvlib.vary, 41

A

absolute_clonal() (in module cnvlib.call), 43
absolute_dataframe() (in module cnvlib.call), 43
absolute_pure() (in module cnvlib.call), 43
absolute_threshold() (in module cnvlib.call), 43
add() (cnvlib.gary.GenomicArray method), 37
add_columns() (cnvlib.gary.GenomicArray method), 37
add_refflat_names() (in module cnvlib.target), 50
apply_async() (cnvlib.parallel.SerialPool method), 53
apply_weights() (in module cnvlib.fix), 46
as_columns() (cnvlib.gary.GenomicArray method), 37
as_dataframe() (cnvlib.gary.GenomicArray method), 37
as_rows() (cnvlib.gary.GenomicArray method), 37
assert_equal() (in module cnvlib.core), 51
assign_names() (in module cnvlib.target), 50
autosomes() (cnvlib.gary.GenomicArray method), 38

B

bam_total_reads() (in module cnvlib.coverage), 44
batch_make_reference() (in module cnvlib.commands), 41
batch_run_sample() (in module cnvlib.commands), 41
batch_write_coverage() (in module cnvlib.commands), 42
bc_chromosome_draw_label() (in module cnvlib.diagram), 44
bc_organism_draw() (in module cnvlib.diagram), 44
bed2probes() (in module cnvlib.reference), 48
bedcov() (in module cnvlib.coverage), 44
biweight_location() (in module cnvlib.metrics), 51
biweight_midvariance() (in module cnvlib.metrics), 51
build_chrom_diagram() (in module cnvlib.diagram), 45
by_chromosome() (cnvlib.gary.GenomicArray method), 38
by_gene() (cnvlib.cnary.CopyNumArray method), 39
by_ranges() (cnvlib.gary.GenomicArray method), 38

C

calculate_gc_lo() (in module cnvlib.reference), 48
call_quiet() (in module cnvlib.ngfrills), 52
center_all() (cnvlib.cnary.CopyNumArray method), 40
center_by_window() (in module cnvlib.fix), 46
check_inputs() (in module cnvlib.smoothing), 54
check_unique() (in module cnvlib.core), 51
chromosome (cnvlib.gary.GenomicArray attribute), 38
chromosome_sizes() (in module cnvlib.plots), 53
close() (cnvlib.parallel.SerialPool method), 53
cnv_on_chromosome() (in module cnvlib.plots), 53
cnv_on_genome() (in module cnvlib.plots), 53
cnvlib (module), 37
cnvlib.antitarget (module), 43
cnvlib.call (module), 43
cnvlib.cnary (module), 39
cnvlib.commands (module), 41
cnvlib.core (module), 51
cnvlib.coverage (module), 44
cnvlib.diagram (module), 44
cnvlib.export (module), 45
cnvlib.fix (module), 46
cnvlib.gary (module), 37
cnvlib.importers (module), 47
cnvlib.metrics (module), 51
cnvlib.ngfrills (module), 52
cnvlib.parallel (module), 53
cnvlib.params (module), 53
cnvlib.plots (module), 53
cnvlib.rary (module), 41
cnvlib.reference (module), 48
cnvlib.reports (module), 48
cnvlib.segmentation (module), 49
cnvlib.smoothing (module), 54
cnvlib.target (module), 50
cnvlib.vary (module), 41
combine_probes() (in module cnvlib.reference), 48
concat() (cnvlib.gary.GenomicArray method), 38
confidence_interval_bootstrap() (in module cnvlib.metrics), 52
coords() (cnvlib.gary.GenomicArray method), 38
copy() (cnvlib.gary.GenomicArray method), 38
CopyNumArray (class in cnvlib.cnary), 39
create_chrom_ids() (in module cnvlib.export), 45
create_diagram() (in module cnvlib.diagram), 45
csvstring() (in module cnvlib.commands), 42
cvg2rgb() (in module cnvlib.plots), 53

D

do_access() (in module cnvlib.commands), 42
do_antitarget() (in module cnvlib.commands), 42
do_breaks() (in module cnvlib.commands), 42
do_call() (in module cnvlib.commands), 42
do_coverage() (in module cnvlib.commands), 42
do_fix() (in module cnvlib.commands), 42
do_gainloss() (in module cnvlib.commands), 42
do_heatmap() (in module cnvlib.commands), 42
do_reference() (in module cnvlib.commands), 42
do_reference_flat() (in module cnvlib.commands), 42
do_rescale() (in module cnvlib.commands), 42
do_scatter() (in module cnvlib.commands), 42
do_segmentation() (in module cnvlib.segmentation), 49
do_targets() (in module cnvlib.commands), 42
drop_extra_columns() (cnvlib.gary.GenomicArray method), 38
drop_low_coverage() (cnvlib.cnary.CopyNumArray method), 40
drop_outliers() (in module cnvlib.segmentation), 49

E

edge_gains() (in module cnvlib.fix), 46
edge_losses() (in module cnvlib.fix), 47
emit() (in module cnvlib.target), 50
end (cnvlib.gary.GenomicArray attribute), 38
ensure_path() (in module cnvlib.ngfrills), 52
ests_of_scale() (in module cnvlib.metrics), 52
expect_flat_cvg() (cnvlib.cnary.CopyNumArray method), 40
export_bed() (in module cnvlib.export), 45
export_nexus_basic() (in module cnvlib.export), 45
export_nexus_ogt() (in module cnvlib.export), 45
export_seg() (in module cnvlib.export), 45
export_theta() (in module cnvlib.export), 45
export_vcf() (in module cnvlib.export), 46

F

fbase() (in module cnvlib.core), 51
filter_names() (in module cnvlib.target), 50
find_background_regions() (in module cnvlib.antitarget), 43
find_picard_files() (in module cnvlib.importers), 47
fit_edges() (in module cnvlib.smoothing), 55
fmt_cdt() (in module cnvlib.export), 46
fmt_gct() (in module cnvlib.export), 46
fmt_jtv() (in module cnvlib.export), 46
format_theta_row() (in module cnvlib.export), 46
from_columns() (cnvlib.gary.GenomicArray class method), 38
from_rows() (cnvlib.gary.GenomicArray class method), 38

G

gainloss_by_gene() (in module cnvlib.reports), 48
gainloss_by_segment() (in module cnvlib.reports), 49
gene_coords_by_name() (in module cnvlib.plots), 53
gene_coords_by_range() (in module cnvlib.plots), 54
GenomicArray (class in cnvlib.gary), 37
get_background() (in module cnvlib.antitarget), 43
get_breakpoints() (in module cnvlib.reports), 49
get_edge_bias() (in module cnvlib.fix), 47
get_fasta_stats() (in module cnvlib.reference), 48
get_gene_intervals() (in module cnvlib.reports), 49
get_relative_chrx_cvg() (cnvlib.cnary.CopyNumArray method), 40
group_by_genes() (in module cnvlib.reports), 49
group_snvs_by_segments() (in module cnvlib.plots), 54
guess_average_depth() (cnvlib.cnary.CopyNumArray method), 40
guess_chromosome_regions() (in module cnvlib.antitarget), 43
guess_xx() (cnvlib.cnary.CopyNumArray method), 40

I

import_picard_pertargetcoverage() (in module cnvlib.importers), 47
import_seg() (in module cnvlib.importers), 47
in_range() (cnvlib.gary.GenomicArray method), 38
in_ranges() (cnvlib.gary.GenomicArray method), 38
interquartile_range() (in module cnvlib.metrics), 52
interval_coverages() (in module cnvlib.coverage), 44
interval_coverages_count() (in module cnvlib.coverage), 44
interval_coverages_pileup() (in module cnvlib.coverage), 44

J

join() (cnvlib.parallel.SerialPool method), 53

K

keep_columns() (cnvlib.gary.GenomicArray method), 39

L

labels() (cnvlib.gary.GenomicArray method), 39
load_adjust_coverages() (in module cnvlib.fix), 47
log2 (cnvlib.cnary.CopyNumArray attribute), 40

M

mask_bad_probes() (in module cnvlib.fix), 47
match_ref_to_probes() (in module cnvlib.fix), 47
match_to_bins() (cnvlib.gary.GenomicArray method), 39
median_absolute_deviation() (in module cnvlib.metrics), 52
merge_samples() (in module cnvlib.export), 46
mirrored_baf() (cnvlib.vary.VariantArray method), 41

modal_location() (in module cnvlib.metrics), 52

O

outlier_iqr() (in module cnvlib.smoothing), 55
outlier_mad_median() (in module cnvlib.smoothing), 55

P

parse_args() (in module cnvlib.commands), 42
parse_range_text() (in module cnvlib.plots), 54
parse_refflat_line() (in module cnvlib.target), 50
parse_theta_results() (in module cnvlib.importers), 48
partition_by_chrom() (in module cnvlib.plots), 54
pick_pool() (in module cnvlib.parallel), 53
plot_x_dividers() (in module cnvlib.plots), 54
prediction_interval() (in module cnvlib.metrics), 52
print_version() (in module cnvlib.commands), 42

Q

q_n() (in module cnvlib.metrics), 52

R

rbase() (in module cnvlib.core), 51
read() (cnvlib.gary.GenomicArray class method), 39
read() (cnvlib.rary.RegionArray class method), 41
read() (in module cnvlib), 37
read_refflat_genes() (in module cnvlib.target), 50
read_vcf() (cnvlib.vary.VariantArray class method), 41
reference2regions() (in module cnvlib.reference), 48
region_depth_count() (in module cnvlib.coverage), 44
RegionArray (class in cnvlib.rary), 41
repair_segments() (in module cnvlib.segmentation), 49
residuals() (cnvlib.cnary.CopyNumArray method), 40
rolling_median() (in module cnvlib.smoothing), 55
rolling_outlier_iqr() (in module cnvlib.smoothing), 55
rolling_outlier_quantile() (in module cnvlib.smoothing), 55
rolling_outlier_std() (in module cnvlib.smoothing), 56
rolling_quantile() (in module cnvlib.smoothing), 56
rolling_std() (in module cnvlib.smoothing), 56
round_log2_ratios() (in module cnvlib.call), 44
row2label() (cnvlib.gary.GenomicArray static method), 39

S

safe_write() (in module cnvlib.ngfrills), 53
sample_id (cnvlib.gary.GenomicArray attribute), 39
seg2cns() (in module cnvlib.segmentation), 49
segment_mean() (in module cnvlib.metrics), 52
segments2vcf() (in module cnvlib.export), 46
select() (cnvlib.gary.GenomicArray method), 39
SerialPool (class in cnvlib.parallel), 53
setup_chromosome() (in module cnvlib.plots), 54
setup_genome() (in module cnvlib.plots), 54
shift_xx() (cnvlib.cnary.CopyNumArray method), 40
shorten_labels() (in module cnvlib.target), 50
shortest_name() (in module cnvlib.target), 50
shuffle() (cnvlib.gary.GenomicArray method), 39
smoothed() (in module cnvlib.smoothing), 56
snv_on_chromosome() (in module cnvlib.plots), 54
snv_on_genome() (in module cnvlib.plots), 54
sort() (cnvlib.gary.GenomicArray method), 39
sort_columns() (cnvlib.gary.GenomicArray method), 39
sorter_chrom() (in module cnvlib.core), 51
sorter_chrom_at() (in module cnvlib.core), 51
split_targets() (in module cnvlib.target), 50
squash_genes() (cnvlib.cnary.CopyNumArray method), 40
squash_segments() (in module cnvlib.segmentation), 49
start (cnvlib.gary.GenomicArray attribute), 39

T

temp_write_text() (in module cnvlib.ngfrills), 53
test_loh() (in module cnvlib.plots), 54
transfer_names_weights() (in module cnvlib.segmentation), 49
tumor_boost() (cnvlib.vary.VariantArray method), 41

U

unpack_range() (in module cnvlib.plots), 54
unpipe_name() (in module cnvlib.importers), 48

V

VariantArray (class in cnvlib.vary), 41
verify_gender_arg() (in module cnvlib.commands), 43

W

warn_bad_probes() (in module cnvlib.reference), 48
write() (cnvlib.gary.GenomicArray method), 39
write() (cnvlib.rary.RegionArray method), 41
write_dataframe() (in module cnvlib.core), 51
write_text() (in module cnvlib.core), 51
write_tsv() (in module cnvlib.core), 51
